# The Ruthenium Nitrosyl Moiety in Clusters: Trinuclear Linear μ-Hydroxido Magnesium(II)-Diruthenium(II), μ_3_-Oxido Trinuclear Diiron(III)–Ruthenium(II), and Tetranuclear μ_4_-Oxido Trigallium(III)-Ruthenium(II) Complexes

**DOI:** 10.1021/acs.inorgchem.1c03011

**Published:** 2021-12-28

**Authors:** Iryna Stepanenko, Pavlo Mizetskyi, Ewelina Orlowska, Lukáš Bučinský, Michal Zalibera, Barbora Vénosová, Martin Clémancey, Geneviève Blondin, Peter Rapta, Ghenadie Novitchi, Wolfgang Schrader, Dominik Schaniel, Yu-Sheng Chen, Martin Lutz, Jozef Kožíšek, Joshua Telser, Vladimir B. Arion

**Affiliations:** † University of Vienna, Institute of Inorganic Chemistry, Währinger Strasse 42, A-1090 Vienna, Austria; ‡ Institute of Physical Chemistry and Chemical Physics, Faculty of Chemical and Food Technology, Slovak University of Technology in Bratislava, Radlinského 9, SK-81237 Bratislava, Slovak Republic; § Univ. Grenoble Alpes, CNRS, CEA, IRIG, LCBM, F-38000 Grenoble, France; ∥ CNRS-LNCMI, 17 avenue des Martyrs, 38042 Grenoble Cedex, France; ⊥ MPI für Kohlenforschung, Kaiser-Wilhelm-Platz 1, 45470 Mülheim an der Ruhr, Germany; Δ Université de Lorraine, CNRS, CRM2, 54506 Nancy, France; ∇ NSF’s ChemMATCARS, The University of Chicago, Lemont, Illinois 60439, United States; △ Structural Biochemistry, Bijvoet Centre for Biomolecular Research, Utrecht University, 3584 CH Utrecht, The Netherlands; ○ Department of Biological, Physical and Health Sciences, Roosevelt University, 430 South Michigan Avenue, Chicago, Illinois 60605, United States; ⬡ Department of Physics, Faculty of Science, University of Ostrava, 30. dubna 22, 70103 Ostrava, Czech Republic

## Abstract

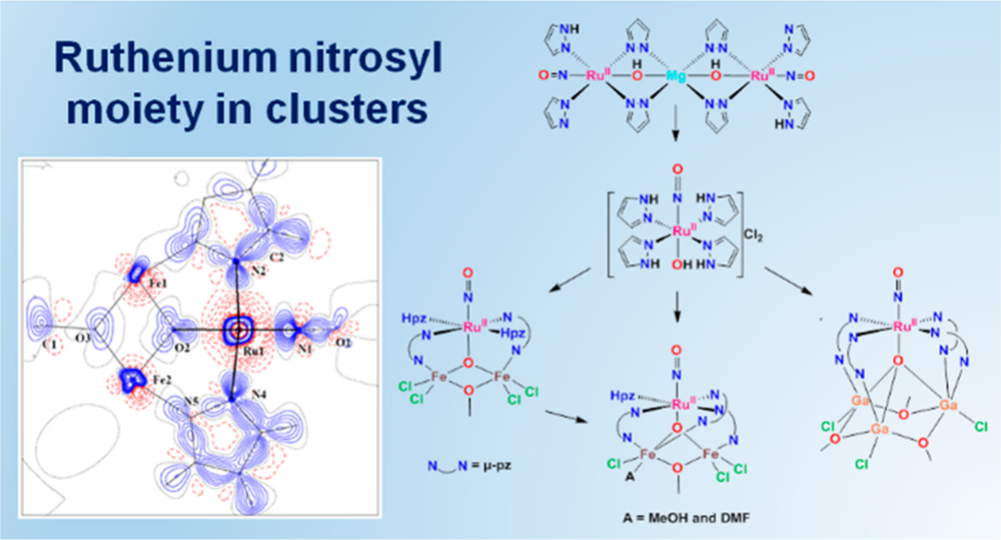

The ruthenium nitrosyl moiety, {RuNO}^6^, is important as a potential releasing agent of nitric oxide and is of inherent interest in coordination chemistry. Typically, {RuNO}^6^ is found in mononuclear complexes. Herein we describe the synthesis and characterization of several multimetal cluster complexes that contain this unit. Specifically, the heterotrinuclear μ_3_-oxido clusters [Fe_2_RuCl_4_(μ_3_-O)(μ-OMe)(μ-pz)_2_(NO)(Hpz)_2_] (**6**) and [Fe_2_RuCl_3_(μ_3_-O)(μ-OMe)(μ-pz)_3_(MeOH)(NO)(Hpz)][Fe_2_RuCl_3_(μ_3_-O)(μ-OMe)(μ-pz)_3_(DMF)(NO)(Hpz)] (**7**·MeOH·2H_2_O) and the heterotetranuclear μ_4_-oxido complex [Ga_3_RuCl_3_(μ_4_-O)(μ-OMe)_3_(μ-pz)_4_(NO)] (**8**) were prepared from *trans*-[Ru(OH)(NO)(Hpz)_4_]Cl_2_ (**5**), which itself was prepared via acidic hydrolysis of the linear heterotrinuclear complex {[Ru(μ-OH)(μ-pz)_2_(pz)(NO)(Hpz)]_2_Mg} (**4**). Complex **4** was synthesized from the mononuclear Ru complexes (H_2_pz)[*trans*-RuCl_4_(Hpz)_2_] (**1**), *trans*-[RuCl_2_(Hpz)_4_]Cl (**2**), and *trans*-[RuCl_2_(Hpz)_4_] (**3**). The new compounds **4**–**8** were all characterized by elemental analysis, ESI mass spectrometry, IR, UV–vis, and ^1^H NMR spectroscopy, and single-crystal X-ray diffraction, with complexes **6** and **7** being characterized also by temperature-dependent magnetic susceptibility measurements and Mössbauer spectroscopy. Magnetometry indicated a strong antiferromagnetic interaction between paramagnetic centers in **6** and **7**. The ability of **4** and **6**–**8** to form linkage isomers and release NO upon irradiation in the solid state was investigated by IR spectroscopy. A theoretical investigation of the electronic structure of **6** by DFT and *ab initio* CASSCF/NEVPT2 calculations indicated a redox-noninnocent behavior of the NO ancillary ligand in **6**, which was also manifested in TD-DFT calculations of its electronic absorption spectrum. The electronic structure of **6** was also studied by an X-ray charge density analysis.

## Abstract 2

Mononuclear *trans-*[Ru(OH)NO(Hpz)_4_]^2+^ proved to be a source of μ-hydroxido and μ_3_- and/or μ_4_-oxido bridging groups, which could be incorporated into the heterotrinuclear complexes {[Ru(μ-OH)(μ-pz)_2_(pz)(NO)(Hpz)]_2_Mg}, [Fe_2_RuCl_4_(μ_3_-O)(μ-OMe)(μ-pz)_2_(NO)(Hpz)_2_], and [Fe_2_RuCl_3_(μ_3_-O)(μ-OMe)(μ-pz)_3_(MeOH)(NO)(Hpz)][Fe_2_RuCl_3_(μ_3_-O)(μ-OMe)(μ-pz)_3_(DMF)(NO)(Hpz)] (**7**·MeOH·2H_2_O) and the heterotetranuclear μ_4_-oxido complex [Ga_3_RuCl_3_(μ_4_-O)(μ-OMe)_3_(μ-pz)_4_(NO)]. The structures obtained were all confirmed by SC-XRD, including an X-ray charge density analysis that revealed the electronic structure of the RuFe2 cluster. Two of these nitrosyl complexes underwent photoinduced isomerization with generation of the nitrosyl linkage isomers MS1 and MS2, as revealed by IR spectroscopy at 10 K.

## Introduction

**Chart 1:**
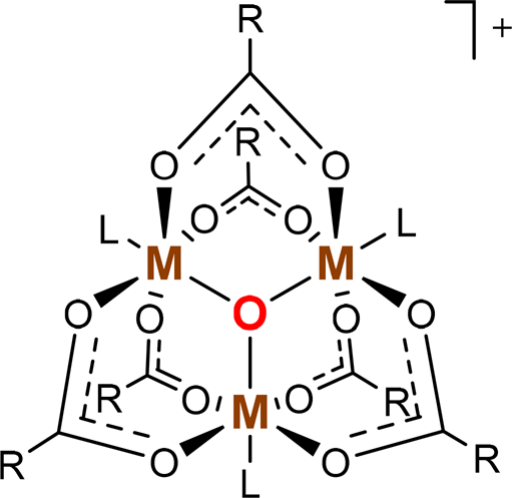
μ_3_-Oxido-Bridged Trinuclear Metal Carboxylatesa

μ_3_-Oxido-centered transition metal based triangular complexes form a class of coordination compounds with many members, often exhibiting exciting electronic and magnetic properties.^1−11^ The trinuclear μ_3_-oxido cluster with μ_2_-carboxylate bridges is a well-known structural type within the broader class of complexes with a [M_3_(μ_3_-O)]^*n*+^ core. A ConQuest search in the Cambridge Structural Database resulted in 220 hits of unique compounds with structures of [Fe^III^_3_(μ_3_-O)(μ_2_-O_2_CR)_6_X_3_]^+^ (X = neutral coligand; none with five-coordinate iron(III)) and 55 hits of unique structures that are [Ru^III^_3_(μ_3_-O)(μ_2_-O_2_CR)_6_X_3_]^+^ (Chart 1). Mixed-valence μ_3_-oxido transition metal carboxylates have attracted much attention as well: e.g., iron(III,III,II) complexes were found to be suitable for an investigation of intramolecular electron transfer in the solid state.^12,13^

a Here with μ_2_-carboxylate bridges and generic axial ligands, L.

The nature of the bridging and axial ligands (Chart 1), the second coordination sphere, the crystallographic phase transition related to the onset of an interstitial solvent, axial ligand L, and/or counteranion motion at a certain temperature were suggested to affect the rate of electron transfer in these mixed-valence iron complexes.^14−16^ Even though heterotrinuclear 3d metal complexes with haloacetate ligands of the type [M^III^_2_M^II^(μ_3_-O)(μ_2_-O_2_CR)_6_(H_2_O)_3_], where M^III^ = Fe, Ru, M^II^ = VO, Mn, Fe, Co, Ni, Cu, Zn, and R = CH_2_Cl, CH_2_Br, CH_2_I, CCl_3_, were reported,^17,18^ there were no hits in a CSD search for heteronuclear 1Fe,2Ru or 2Fe,1Ru clusters. Therefore, the intra group 8 aspect of metal carboxylate chemistry is worth exploring and has motivated the present work.

**Chart 2:**
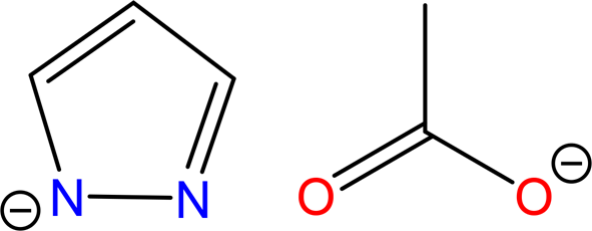
Pyrazolate and Carboxylate as Potential Bridging Ligands

Recently a structural parallel between carboxylate and pyrazolate complexes of the same nuclearity has been recognized (i.e., μ_2_-O=C–O and μ_2_-N–N–; see Chart 2) and several multinuclear pyrazolate systems have been synthesized and comprehensively characterized.^19−22^

It should also be stressed that the source of the μ_3_-oxido bridging group in metal carboxylates, and several pyrazolates, is likely water and/or hydroxide ions introduced into the reaction mixture whether in a controlled or an uncontrolled manner. We hypothesized that an octahedral tetrapyrazole ruthenium complex bearing NO and OH^–^ coligands in axial positions could thus also be a suitable source of μ-hydroxido and μ_3_- or μ_4_-oxido groups that might be incorporated into more developed heteropolynuclear metal complexes. The idea arose from a simple experiment wherein *trans*-[Ru(OH)(NO)(Hpz)_4_]Cl_2_ was treated with excess Ba(OH)_2_ in methanol and the solution subjected to high-resolution electrospray ionization mass spectrometry (HR ESI MS). The mass spectra in positive ion mode showed peaks that could be attributed to [{Ru(OH)(pz)_3_(NO)(Hpz)}_2_M^III^]^+^ (M = Fe, Al) and to [{Ru(OH)(pz)_3_(NO)(Hpz)}_2_Ba+H]^+^ (see Figures S1–S3 in the Supporting Information), all containing oxophilic metal ions present in-source. The use of ESI MS as the technique of choice for the study of gas-phase coordination chemistry and for rapid screening of the reactivity of metal complexes is well-documented.^23,24^ The characteristic multi-isotopic distribution pattern of ruthenium in combination with high resolution makes such screening efforts toward the preparation of heterometallic Ru-containing species particularly attractive. These microscale ESI MS experiments were thus used as a prelude to the macroscale synthesis of heteronuclear complexes of oxophilic metal ions.

Ruthenium nitrosyl complexes show interesting redox activity^25,26^ and are of inherent interest as potential anticancer drugs.^27−29^ As discussed in a recent “Forum of Renaissance in NO Chemistry”,^30^ nitric oxide release both in the solid state and in aqueous solution is relevant for biomedical applications.^31,32^ Light-induced Ru–NO bond dissociation in solution^33^ may involve the formation of the intermediate metastable linkage isomers MS1 (Ru-ON) and MS2 (Ru-η^2^-NO).^34−37^ These photoisomerization processes in the solid state have potential uses in data storage^38−40^ and in the design of photochromic materials^41^ or hologram gratings on the basis of the difference in refractive indexes between the ground and metastable states.^42^ Another interest is the use of ruthenium nitrosyl complexes as building blocks for the assembly of systems combining a photoswitchable unit with a paramagnetic moiety in the same crystal.^40,43,44^ Such systems may allow for light-induced linkage isomerization, which will trigger changes in magnetic behavior, leading potentially to novel functional materials. In particular, the ruthenium complexes [RuNO(NH_3_)_5_]^3+^, [RuNO(NH_3_)_4_OH]^2+^, and [RuNO(en)_2_Cl]^2+^ with paramagnetic anions based on chromium, cobalt, and manganese have been synthesized and characterized.^43,44^ The light-induced transition of the ground state (GS) Ru-NO unit to its metastable isomer Ru-ON (MS1) was observed in all cases. MS1 is stable only at low temperature, although an increase in back-isomerization (MS1 to GS) temperature of 20–25 K was found, depending on the identity of the counteranion. For [RuNO(NH_3_)_5_][Cr(CN)_6_] a weak reversible change of χ_M_*T* value (0.5–1%) after irradiation was observed.^44^ Di- and polynuclear homo- and heterometallic complexes based on ruthenium nitrosyl complexes are still rare.^29,45−48^ Some of the reported systems lose their photoactivity and do not show the formation of metastable states, while other complexes undergo photoisomerization.^49^

Taking all this into account, we aimed at (i) the development of a straightforward approach to new di- and polynuclear homo- and heterometallic ruthenium nitrosyls with desirable properties to produce a complex with a more direct electronic coupling that potentially would generate a larger photomagnetic effect, (ii) the use of Fe^3+^ and Ga^3+^ as oxophilic metal ions for the assembly of molecules combining a photoswitchable unit with paramagnetic ions in the same molecule and heterometallic complexes, which might serve as suitable precursors for the introduction of other paramagnetic metal ions via transmetalation reactions in future studies, respectively, (iii) the investigation of photoisomerization of NO and its photorelease by new heterometallic complexes under light irradiation in the solid state, and (iv) the estimation of the potential of new compounds for NO photorelease in solution in the context that mononuclear ruthenium-nitrosyl complexes containing four equatorial azole heterocycles (i.e., *trans*-[Ru(NO)(X)(1*H*-indazole)_4_]Cl_2_ (X = Cl, OH) were found by us recently to be stable in aqueous solution and exhibit NO photorelease upon UV light irradiation.^33^

**Scheme 1:**
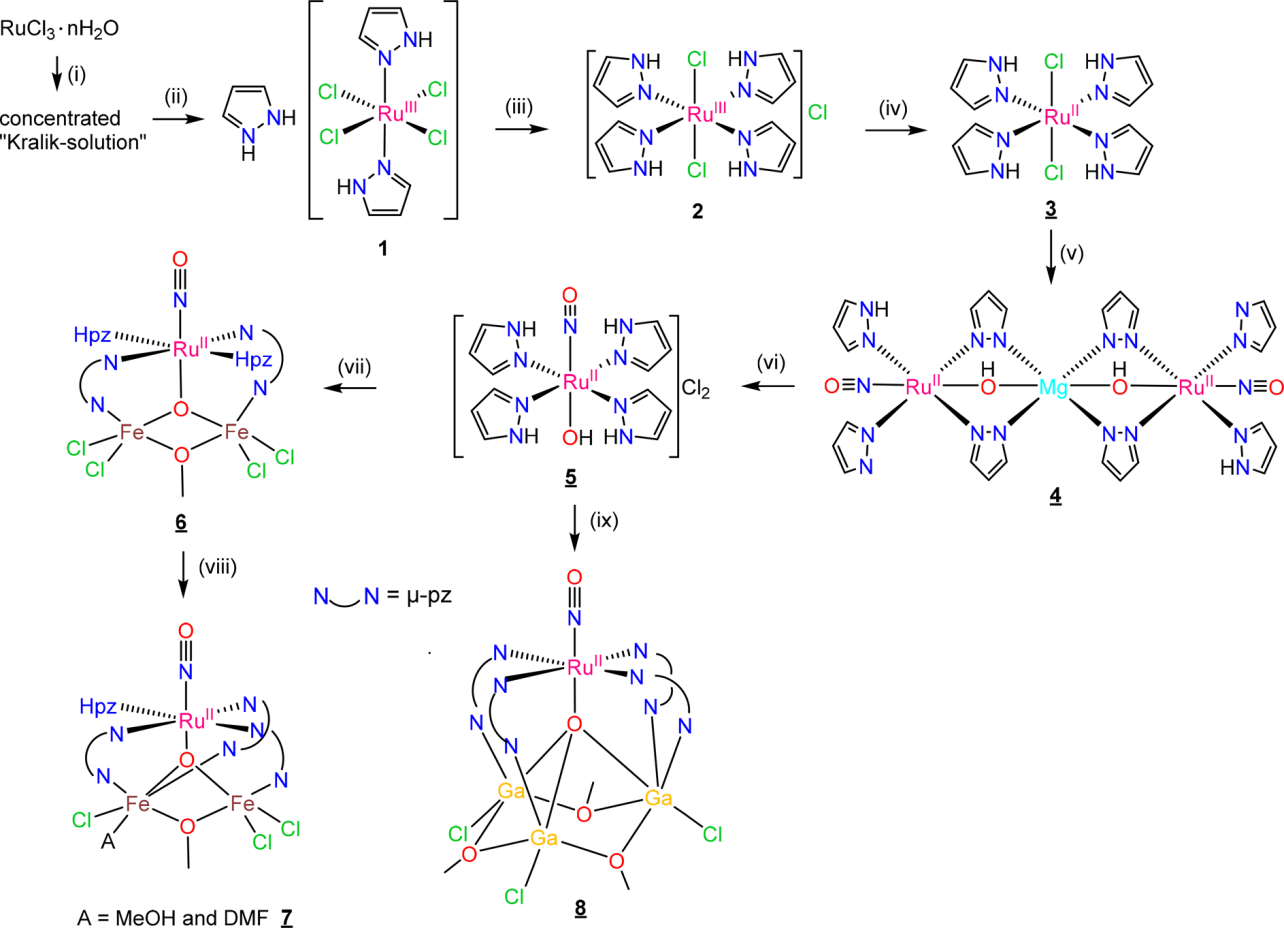
Overview of Reaction Pathways to Complexes **4**–**8** Underlined numbers indicate compounds studied by SC-XRD. Reagents and conditions: (i) EtOH, 12 M HCl (v/v 1/1), reflux, 1 h; (ii) 12 M HCl, excess of 1*H*-pyrazole, rt, 3 days; (iii) H_2_O/EtOH (v/v 3/7), 1*H*-pyrazole, reflux, 7 h; (iv) MeOH, NaBH_4_, rt, 1 h; (v) NaNO_2_, Mg(OH)_2_, acetone/CH_2_Cl_2_ (v/v 1/1), reflux, overnight; (vi) acetone, 3 M HCl; (vii) MeOH, excess FeCl_3_·6H_2_O, K_2_CO_3_, 70 °C, overnight; (viii) addition of several drops of DMF to the solution of **6**; (ix) MeOH, excess GaCl_3_, K_2_CO_3_, 70 °C, 1.5 h.

Herein we report on the synthesis and characterization of a series of such heterometallic Ru cluster complexes: the linear heterotrinuclear μ-hydroxido diruthenium nitrosyl magnesium(II) complex {[Ru(μ-OH)(μ-pz)_2_(pz)(NO)(Hpz)]_2_Mg} (**4**), heterotrinuclear μ_3_-oxido clusters [Fe_2_RuCl_4_(μ_3_-O)(μ-OMe)(μ-pz)_2_(NO)(Hpz)_2_] (**6**) and [Fe_2_RuCl_3_(μ_3_-O)(μ-OMe)(μ-pz)_3_(MeOH)(NO)(Hpz)][Fe_2_RuCl_3_(μ_3_-O)(μ-OMe)(μ-pz)_3_(DMF)(NO)(Hpz)] (**7**·MeOH·2H_2_O), and the heterotetranuclear complex [Ga_3_RuCl_3_(μ_4_-O)(μ-OMe)_3_(μ-pz)_4_(NO)] (**8**) (Scheme 1).

The magnetic behavior and Mössbauer spectra of Fe-containing complexes **6** and **7** are reported. The electronic structure of **6** has been investigated by DFT calculations and X-ray charge density. Finally, and most relevant to potential applications of such nitrosyl complexes, the ability of **4** and **6**–**8** to form photoinduced linkage isomers and release NO upon irradiation in the solid state is described.

## Results and Discussion

### Synthesis

The preparation of a Kralik solution,^50^ and the synthesis of (H_2_pz)[*trans*-Ru^III^Cl_4_(Hpz)_2_] (**1**) and *trans*-[Ru^III^Cl_2_(Hpz)_4_]Cl (**2**)^51,52^ and their ESI mass spectra (Figures S4 and S5, respectively) are specified in the Supporting Information. The reduction of the paramagnetic complex **2** (*S* = 1/2) was performed with NaBH_4_ in methanol and resulted in the formation of the diamagnetic (*S* = 0) complex *trans*-[Ru^II^Cl_2_(Hpz)_4_] (**3**) (Scheme 1). The diamagnetism of complex **3** was confirmed by a ^1^H NMR spectrum, which exhibited sharp resonances in the usual window of chemical shifts for diamagnetic compounds (see Figure S6 in the Supporting Information). The ESI mass spectrum of **3** in MeCN/MeOH measured in the negative ion mode showed a peak at *m*/*z* 442.89 attributed to [Ru^II^Cl_2_(Hpz)_4_–H]^−^ (Figure S7 in the Supporting Information). The reduction step was also confirmed by SC-XRD of **3** (Figure S8 in the Supporting Information).

By the reaction of **3** with Mg(OH)_2_ and NaNO_2_ the new diruthenium-magnesium-nitrosyl complex {[Ru(μ-OH)(μ-pz)_2_(pz)(NO)(Hpz)]_2_Mg} (**4**) was synthesized. ESI mass spectra of **4** showed peaks at *m*/*z* 861.1 and 859.0 attributed to [M + H]^+^ and [M – H]^−^, respectively (Figures S9 and S10 in the Supporting Information). The ^1^H NMR spectrum of **4** in CDCl_3_ at room temperature showed several broad lines, which became resolved at −40 °C.

The synthesis of [Ru^II^(OH)(NO)(Hpz)_4_]Cl_2_ (**5**) was realized by the treatment of **4** with 3 M hydrochloric acid (see the Experimental Section). The formation of [Ru^II^(OH)(NO)(Hpz)_4_]Cl_2_ (**5**) was confirmed by ESI mass spectra (Figures S11 and S12 in the Supporting Information). Peaks at *m*/*z* 420.17 and 418.01 in the spectra of **5** could be attributed to [Ru(OH)(NO)(pz)(Hpz)_3_]^+^ and [Ru(OH)(NO)(pz)_3_(Hpz)]^−^, respectively. The complex was characterized by ^1^H NMR spectroscopy and could be easily identified by a set of peaks of pyrazole protons with different chemical shifts, as well as by an IR spectrum (Figures S13 and S14 in the Supporting Information).

The reaction of crude **5** (see details in the Supporting Information) with excess FeCl_3_·6H_2_O in the presence of K_2_CO_3_ afforded [Fe_2_RuCl_4_(μ_3_-O)(μ-OMe)(μ-pz)_2_(NO)(Hpz)_2_] (**6**) in 46% yield. ESI MS showed several peaks that could be assigned to fragment ions related to **6**, namely at *m*/*z* 597.92 to [M – (Hpz) – Cl]^+^, at *m*/*z* 631.90 to [M – HCl – Cl]^+^, at *m*/*z* 633.90 to [M – (CH_3_O) – HCl]^+^, and at *m*/*z* 665.85 to [M – HCl – H]^−^ (see Figures S15 and S16 in the Supporting Information). When a small amount of DMF was used for the crystallization of **6**, the complex [Fe_2_RuCl_3_(μ_3_-O)(μ-OMe)(μ-pz)_3_(MeOH)(NO)(Hpz)][Fe_2_RuCl_3_(μ_3_-O)(μ-OMe)(μ-pz)_3_(DMF)(NO)(Hpz)] (**7**) was formed, as confirmed by SC-XRD (see X-ray Crystallography). ESI MS showed peaks at *m*/*z* 593.9, 599.8, and 629.84 in the positive ion mode which could be assigned to [M – HCl – Cl]^+^, [M – (Hpz) + H]^+^ and [M – Cl]^+^, respectively, while in the negative ion mode the peak at *m*/*z* 665.75 was attributed to [M – H]^−^, where M is the unit [Fe_2_Ru^II^Cl_3_(μ_3_-O)(μ-OMe)(μ-pz)_3_(NO)(Hpz)] (Figure S17 in the Supporting Information). The IR spectra for **6** and **7**·MeOH·2H_2_O are shown in Figures S18 and S19 in the Supporting Information.

Addition of excess GaCl_3_ instead of FeCl_3_·6H_2_O to **5** gave rise to [Ga_3_RuCl_3_(μ_4_-O)(μ-OMe)_3_(μ-pz)_4_(NO)] (**8**). ESI MS revealed peaks at *m*/*z* 759.80 and 793.76 in the positive ion mode and a signal at *m*/*z* 859.56 in the negative ion mode, attributed to [M – (OMe) – HCl]^+^, [M – (OMe)]^+^, and (M + Cl)^−^, respectively (see Figures S20 and S21), while its IR spectrum is shown in Figure S22 in the Supporting Information. In contrast to **6**, complex **8** could be crystallized in DMF/MeOH and remains intact, as confirmed by SC-XRD measurements (see X-ray Crystallography).

The stretching vibration of the NO ligand is found at 1847, 1881, 1857, and 1862 cm^–1^ in **4** and **6**–**8**, respectively. The presence of DMF coordinated to ruthenium in **7** is corroborated by a C=O stretching vibration at 1646 cm^–1^ (Figure S19 in the Supporting Information).

Complexes **6** and **7** were found to decompose in solution, as confirmed by X-band (9.43 GHz) EPR spectroscopy in frozen MeOH and DMF, which revealed intense signals typical of high-spin rhombic (*S* = 5/2) Fe^III^ at *g* = 4.3 (data not shown).

### X-ray Crystallography

**Figure 1:**
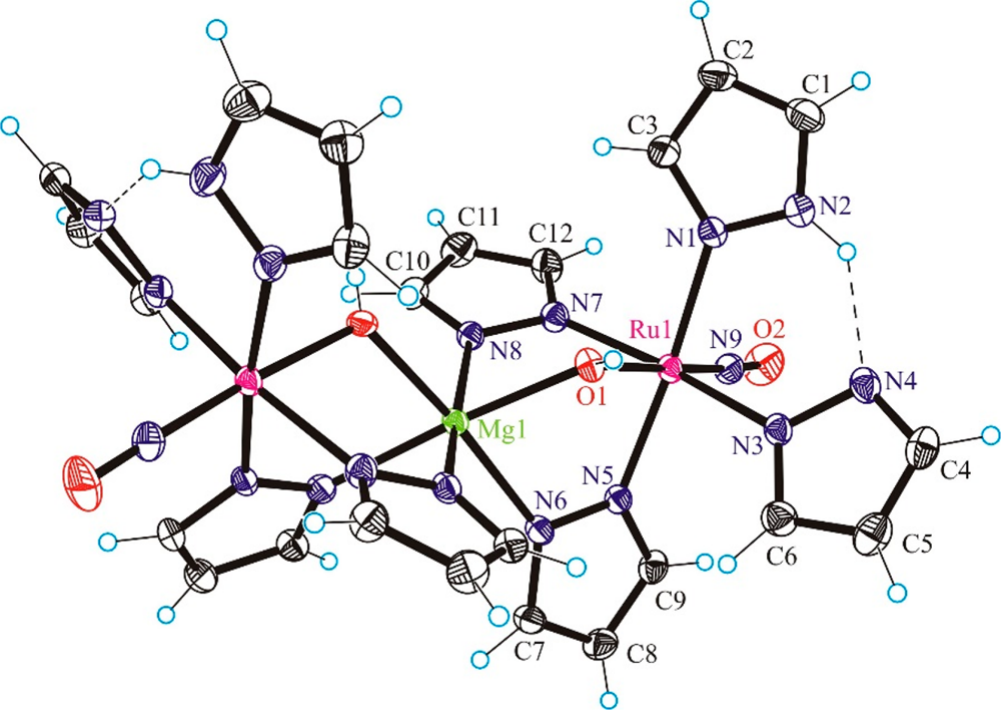
Structure of {[Ru(μ-OH)(μ-pz)_2_(pz)(NO)(Hpz)]_2_Mg} (**4**). Selected bond distances (Å) and bond angles (deg): Ru1–O1 1.9672(12), Ru1–N9 1.7428(16), Ru–N1 2.0873(15), Ru–N3 2.0608(14), Ru–N5 2.0495(15), Ru–N7 2.0772(14), Ru1···Mg 3.37114(17), Mg–O1 2.0347(12), Mg–N6 2.1712(15), Mg–N8 2.1622(14), N9–O2 1.150(2); N9–Ru–O1 176.48(6), O2–N9–Ru 178.05(16).

**Figure 2:**
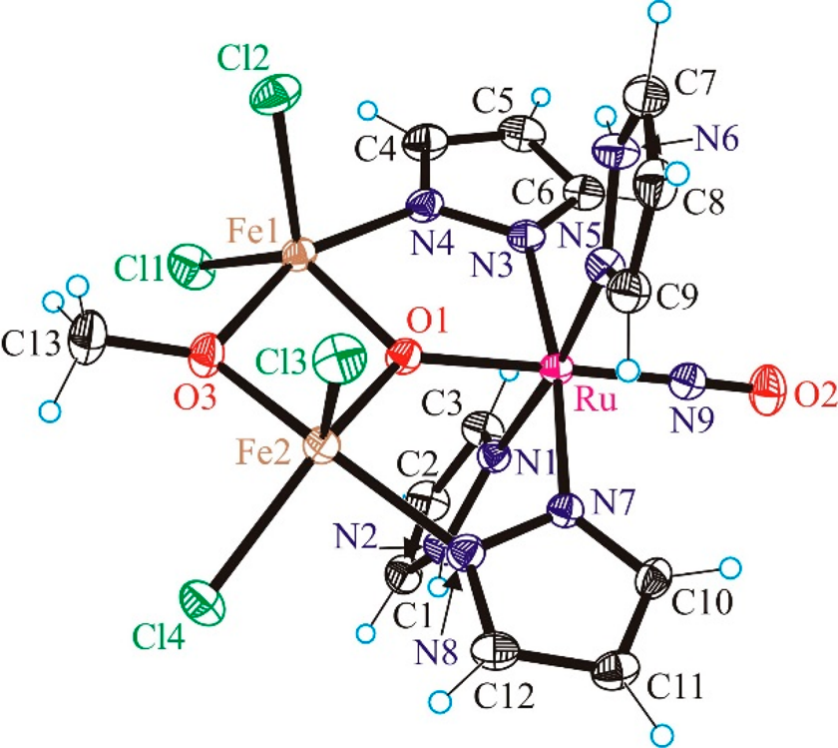
ORTEP view of [Fe_2_RuCl_4_(μ_3_-O)(μ-OMe)(μ-pz)_2_(NO)(Hpz)_2_] (**6**). Selected bond distances (Å) and bond angles (deg): Ru–O1 1.9459(19), Ru–N9 1.758(3), Ru–N1 2.076(2), Ru–N3 2.063(2), Ru–N5 2.071(2), Ru–N7 2.068(2), Fe1–O1 1.931(2), Fe1–O3 1.992(2), Fe1–N4 2.076(3), Fe1–Cl1 2.2411(9), Fe1–Cl2 2.2408(9), Fe2–O1 1.950(2), Fe2–O3 1.960(2), Fe2–N8 2.082(2), Fe2–Cl3 2.2200(9), Fe2–Cl4 2.2639(8), N9–O2 1.146(3); N9–Ru–O1 176.92(10), O2–N9–Ru 176.0(2).

**Figure 3:**
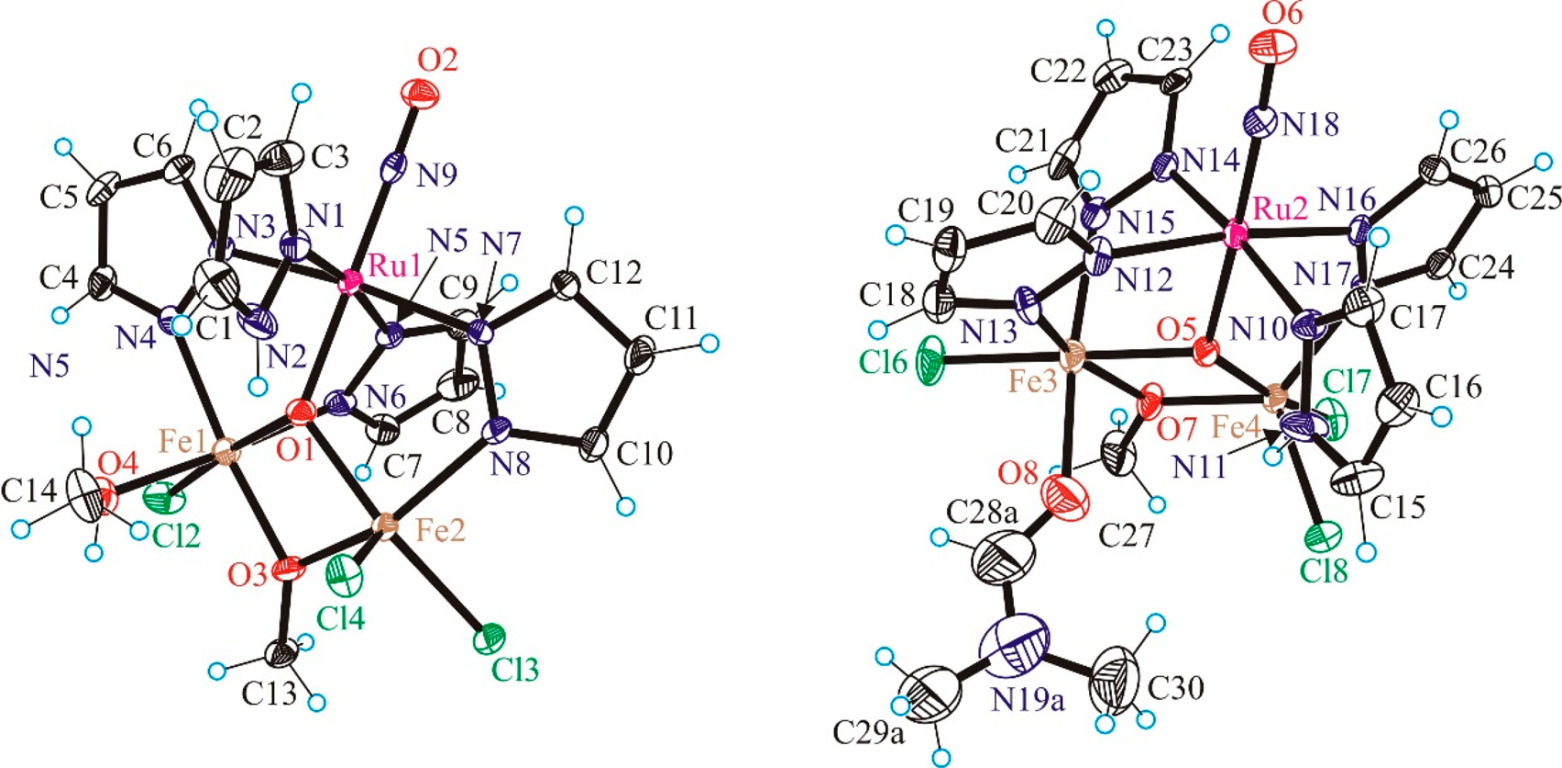
ORTEP view of crystallographically independent complexes in [Fe_2_RuCl_3_(μ_3_-O)(μ-OMe)(μ-pz)_3_(MeOH)(NO)(Hpz)][Fe_2_RuCl_3_(μ_3_-O)(μ-OMe)(μ-pz)_3_(DMF)(NO)(Hpz)]·MeOH (**7·MeOH**). Selected bond distances (Å) and bond angles (deg): Ru1–O1 1.954(5), Ru1–N9 1.762(6), Ru1–N1 2.101(6), Ru1–N3 2.063(6), Ru1–N5 2.053(6), Ru1–N7 2.059(6), Fe1–O1 2.010(5), Fe1–O3 1.989(5), Fe1–O4 2.107(5), Fe1–N4 2.062(6), Fe1–N6 2.093(6), Fe1–Cl2 2.279(2), Fe2–O1 1.986(5), Fe2–O3 1.957(5), Fe2–N8 2.051(6), Fe2–Cl3 2.278(2), Fe2–Cl4 2.258(2), N9–O2 1.141(7); N9–Ru1–O1 178.2(3), O2–N9–Ru1 178.2(6); Ru2–O5 1.934(5), Ru2–N18 1.760(6), Ru2–N10 2.086(6), Ru2–N12 2.074(6), Ru2–N14 2.063(6), Ru2–N16 2.077(6), Fe3–O5 1.996(5), Fe3–O7 2.018(5), Fe3–O8 2.078(7), Fe3–N13 2.078(7), Fe3–N15 2.106(6), Fe3–Cl6 2.275(2), Fe4–O5 1.962(5), Fe4–O7 1.935(5), Fe4–N17 2.053(6), Fe4–Cl7 2.314(2), Fe4–Cl8 2.248(2), N18–O6 1.145(8); O6–N18–Ru2 176.1(6).

**Figure 4:**
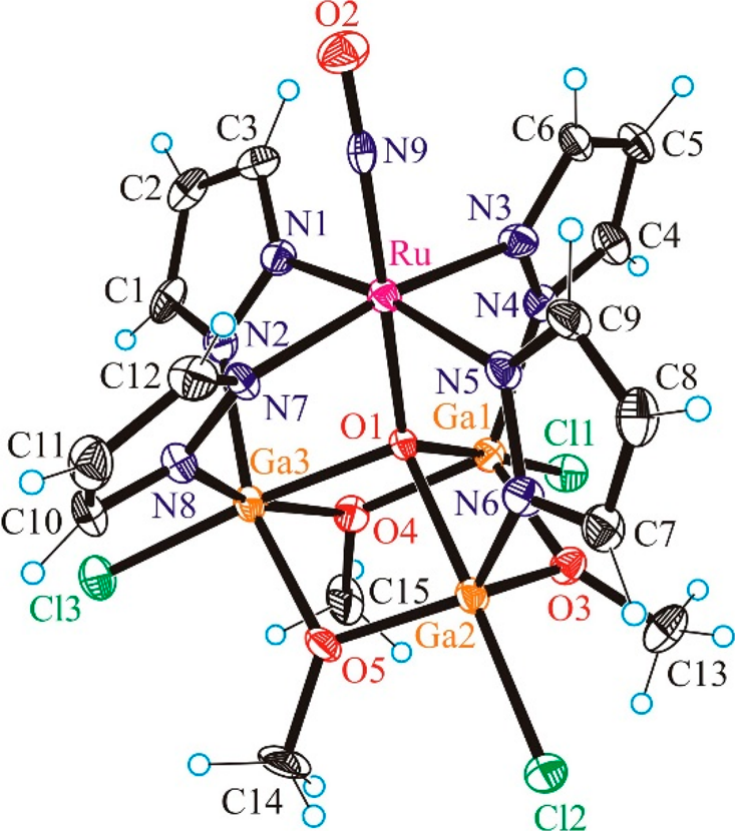
ORTEP view of [Ga_3_RuCl_3_(μ_4_-O)(μ-OMe)_3_(μ-pz)_4_(NO)] (**8**). Selected bond distances (Å) and bond angles (deg): Ru–O1 1.949(3), Ru–N9 1.752(4), Ru–N1 2.055(4), Ru–N3 2.078(4), Ru–N5 2.072(4), Ru–N7 2.062(4), Ga1–O1 2.028(3), Ga1–O3 1.907(3), Ga1–O4 1.894(4), Ga1–N4 1.964(4), Ga1–Cl1 2.2268(12), Ga2–O1 2.047(3), Ga2–O3 1.914(3), Ga2–O5 1.891(3), Ga2–N6 1.965(4), Ga2–Cl2 2.2191(13), Ga3–O1 2.028(3), Ga3–O4 2.009(3), Ga3–O5 2.003(3), Ga3–N2 2.025(4), Ga3–N8 2.039(4), Ga3–Cl3 2.2339(13), O2–N9 1.147(5); N9–Ru–O1 177.27(14), O2–N9–Ru 175.2(3).

The results of X-ray diffraction studies of complexes **3**–**8** are shown in Figure S8, Figure 1, Figure S23, and Figures 2–4, respectively. Selected bond lengths and bond angles are quoted in the legends to the figures. Complex **4** is centrosymmetric with a magnesium(II) ion lying on the center of symmetry, which joins the two octahedrally coordinated ruthenium centers. Each ruthenium atom is coordinated by one 1*H*-pyrazole (Hpz) and three pyrazolate (pz) ligands in the equatorial plane and by nitrosyl and hydroxido groups in axial positions. Two pyrazolates and the hydroxido group from one ruthenium complex and two pyrazolates and one hydroxido group from the second ruthenium complex act as bridging ligands (μ-pz and μ-OH) to Mg(II). The six-coordinate surrounding geometry of Mg(II) is approaching octahedral with four pyrazolato ligands in the equatorial plane and two hydroxido ligands in axial positions. However, the equatorial plane is significantly inclined and the angles between the axial bonds and the equatorial plane deviate by ca. ±7° from the 90° typical for *O*_*h*_ symmetry, which is not unexpected for an alkaline-earth-metal (group 2) ion.

Complex **6** (Figure 2) consists of three metal centers, namely a ruthenium and two iron atoms, in a trigonal-planar geometry surrounding a μ_3_-bridging oxygen. The three metal centers are straddled by two bridging pyrazolates, while only the iron atoms are straddled by a μ-methoxido group. The ruthenium center adopts a pseudo-octahedral coordination geometry with two pyrazolato and two pyrazole ligands in the equatorial plane and the NO ancillary ligand and μ_3_-oxido ligand in axial positions. The two Fe(III) centers are five-coordinate and, in addition, are bound by two ancillary chlorido ligands each.

Complex **7·MeOH** crystallizes in the noncentrosymmetric monoclinic space group *Cc* with two chemically different complexes, as shown in Figure 3, and one interstitial molecule of methanol in the asymmetric unit. Addition of a small amount of DMF to a methanolic solution of **6** resulted in substitution of one chlorido coligand by a molecule of MeOH or DMF. The overall charge balance was reached by deprotonation of 1*H*-pyrazole coordinated to Ru via N5 or N16 and its coordination to iron(III) (Fe1 and Fe3 in Figure 3) via N6 and N17, respectively, as a bridging ligand. Another notable feature is the increase in the coordination number of Fe1 and Fe3 from 5 to 6. As expected, the bond lengths from donor atoms to Fe1 and Fe3 are significantly increased in comparison to those around Fe2 and Fe4. Indeed, the comparable bonds around Fe1 are longer than those around Fe2 (Fe1–N4 2.062(4) Å vs Fe2–N8 2.051(4) Å; Fe1–O1 2.010(4) vs Fe2–O1 1.986(4); Fe1–O3 1.989(4) Å vs Fe2–O3 1.957(4) Å; Fe1–Cl2 2.2790(15) Å vs Fe2–Cl4 2.2581(15) Å). It should be noted that the last two bonds are not directly comparable; nevertheless, the overall trend is clear. The same picture is reproduced for the Fe3 and Fe4 environment. The M–L bonds around Fe3 are longer than those around Fe4 (see legend to Figure 3).

The heterometallic complex **8** (Figure 4) was prepared as a structural and electronic analogue of the Fe_2_Ru clusters **6** and **7·MeOH**, but with a redox-inactive (and diamagnetic) Fe(III) congener, namely gallium(III), which shares some properties with high-spin iron(III) due to its comparable ionic radius (*r*_Ga(III)_ ≈ 0.620 Å vs *r*_Fe(III)_ ≈ 0.645 Å), charge, and coordination chemistry. Gallium(III) has been often employed as a structural substitute for iron(III) in biomimetic systems.^53−57^ The redox-inactive Ga platform is also a useful comparison for the Fe_2_Ru complexes in terms of the photoreactivity of the Ru-NO moiety. Complex **8** consists of three gallium(III) atoms and one ruthenium atom that are bridged by a μ_4_-oxido bridging group stemming from the octahedral ruthenium complex. Ruthenium is coordinated by four pyrazolato ligands in the equatorial plane, with NO and the oxido group in axial positions. The three Ga(III) ions and three ancillary methoxido groups occupy the corners of a six-membered ring in an alternating manner. The Ga_3_O_3_ ring adopts a slightly distorted chair conformation, in contrast to trimeric organoalkoxochloridogallanes with strongly distorted boat conformations.^58^ Pentanuclear alkoxido gallium(III) complexes are also well-documented in the literature.^59^ Among the four pyrazolates acting as bridging ligands to gallium, two are bound to Ga3 and the remaining two are singly coordinated to Ga1 and Ga2, respectively. Ga3 is six-coordinate, while Ga1 and Ga2 are five-coordinate. Each coordination polyhedron is completed by one chlorido coligand which is in a position *trans* to the μ_4_-oxido bridge. The Ga–Cl bond distances in organoalkoxochloridogallanes (2.13–2.18 Å) are shorter than those in complex **8** (2.2191(13)–2.2339(13) Å). The Ga–O bond distances in complex **8** are either similar (1.891(3)–1.914(3) Å) or longer (2.003(3)–2.047(3) Å) in comparison to those in organoalkoxochloridogallanes (1.90–1.91 Å). Lengthening of the respective bond lengths in **8** was expected due to the increase in coordination number from 4 to 5 (Ga1 and Ga2) and 6 (Ga3), as seen for other organoalkoxochloridogallanes.^58^

The Ru–N–O entity is very close to linear in all heteronuclear complexes studied, varying very slightly from 175.2(3)° in **8** to 178.2(2)° in **7·MeOH**. The interatomic N–O distances (1.150(2) Å in **4**, 1.146(3) Å in **6**, 1.141(5) and 1.145(6) Å in **7**, and 1.147(5) Å in **8**) are slightly shorter than in free NO (1.154 Å)^60^ but significantly longer than in free NO^+^ (1.063 Å).^61^ These geometric parameters along with ν_NO_ stretching vibrations as a spectroscopic handle indicate that the Ru(NO) moiety can be best described in Enemark and Feltham notation^62^ as {Ru(NO)}^6^, where 6 is the number of electrons in the d (ruthenium) and π*(NO) orbitals. Hence, this moiety is in accordance with Ru^II^ (4d^6^, *S*_Ru_ = 0) bonded to NO^+^ (π*^0^, *S*_NO_ = 0).^63−68^

### Magnetometry

The results of temperature-dependent magnetic susceptibility measurements on the polycrystalline heterotrinuclear clusters **6** and **7·MeOH** are shown in Figure S24. At room temperature the χ_M_*T* products for **6** and **7·MeOH** are 4.414 and 6.037 cm^3^ K mol^–1^, respectively. These values are lower than the 8.754 cm^3^ K mol^–1^ expected for two uncoupled Fe^III^ ions (*S* = 5/2, *g* = 2). When the temperature was lowered, the χ_M_*T* product for **6** decreased almost linearly to reach 0.077 cm^3^ K mol^–1^ at 2 K, while in the case of **7·MeOH** it decreased nonlinearly from 300 to 100 K and then almost linearly from 100 K to reach 0.08 cm^3^ K mol^–1^ at 15 K. The overall shape of the curves and χ_M_*T* values close to zero at low temperature indicate antiferromagnetic interactions in **6** and **7·MeOH** and an *S* = 0 ground state for both complexes. When the crystallographic data are taken into account, the most likely scenario is an antiferromagnetic interaction between two Fe^III^ ions, with the Ru(NO) unit being diamagnetic (*S* = 0). In this context the magnetic susceptibility data of the two compounds were analyzed by using an isotropic exchange spin Hamiltonian (see eq 1 in the Experimental Section) with each site being high-spin Fe^III^.^69^ Fits of experimental data using this model yielded the following *J*_Fe–Fe_ and *g*_Fe_ parameters: −49.4(4) cm^–1^ and 2.158(2) for **6** and −25.64(2) cm^–1^ and 2.076(1) for **7·MeOH**, respectively.

### ^57^Fe Mössbauer Spectroscopy

**Figure 5:**
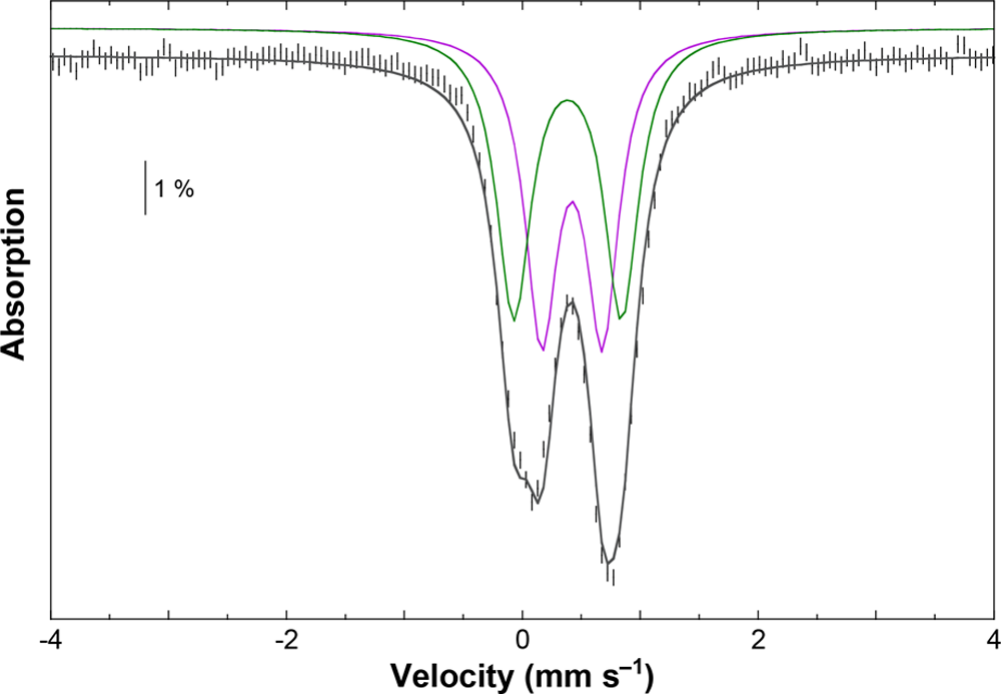
Experimental 82 K Mössbauer spectrum (hatched bars) recorded on a powder sample of **7·MeOH** using a 0.06 T external magnetic field applied parallel to the γ-beam. A simulation is overlaid on the experimental data as a gray solid line. The contributions of site 1 (δ = 0.39(2) mm s^–1^, Δ*E*_Q_ = 0.91(5) mm s^–1^) and site 2 (δ = 0.42(2) mm s^–1^, Δ*E*_Q_ = 0.52(5) mm s^–1^), in a 1:1 ratio, are displayed above the spectrum as green and purple lines, respectively.

The 82 K Mössbauer spectra of Fe-containing complexes **6** and **7·MeOH** are reproduced in Figure S25 and in Figure 5, respectively. A slightly asymmetric doublet was detected for **6**, suggesting that the two iron sites are similar. Whatever the analysis, the isomer shift value for each iron site (0.38(2) mm s^–1^, see legend to Figure S25 in the Supporting Information) is consistent with a ferric ion in a high-spin or an intermediate-spin configuration.^70−73^ However, the quadrupole splitting typically observed for an *S*_Fe_ = 3/2 spin state is significantly larger than what is seen here (0.71–0.83 mm s^–1^, see legend to Figure S25 in the Supporting Information).^73,74^ Accordingly, a high-spin *S*_Fe_ = 5/2 state is proposed for each of the two iron sites in **6**, consistent with the magnetic susceptibility data. The spectrum recorded at 6 K using a strong external magnetic field evidenced an *S* = 0 ground state (see Figure S26 in the Supporting Information), in agreement with the temperature dependence of the χ_M_*T* vs *T* curve (see Figure S24).

With regard to **7·MeOH**, the low-velocity line (Figure 5) clearly evidenced different iron sites. The 82 K Mössbauer spectrum was reproduced by assuming two different doublets (see caption of Figure 5). Parameters of site 1 in **7·MeOH** are similar to those observed for the ferric ions in **6**, suggesting that this site corresponds to the five-coordinate ferric ions Fe2 and Fe4 (see Figure 3). The isomer shift of site 2 is slightly larger than that of site 1 (0.42(2) vs 0.39(2) mm s^–1^), suggesting an increase in the coordination number, while the quadrupole splitting is significantly smaller (0.52(5) vs 0.91(5) mm s^–1^), indicating a more spherical electronic distribution. These observations taken together strongly suggest that site 2 corresponds to the six-coordinate ferric ions Fe1 and Fe3 (see Figure 3) with a local high-spin configuration.

### Photoinduced NO Linkage Isomerization and NO Release

The introduction of the NO ligand provides a convenient spectroscopic handle. Compounds **4** and **6**–**8** were investigated for photoinduced linkage isomerization (PLI) at low temperatures by using infrared spectroscopy, which is a sensitive tool to detect even small amounts of nitrosyl linkage isomers via the shift of the characteristic ν(NO) stretching vibration.^75,76^ The wavelength dependences for photogenerating MS1 (M-ON) or MS2 (M-η^2^-NO) are different, clearly distinguishing these two states. For MS2 irradiation at lower wavelengths is required, in the blue to UV spectral range, while for MS1 irradiation in the blue to green spectral range induced a maximum population of this isomer. The IR data of irradiated samples (summarized in Table 1 showed that in **4** and **6** the signatures of two PLIs are detected, while in **8** only one PLI is found, but in **7·MeOH** no PLI is detected (the population of these species is ≪1%). In **8**, irradiation with blue light (maximum at 476 nm) leads to the appearance of a weak, new band at 1725/1710 cm^–1^, while the ground-state (GS) band at 1866 cm^–1^ decreases slightly, indicating a population of about 0.5–1.0%. The shift of the ν(NO) band of about 140 cm^–1^ to lower energy is typical for MS1, the isonitrosyl Ru–ON linkage isomer.^75−77^ The effect is reversible, either by irradiating at other wavelengths or by warming back to room temperature.

**Table 1:** Vibrational Frequencies (in cm^–1^) Measured at 10 K, using KBr Pellets^a^

compound	GS	MS1	MS2
**4**	1869/1854	1723/1710/1704	1514/1496
**6**	1897/1867 (sh)	1741	1579/1574
**7·MeOH**	1866	na	na
**8**	1866	1725/1710	na

a GS indicates the ν(NO) stretching frequency in the ground state: i.e., before light irradiation. MS1 denotes the ν(NO) stretching frequency in the first linkage isomer, thought to be an iso-nitrosyl configuration. MS2 denotes the ν(NO) stretching frequency in the second linkage isomer, thought to be a side-on configuration of the nitrosyl.

**Figure 6:**
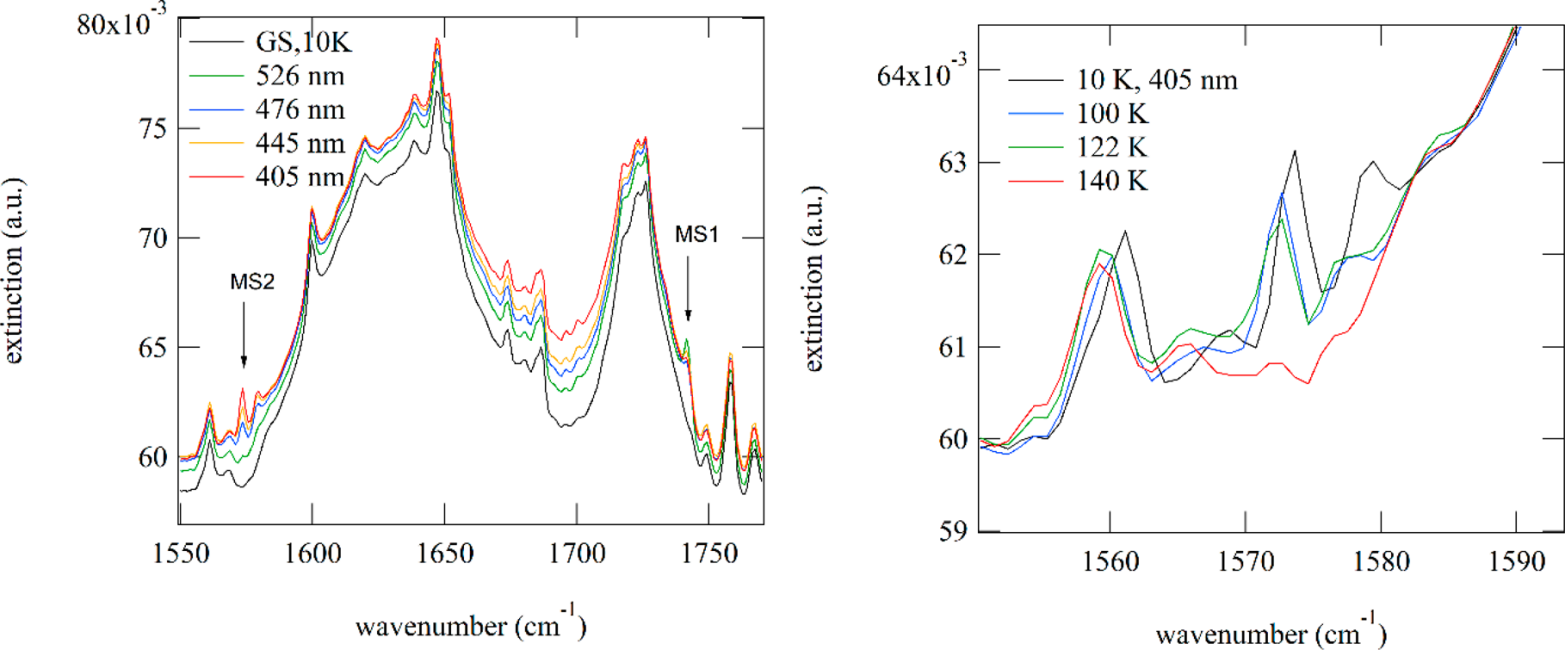
Generation of PLI in **6**. (left) Wavelength dependence of the population. MS1 can be generated in the blue to green spectral range, while MS2 is generated by irradiation in the blue to ultraviolet spectral range. (right) When the sample is heated, the PLI relaxes back to GS, as illustrated by the disappearance of the absorption band of MS2, which decays at around 130 K in **6**.

**Figure 7:**
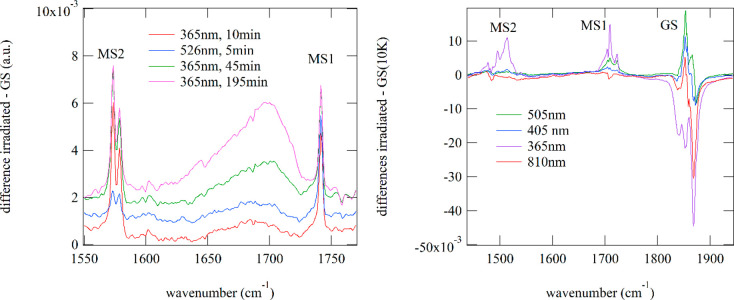
Reversibility of PLI. (left) In **6** MS2, characterized by its absorption bands at 1579/1574 cm^–1^, is generated with UV light (365 nm) and can be partially erased by green light (526 nm). Long-term irradiation with UV light (365 nm) leads to additional effects, indicated by the appearance of a broad band at around 1700 cm^–1^. (right) In **4** MS2, characterized by its bands at 1514/1496 cm^–1^, is generated by 365 nm light and can be completely erased by 505 nm light. Using infrared light (810 nm), MS2 and MS1 can be erased completely.

Similarly, for compounds **4** and **6** we also observe the generation of new bands at 1741 cm^–1^ (**6**) and 1723/1710/1704 cm^–1^ (**4**) on irradiation with blue-green light corresponding to the ν(NO) bands of MS1. The corresponding ν(NO) bands of GS at 1897/1867(sh) cm^–1^ (**6**) and 1869/1854 cm^–1^ (**4**) decrease, indicating rather low populations in the range of 1–2%. The maximum population of these MS1 states is found on irradiation with 505 nm light for **4** and 526 nm for **6** (see Figures 6 and 7). In addition, we found for both compounds bands at 1579/1574 cm^–1^ (**6**) and 1514/1496 cm^–1^ (**4**). These bands are assigned to the MS2 side-on (Ru-(η_2_-(NO)) state, as documented for other compounds, where typical shifts of 300 cm^–1^ to lower wavenumbers with respect to the GS were observed.^44^ The wavelength dependences for generating MS1 or MS2 are different, clearly distinguishing these two states. For MS2 irradiation at lower wavelengths is required, in the blue to UV spectral range. With green light, e.g., 526 nm for **6**, we can partially erase MS2 (see Figure 7). Full erasure of MS2 and MS1 is possible with red-infrared light or by heating above about 130 K. While in **6** MS2 can be generated with blue-green light (Figure 6), in **4** we need UV light (365 nm). However, irradiation with UV light might induce other effects. Indeed, we observed that prolonged irradiation with UV light leads to irreversible modifications. As can be seen in Figure 7, a broad band around 1700 cm^–1^ appears and, in addition, a well-defined band at 2225 cm^–1^. The latter is known from other studies^78,79^ and is assigned to NO release and possible reactions in KBr to yield free N_2_O, even at very low temperatures (10 K).^49^

**Figure 8:**
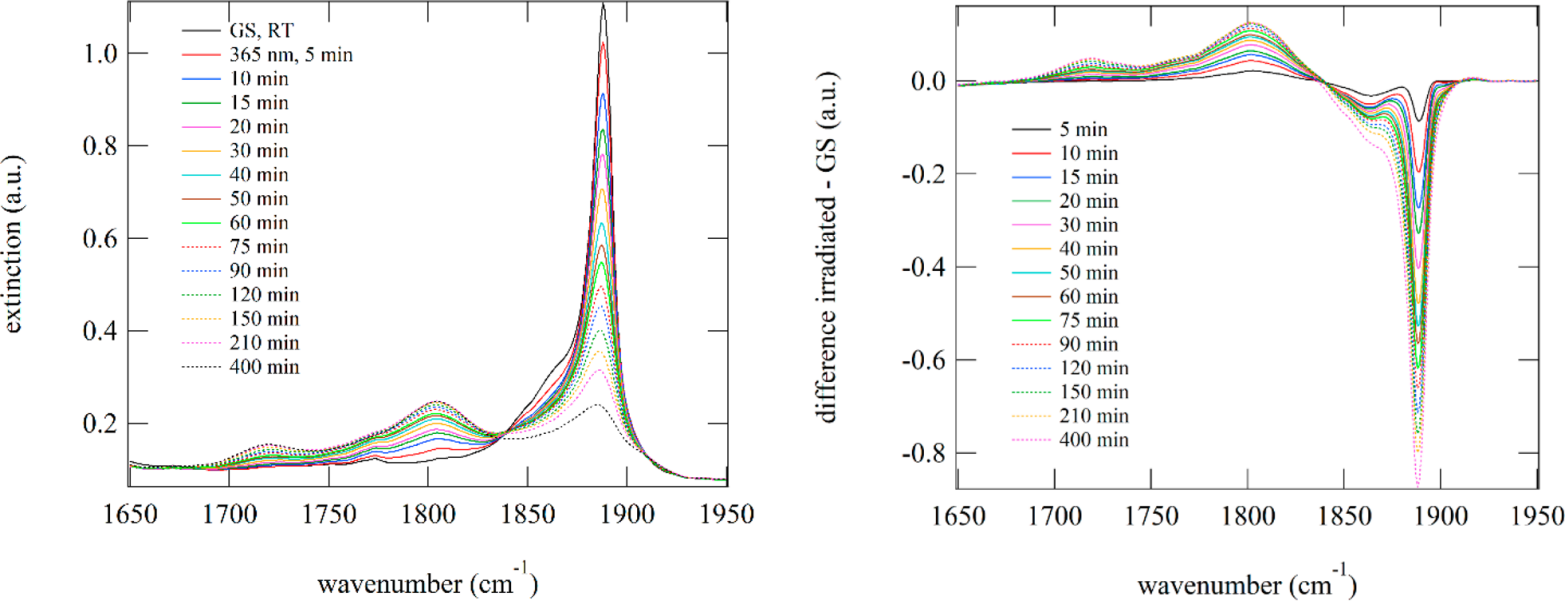
IR spectra at room temperature of **6** upon irradiation with 365 nm. On the right, the corresponding difference spectra with respect to the initial state are plotted.

**Figure 9:**
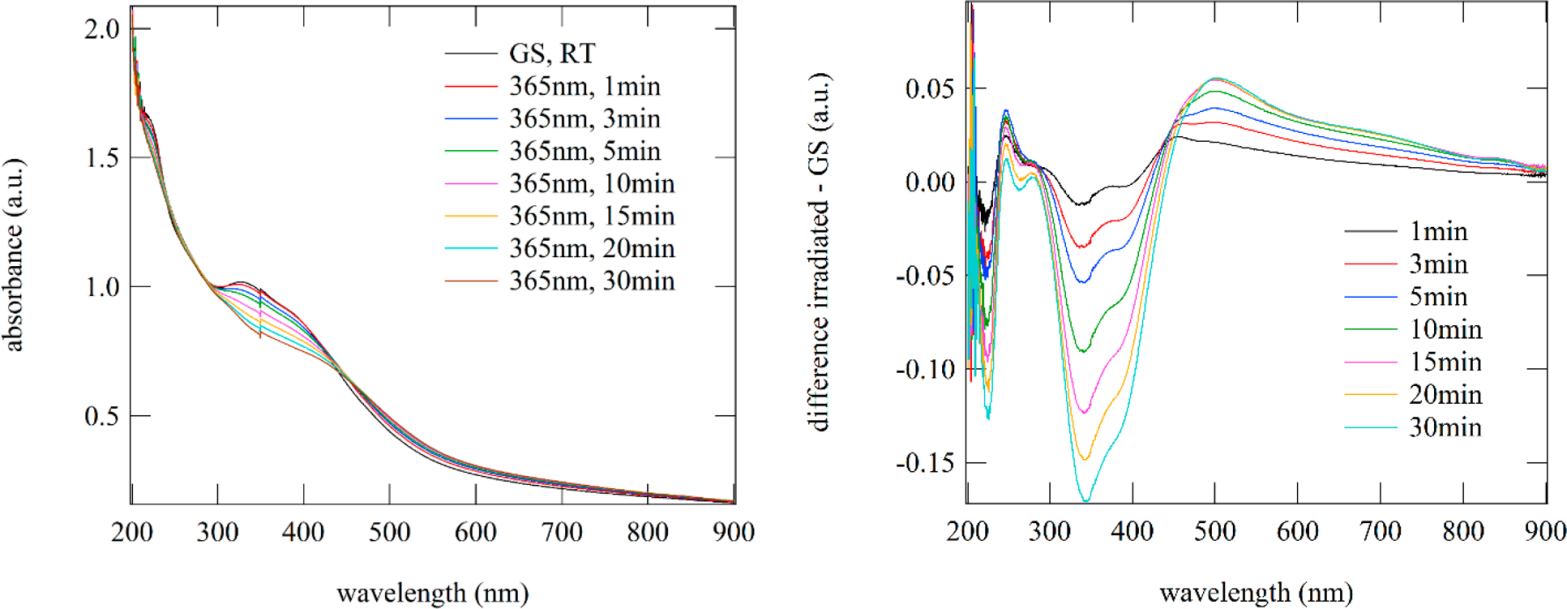
UV–vis spectra of **6** upon irradiation with 365 nm light at room temperature. On the right, the corresponding difference spectra with respect to the initial state are plotted, illustrating the decrease in the absorption bands in the UV spectral range and the increase in bands in the green to red spectral range.

Given that even at 10 K we can observe the signature of NO release in the solid state, we studied the effect of irradiation on **6** in more detail at room temperature (compound **4** exhibits the same behavior to some extent). For this purpose, we prepared a KBr pellet and measured the evolution of the IR and UV–vis spectra as a function of light irradiation dose (Figures 8 and 9). Irradiation with 365 nm (405 nm leads to the same results) leads to irreversible modification of the spectra. Notably, the ν(NO) band at 1888 cm^–1^ decreases strongly and almost vanishes after 7 h of irradiation with 100 mW/cm^2^ at 365 nm. New bands arise at 1803 and 1718 cm^–1^. In fact, the entire IR spectrum undergoes dramatic changes (see Figure S27 in the Supporting Information), including the appearance of a band at 2221 cm^–1^, indicating a photoinduced NO release, probably followed by further reactions/decomposition. This is corroborated by the UV–vis absorption spectra as a function of irradiation, where the GS bands at 390 and 335 nm decrease and new bands in the range 500–700 nm arise. Such absorption changes are well-known from photoinduced NO release in solution.^49,80^

### DFT and *Ab Initio* Results

#### Energetics and *J* Coupling

According to the B3LYP/def2-SVP calculations, we found the broken symmetry (BS) singlet state, ^1^[**6**]^0^ (the first index stands for spin multiplicity, *m*_*S*_ = 2*S* + 1), to be energetically preferred in the case of the neutral complex, but the high-spin state (^11^[**6**]^0^; from *S*_tot_ = 5/2 + 5/2 = 5) lies very close in energy (Table S1). The absolute energy difference is 5 kJ/mol, as shown in Table 2, and the *J* coupling is −0.201 kJ/mol (−16.8 cm^–1^), note that *J* coupling is given with respect to eqs 1–3 and the negative sign is in line with the antiparallel (antiferromagnetic) spin/spin coupling between the two irons determined experimentally in [**6**]^0^. Still, the B3LYP determined *J* coupling is underestimated by almost a factor of 3 when compared to the experimentally determined value (−49.4 cm^–1^). The ^13^[**6**]^0^ state, which accounts for an open shell configuration also at the [Ru-NO] moiety (from *S*_tot_ = *S*_Fe1_ + *S*_Fe2_ + *S*_NO_ = 5/2 + 5/2 + 1 = 6), is not energetically favored, and will not be considered further. The BLYP/def2-SVP calculations are qualitatively in agreement with the B3LYP approach. The BLYP-calculated *J* coupling of [**6**]^0^ is −39.0 cm^–1^, which is almost twice as large as that for the B3LYP functional and hence closer to the experimental value (−49.4 cm^–1^). The CASSCF and the NEVPT2 calculations are in accordance with the DFT results: i.e., the spin state preference is confirmed. Nevertheless, CASSCF tends to yield lower LS vs HS difference energies in comparison to B3LYP. NEVPT2 finds the LS to HS energy gap in close agreement with B3LYP for the oxidized and reduced forms of **6**, while for the neutral complex the NEVPT2 energy gap is still smaller by a factor of 2 when it is compared against B3LYP. CASSCF- and NEVPT2-calculated *J* couplings of the neutral species of **6** (extracted for *S* = 5, making Δ*E* relative to *S* = 0) are −0.067 and −0.159 kJ/mol (−5.6 and −13.3 cm^–1^), respectively. The underestimation of *J* coupling for the CASSCF and NEVPT2 methods confirms the trends in results previously found for a dicopper(II)-tetrakis(μ-acetato)-diaqua complex.^81^ Results for the oxidized and reduced species of **6** can be found in the Supporting Information (section on DFT and *ab initio* results of [**6**]^+^ and [**6**]^−^ and Tables S1 and S2).

**Table 2:** Relative B3LYP and BLYP def2-SVP Energies (Δ*E* = *E*_S_), ⟨*S*^*2*^⟩ Expectation Values, *J* Coupling of Spin States, and Relative CASSCF and NEVPT2 def2-SVP Energies of the Studied Species of [**6**]^0^ a

	Δ*E* (kJ/mol)		*S*^2^ (au)		*J* (kJ/mol)		Δ*E* (kJ/mol)	
	B3LYP	BLYP	B3LYP	BLYP	B3LYP	BLYP	CASSCF	NEVPT2
^13^[**6**]^0^	101.1	119.4	42.029	42.017	b	b		
^11^[**6**]^0^	0.0	0.0	30.023	30.017			0.0	0.0
^9^[**6**]^0^	37.0	1.5	20.053	20.039	3.707	0.147	–0.3	–0.7
^7^[**6**]^0^	96.7	32.0	12.083	12.062	5.391	1.781	–0.6	–1.4
^5^[**6**]^0^	155.0	73.0	6.808	6.677	6.677	3.130	–0.8	–1.9
^3^[**6**]^0^	217.1	67.7	3.596	3.663	8.217	2.570	–0.9	–2.2
^1^[**6**]^0^	–5.0	–11.7	4.956	4.846	–0.201	–0.466	–1.0	–2.4

a Note that *J* coupling is given with respect to eqs. 2 and 3.

b Not relevant.

The optimized B3LYP/def2-SVP bond distances and angles of the chosen HS and LS geometries of **6** are presented in Table S3. In the case of the neutral species, the high-spin state *m*_*S*_ = 11 and the energetically preferred BS singlet spin states have been taken into account. While the Ru-NO moiety is linear in **6**, in the case of a reduced species the Ru–N9–O2 angle becomes bent^82−85,26^ (see section on optimized geometries and Table S3 in the Supporting Information).

#### Electronic Structure Characterization, Frontier Orbitals, and TD-DFT

**Figure 10:**
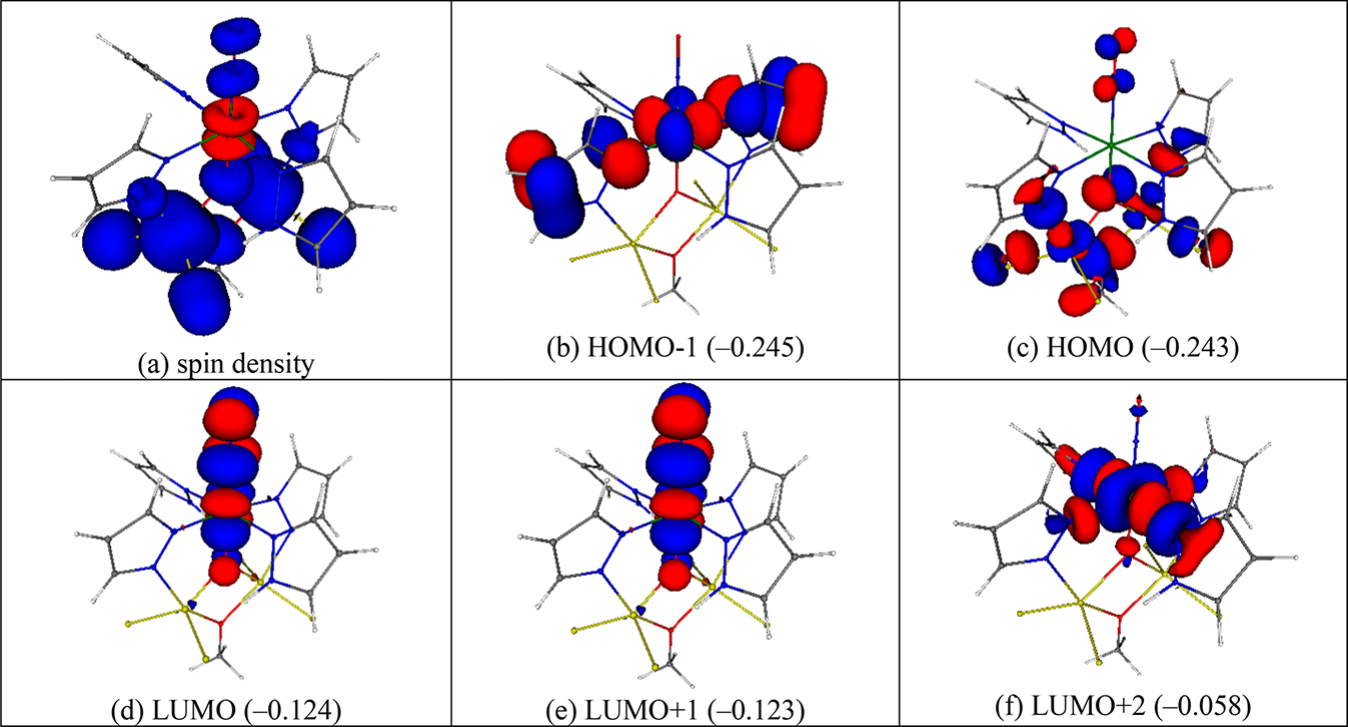
Spin density and frontier α orbitals of ^11^[**6**]^0^, ^10^[**6**]^+^, and ^10^[**6**]^−^ (eigenvalues in hartrees are shown in parentheses). The isosurface values are 0.002 (spin density) and 0.04 (frontier orbitals) e bohr^–3^. Note that the pairs HOMO/HOMO-1 and LUMO/LUMO+1 are each nearly degenerate (energy separations are 440 and 220 cm^–1^, respectively).

In the neutral ^11^[**6**]^0^ and BS ^1^[**6**]^0^ species, we can identify 5α d orbitals (or 5β orbitals in BS ^1^[**6**]^0^), which supports a formal d^5^ electron configuration on both iron atoms (see Table S2). The spin population suggests a physical d^4^ electron configuration, with one unpaired electron from each iron center being delocalized over the complex, while the atomic orbital (AO) α d populations confirm the d^5^ configuration on both iron centers of ^11^[**6**]^0^ (see Figure 10a and Tables S4a and S5). In the case of BS ^1^[**6**]^0^, the spin of the Fe1 center is flipped and the d^5^ configuration is found in the β d populations (see Tables S4a and S5).

A further description of the oxidation/reduction of [**6**] with respect to electronic structure of different spin states is given in Tables S3, S4a, S4b, and S5 and Figure S28 in the Supporting Information.

The interpretation of the electron configuration given above is also in agreement with the frontier orbitals. B3LYP/def2-SVP frontier α HOMO-1, HOMO, LUMO, LUMO+1, and LUMO+2 orbitals for complexes ^11^[**6**]^0^, ^10^[**6**]^+^, and ^10^[**6**]^−^ complex are depicted in Figure 10. The HOMO of ^11^[**6**]^0^ is delocalized around the Fe atoms (10% of the HOMO is localized on each iron), and HOMO-1 is on the pz-Ru-pz unit with two pyrazolato (pz^–^) ligands in positions *trans* to each other. The first two virtual orbitals of ^11^[**6**]^0^, LUMO and LUMO+1, have a Ru-NO antibonding nature. The pairs HOMO/HOMO-1 and LUMO/LUMO+1 are each nearly degenerate (energy separations are 440 and 220 cm^–1^, respectively). The B3LYP/def2-TZVP d populations are quantitatively in agreement (see Table S6) with the B3LYP/def2-SVP populations (see Table S5).

**Figure 11:**
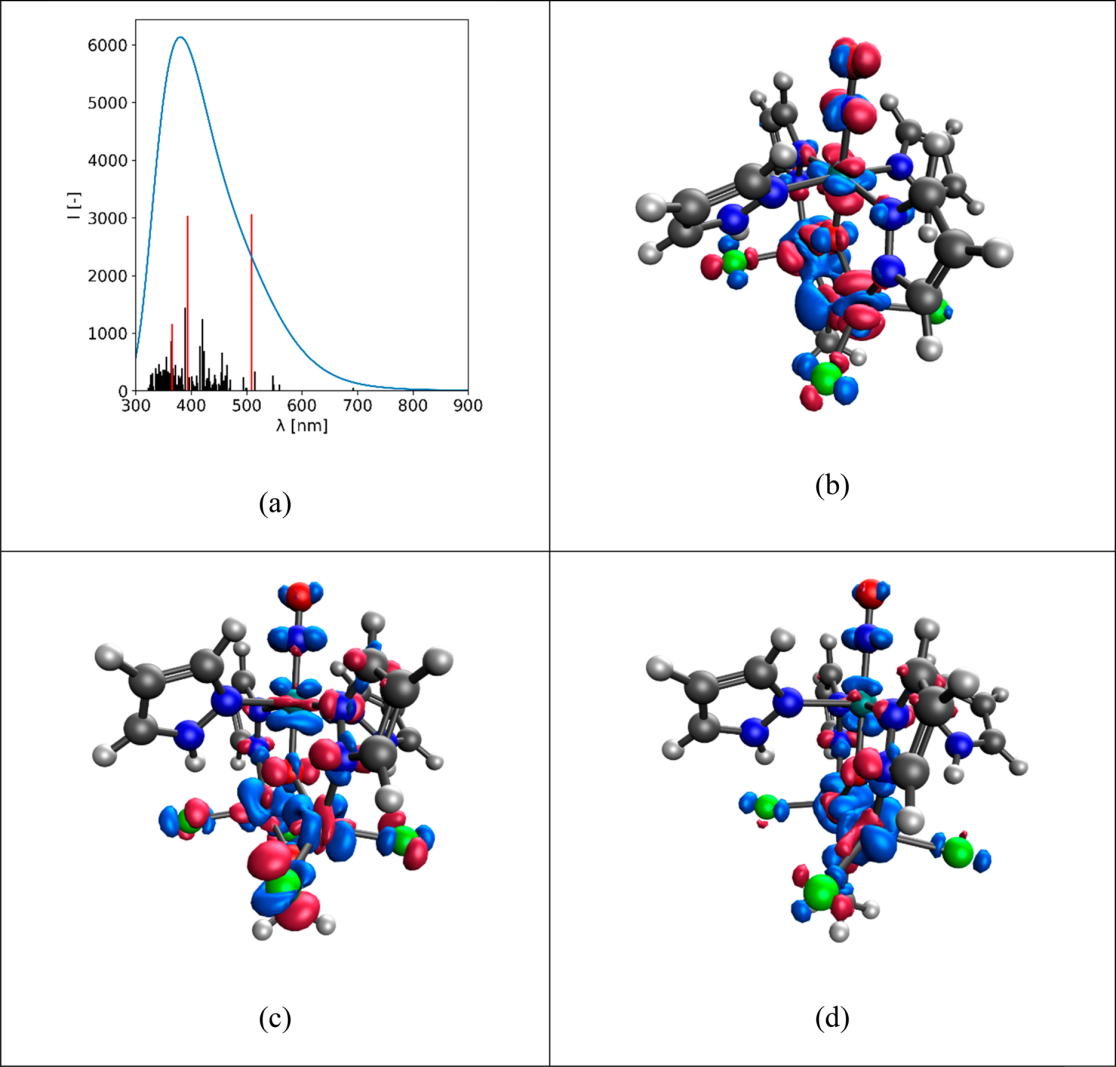
(a) TD-DFT calculated spectrum with chosen transitions shown as red bars. TD-DFT computed changes in excited-state electron density with respect to the ground state electron density at the wavelengths indicated: (b) 394 nm; (c) 389 nm; (d) 365 nm.

To probe the role of the nitrosyl ligand in the electronic absorption spectra of **6**, TD-DFT calculations were performed with the calculated spectrum shown in Figure 11. Also included are transition densities of TD excited-state electron density with respect to the ground-state electron density for several states. It is evident that the change in UV–vis intensity in Figure 9 upon NO release (or, minimally, a change of coordination in the {Ru(NO)} moiety) is related to the non-negligible contribution of the NO group in these UV–vis transitions, most notably for the visible band at 509 nm.

#### Mössbauer Parameters

The TZVP basis set is used for calculation of Mössbauer parameters for comparison with the present experiments and EPR parameters for potential future studies (see the Supporting Information). These B3LYP/TZVP calculated results for iron centers of different neutral species of **6** are compiled in Table 3 (charged species are compiled in Table S7). We find a reasonable agreement with experiment for the neutral ^11^[**6**]^0^ species; Δ*E*_Q_ values are overestimated for the BS ^1^[**6**]^0^ species. This computational protocol involves artificial contributions in the evaluation of Δ*E*_Q_ for the individual high-spin irons in the antiferromagnetic regime. Mössbauer parameters calculated for the reduced and oxidized species of **6** are provided in Table S7. EPR (i.e., spin Hamiltonian: *g* values and ZFS) parameters for these charged species of **6** are provided in Table S8 for completeness and possible future reference.

**Table 3:** B3LYP/TZVP Mössbauer Parameters for Fe1 and Fe2 Centers of Different Neutral Species of **6**

		δ (mm s^–1^)	Δ*E*_Q_ (mm s^–1^)
^11^[**6**]^0^	Fe1	0.31	0.700
	Fe2	0.32	0.732
^1^[**6**]^0^	Fe1	0.32	1.349
	Fe2	0.34	1.456

#### Electronic Structure of **6** by X-ray Charge Density Analysis

**Figure 12:**
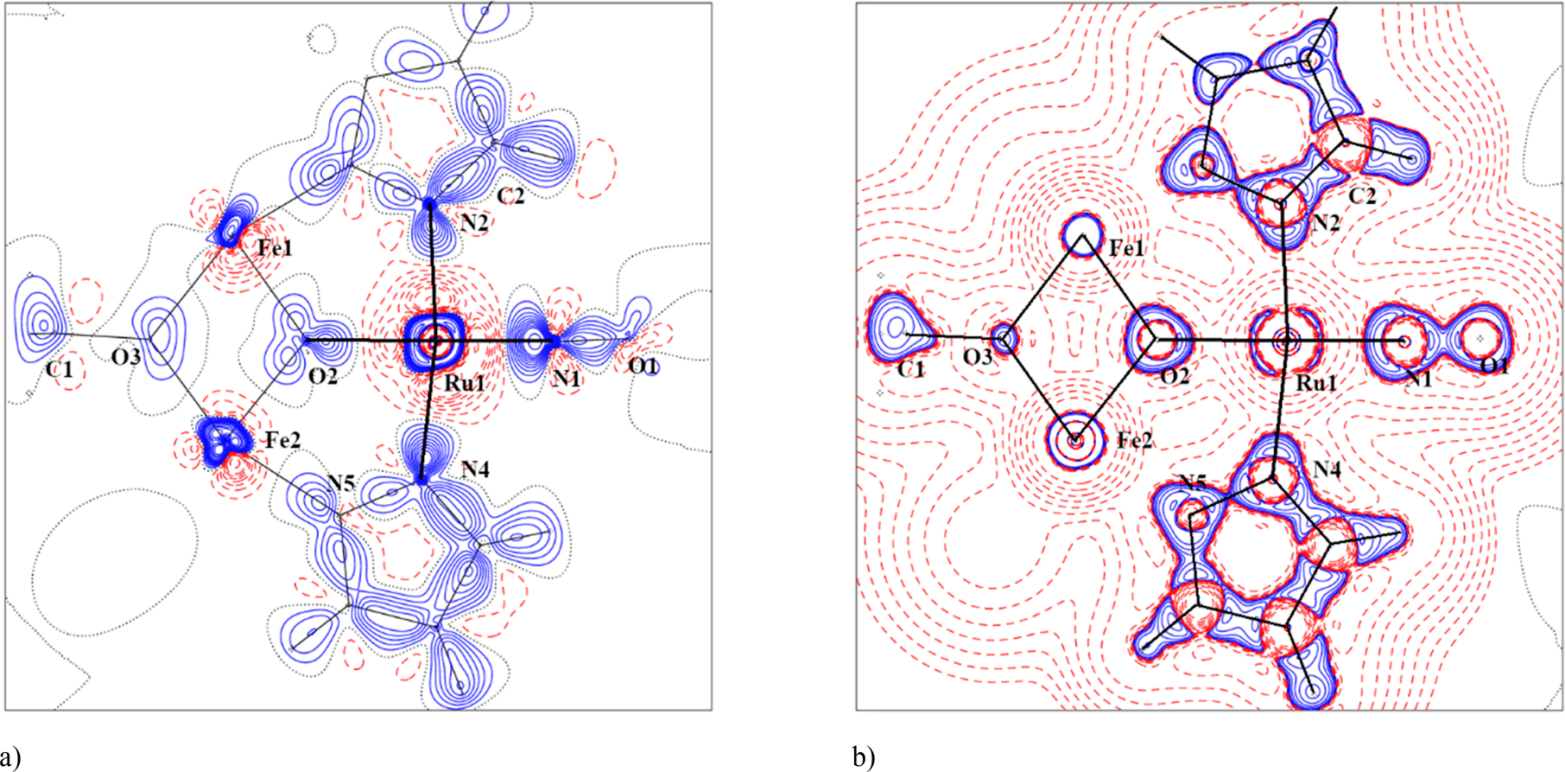
(a) Experimental static electron deformation density of **6** in the plane defined by the atoms Ru1–N1–N2. The contour spacing is 0.1 e/Å^3^, with positive contours drawn with a solid blue line and negative contours with a dashed red line. (b) Experimental distribution of the Laplacian in the plane defined by the atoms Ru1–N1–N2 of **6**. The contours are drawn at −1.0 × 10^–3^, ±2.0 × 10^*n*^, ±4.0 × 10^*n*^, ±8.0 × 10^*n*^ (*n* = −3, – 2, −1, 0, +1, +2, +3) e/Å^5^, with positive contours being drawn with a solid blue line and negative contours with a dashed red line.

Crystallographic data and details of data collection for **6**, along with selected interatomic bond lengths and bond angles, are given in Tables S9 and S10, respectively. The result of the X-ray diffraction study is shown in Figure S29. The data show that the nitrosyl quantum theory of atoms in molecules (QTAIM) charge *q* for NO^*q*^ has a value of −0.30 (N1 +0.04, O1 −0.34), which compares well with the *q* value of −0.34 (N −0.02, O −0.32) found in Na_2_[Fe(CN)_5_NO]^86^ (DFT: *q* = −0.219, N1 +0.232, O1 −0.451). According to Lee,^87^ a negatively charged nitrosyl should have two lone electron pairs (two valence shell charge concentrations, VSCCs) on the oxygen atom (see Figure S30). Asymmetry in the VSCCs is due to nonbonding interactions of chlorido coligands from neighboring molecules (see deformation density and Laplacian in Figure 12 and Figure S31). In addition, Figure 12 shows a plot of the coordination environment of the ruthenium atom.

From the QTAIM analysis (see Table S11), we can say that the strongest coordination bond is between the nitrosyl group and the ruthenium atom. The N–O bond (see Table S11) is a strong covalent bond with an ellipticity of 0.12, which excludes the pure triple bond of NO^+^ (see also Figure S32 in the Supporting Information). The coordination bonds in the ruthenium polyhedron are stronger than the bonds of both central iron atoms with coordinated atoms. Distribution of d electrons for the ruthenium atom fits with the nonbonding d_*xy*_ orbital (see Table S12). All four chlorido coligands are significantly inequivalent. The experimental charge in the atomic basin is in the range of −0.49 to −0.71, and the volume is in the interval 32.71–34.36 Å^3^ (see Table S13). From the experimental population of the d orbitals (see Table S12), the oxidation state of ruthenium atom is close to +2 and that of both iron atoms close to +0.5. On the other hand, in comparison against DFT results, it can be seen that certain d orbital populations of Fe are overestimated (see Table S12).

It is common in experimental studies (as well as in theoretical calculations) of electronic structure^88^ that the charge of the central atom is always lower than the formal oxidation state. When these charges are considered, the ruthenium atom is close to +2 and both iron atoms close to +1.5. The more positive charge on the Ru atom in comparison to those on the Fe atoms is consistent with the electron density in the bond critical point (BCP) for bonds to Ru, which are higher than those for both central Fe atoms (see Table S11). The weakest bond in the iron coordination polyhedra are Fe1–Cl2 and Fe2–Cl4 (0.436(6) and 0.490(5), respectively) (see Table S11). Furthermore, the electron density distribution of chlorido coligands is different with respect to the bond to the iron atom: i.e., their Laplacian distribution at the chosen isovalue is not yet a closed surface (see Figures S33 and S34). The observed decomposition of the complex in solution could have its origin in breaking these Fe–Cl bonds.

## Conclusions

The use of ESI MS as a powerful synthesis-targeting technique has been justified. The synthesis of the heteronuclear complexes {[Ru(μ-OH)(μ-pz)_2_(pz)(NO)(Hpz)]_2_Mg} (**4**), [Fe_2_RuCl_4_(μ_3_-O)(μ-OMe)(μ-pz)_2_(NO)(Hpz)_2_] (**6**), and [Fe_2_RuCl_3_(μ_3_-O)(μ-OMe)(μ-pz)_3_(MeOH)(NO)(Hpz)][Fe_2_RuCl_3_(μ_3_-O)(μ-OMe)(μ-pz)_3_(DMF)(NO)(Hpz)] (**7**·MeOH·2H_2_O) and the heterotetranuclear μ_4_-oxido complex [Ga_3_RuCl_3_(μ_4_-O)(μ-OMe)_3_(μ-pz)_4_(NO)] (**8**) proved to be consistent with ESI MS screening assays, which showed a high reactivity of [Ru^II^(OH)(NO)(Hpz)_4_]Cl_2_ (**5**) as a metalloligand toward oxophilic metal ions (i.e. Mg^2+^, Fe^3+^, Ga^3+^). The results obtained are in accordance with the tendency of **5** to form trinuclear species via μ-hydroxido and μ_3_-oxido bridging of the oxophilic metal centers. Interestingly, treatment of **5** with excess GaCl_3_ afforded **8**, a complex of even higher nuclearity. Attempts to prepare a similar RuFe_3_ counterpart failed, even though the mass spectra of reaction mixtures showed peaks that could be assigned to a triferric core analogous to the trigallium(III) core of **8**. Complex **8** might be of use as a starting material in attempts to introduce other paramagnetic ions, e.g. Fe^3+^, via transmetalation reactions.

X-ray crystallography revealed that in **6** the two iron sites are five-coordinate, while in **7**, which contains two chemically distinct complexes, one iron site in both complexes (Fe1 and Fe3, respectively) is six-coordinate while the second (Fe2 and Fe4, respectively), as in **6**, is five-coordinate. The established pentacoordination is a feature of note not previously observed in μ_3_-oxido-bridged trinuclear metal carboxylates. The temperature-dependent magnetic susceptibility measurements of both **6** and **7** indicate antiferromagnetic interactions between ferric ions (*S* = 5/2) with *J*_Fe–Fe_ values of −49.4(4) and −25.60(4) cm^–1^, respectively, and a resulting total *S* = 0 ground state. The diamagnetic ground state of **6** was confirmed by applied-field Mössbauer spectroscopy. Theoretical investigations of **6** confirm an antiparallel spin–spin coupling, albeit with *J*_Fe–Fe_ being underestimated, but the Mössbauer spectral parameters are well reproduced when the Fe^III^ centers are considered as independent sites with *S* = 5/2.

A key feature of the preparation of clusters containing the ruthenium-nitrosyl moiety is whether such a complex can release NO photolytically. As monitored by IR spectroscopy at 10 K, photoinduced isomerization with the generation of nitrosyl linkage isomers MS1 and MS2 was found for **4** and **6**, while the formation of only the isonitrosyl linkage isomer (MS1) was found for **8**, although the population of these metastable states did not exceed 2% for any of these. In **7**, no nitrosyl linkage isomers were detected. Irradiation of **6** by UV light led to irreversible changes accompanied by the appearance of a well-defined band at 2225 cm^–1^ at room temperature, which is due to NO release.

EPR studies revealed that complexes **6** and **7** are not robust enough to be used for NO photorelease in solution. The stability of this type of complex might be increased by replacement of 1*H*-pyrazole with a stronger electron-donating ligand such as 3,5-dimethyl-1*H*-pyrazole. Other types of di- and polynuclear assemblies with an incorporated *trans-*[Ru(OH)NO(Hazole)_4_]^2+^ (Hazole = derivatives of 1*H*-indazole and 1*H*-pyrazole) moiety are envisioned.

Finally, this work demonstrates a broader point, which is that use of complexes such as **5** as a source of μ-hydroxido, μ_3_-oxido, and μ_4_-oxido groups is a viable and potentially general way to assemble heteropolynuclear metal complexes. These compounds maintain the essential properties searched for in nitrosyl compounds (i.e., PLI and/or NO release) and might serve also as platforms to combine magnetism and photoinduced changes.

## Experimental Section

The preparation of a Kralik solution^50^ and the synthesis of complexes (H_2_pz)[*trans*-Ru^III^Cl_4_(Hpz)_2_] (**1**) and *trans*-[Ru^III^Cl_2_(Hpz)_4_]Cl (**2**)^51,52^ are described in the Supporting Information.

### *trans*-[Ru^II^Cl_2_(Hpz)_4_] (**3**)

NaBH_4_ (2.0 g, 52.86 mmol) was added in portions to a suspension of **2** (3.23 g, 6.73 mmol) in methanol (125 mL), and the reaction mixture was stirred at room temperature for 1 h. The light brown precipitate was filtered off, washed with a small amount of water, methanol, and diethyl ether, and dried *in vacuo*. Yield: 2.19 g, 67.8%. ^1^H NMR (600 MHz, DMSO*-d*_6_): δ 12.11 (s, 1H), 7.68 (s, 1H), 7.38 (s, 1H), 6.18 (s, 1H). Anal. Calcd for C_12_H_16_Cl_2_N_8_Ru (*M*_r_ = 444.28): C, 32.44; H, 3.63; N, 25.22. Found: C, 32.60; H, 3.32; N, 25.11. Negative ion ESI-MS (in MeCN/MeOH + 1% H_2_O): *m*/*z* 442.89 [Ru^II^Cl_2_(Hpz)_4_ – H]^−^. IR (ATR, selected bands, ν_max_, cm^–1^): 3287, 1512, 1462, 1404, 1349, 1115, 1038, 849, 757, 599. UV–vis (CH_2_Cl_2_; λ_max_, nm (ε, M^–1^ cm^–1^)): 317 (17656), 394 (35).

### {[Ru(μ–OH)(μ-pz)_2_(pz)(NO)(Hpz)]_2_Mg} (**4**)

A suspension of NaNO_2_ (0.28 g, 4.06 mmol) and Mg(OH)_2_ (0.18 g, 3.09 mmol) in water (18 mL) was added to complex **3** (0.99 g, 2.23 mmol) in acetone/dicloromethane 1/1 (200 mL). The solution was refluxed with stirring overnight and cooled to room temperature. The organic phase was separated in a separatory funnel and washed with water (3 × 60 mL). The solvent was removed under reduced pressure, and the residue was suspended in a small amount of methanol (10 mL) and allowed to stand in the refrigerator overnight. The rose precipitate was filtered off, washed with a small amount of methanol and diethyl ether, and dried *in vacuo*. Yield: 0.66 g, 68.8%. Anal. Calcd for C_24_H_28_MgN_18_O_4_Ru_2_ (*M*_r_ = 859.04): C, 33.56; H, 3.29; N, 29.35. Found: C, 33.19; H, 3.26; N, 29.19. ESI-MS (in MeCN/MeOH + 1% H_2_O): positive ion, *m*/*z* 420.12 [Ru(OH)(NO)(pz)(Hpz)_3_]^+^, 861.1 (M + H)^+^; negative ion, *m*/*z* 859.0 (M – H)^−^. IR (ATR, selected bands, ν_max_, cm^–1^): 3597, 1847, 1483, 1409, 1381, 1352, 1274, 1160, 1050, 955, 874, 749, 673, 627, 568. UV–vis (CH_2_Cl_2_; λ_max_, nm (ε, M^–1^ cm^–1^)): 231 (37881), 497 (183). X-ray-diffraction-quality single crystals of **4** were grown in acetone/hexane (1/1).

### *trans*-[Ru(OH)(NO)(Hpz)_4_]Cl_2_ (**5**)

To a solution of **4** (0.45 g, 0.52 mmol) in acetone (200 mL) was added dropwise 3 M HCl (1.6 mL, 4.8 mmol), and the resulting beige-orange precipitate of crude **5** was filtered off immediately, washed with acetone, and dried *in vacuo*. Yield: 520 mg (ca. 100%). ESI MS (in MeOH): positive ion, *m*/*z* 420.17 [Ru(OH)(NO)(pz)(Hpz)_3_]^+^; negative ion, *m*/*z* 418.01 [Ru(OH)(NO)(pz)_3_(Hpz)]^−^. ^1^H NMR (600 MHz, DMSO-*d*_6_): δ 8.17 (d, *J* = 2.6 Hz, 1H), 7.66 (d, *J* = 2.2 Hz, 1H), 6.61 (t, *J* = 2.5 Hz, 1H). X-ray-diffraction-quality single crystals of **5·H**_**2**_**O** were grown in CHCl_3_/MeOH.

### [Fe_2_Ru^II^Cl_4_(μ_3_-O)(μ-OMe)(μ-pz)_2_(NO)(Hpz)_2_] (**6**)

To a solution of crude **5** (650 mg, 1.32 mmol) in methanol (45 mL) were added excess FeCl_3_·6H_2_O (2570 mg, 9.52 mmol) and excess K_2_CO_3_ (840 mg, 6.08 mmol), and the resulting suspension was heated at 70 °C overnight. The light brown unidentified precipitate (ca. 350 mg) was filtered, and the dark orange-brown filtrate was stored at 4 °C overnight. The formed colorless crystals of KCl were separated by filtration. Then the filtrate was concentrated to half of the original volume and again placed in the refrigerator at 4 °C for 1 day to give a new portion of KCl, which was separated by filtration. Further concentration of the filtrate led to the formation of a dark red-brown crystalline product, which was filtered off, washed with methanol, and dried *in vacuo*. Yield: 428 mg, 46.1%. Anal. Calcd for C_13_H_17_Cl_4_Fe_2_N_9_O_3_Ru (*M*_r_ = 701.9): C, 22.25; H, 2.44; N, 17.96. Found: C, 22.16; H, 2.45; N, 17.60. ESI MS (in MeCN/MeOH+1% H_2_O): positive ion, *m*/*z* 597.94 [M – (Hpz) – Cl]^+^, 631.83 (M – Cl – HCl)^+^, 633.90 [M – (OMe) – HCl]^+^; negative ion, *m*/*z* 665.83 (M – HCl – H)^−^. IR (ATR, selected bands, ν_max_, cm^–1^): 3298, 1881, 1367, 1276, 1113, 1048, 764, 683. X-ray-diffraction-quality crystals of **6** were selected directly from the isolated product formed upon concentration of the last filtrate.

### [Fe_2_RuCl_3_(μ_3_-O)(μ-OMe)(μ-pz)_3_(MeOH)(NO)(Hpz)][Fe_2_RuCl_3_(μ_3_-O)(μ-OMe)(μ-pz)_3_(DMF)(NO)(Hpz)] (**7**·MeOH·2H_2_O)

To a solution of crude **5** (250 mg, 0.51 mmol) in methanol (15 mL) were added excess FeCl_3_·6H_2_O (1053 mg, 3.9 mmol) and K_2_CO_3_ (345 mg, 2.5 mmol), and the resulting suspension was heated at 70 °C overnight. The light brown unidentified precipitate was filtered, and the dark orange-brown filtrate was stored at 4 °C overnight. The formed colorless crystals were separated by filtration. Then DMF (0.02 mL, 0.26 mmol) was added to the filtrate of **6** and the mixture was placed in the refrigerator at 4 °C for 1 day. A small amount of white crystals that formed was filtered off, and the filtrate was concentrated to half of the original volume and again placed in the refrigerator at 4 °C for 3 days to give a dark brown crystalline product, which was filtered off, washed with MeOH, and dried *in vacuo*. Yield: 160 mg, 41.6%. Anal. Calcd for **7·MeOH·2H**_**2**_**O**, C_31_H_51_Cl_6_Fe_4_N_19_O_11_Ru_2_ (*M*_r_ = 1504.09): C, 24.75; H, 3.42; N, 17.69; O, 11.70. Found: C, 24.54; H, 3.12; N, 17.33; O, 11.43. ESI MS (in MeCN/MeOH+1% H_2_O): positive ion, *m*/*z* 593.9 (M – HCl – H)^+^, 599.8 [M – (Hpz) + H]^+^, 629.84 (M–Cl)^+^; negative ion, 665.75 (M–H)^−^, where M is the unit [Fe_2_Ru^II^Cl_3_(μ_3_-O)(μ-OMe)(μ-pz)_3_(NO)(Hpz)]. IR (ATR, selected bands, ν_max_, cm^–1^): 3276, 1858, 1646, 1378, 1050, 756, 646. X-ray-diffraction-quality crystals of **7·MeOH** were selected directly from the isolated product.

### [Ga_3_RuCl_3_(μ_4_-O)(μ-OMe)_3_(μ-pz)_4_(NO)] (**8**)

To solution of crude **5** (260 mg, 0.53 mmol) in MeOH (5 mL) were added excess GaCl_3_ (550 mg, 3.13 mmol) in MeOH (11 mL) and K_2_CO_3_ (310 mg, 2.25 mmol). The resulting suspension was heated at 70 °C for 1.5 h. The next day, the first crop of the violet precipitate (ca. 400 mg) was filtered off and washed with a small amount of methanol. The red filtrate was concentrated to half of the original volume to give colorless crystals, which were separated by filtration. The mother liquor was allowed to stand at room temperature for 72 h to produce a second portion of violet and colorless crystals. This mixture was filtered off and washed with a small amount of water to remove the colorless crystals. The remaining violet crystals were collected and dried in air (45 mg). The first isolated portion of the violet precipitate (ca. 400 mg) was suspended in dimethylformamide (1 mL) and filtered, the violet filtrate was concentrated to 0.5 mL. Methanol (200 mL) was added to produce violet crystals, which were isolated after 72 h and dried in air (110 mg). Total yield: 155 mg, 35.6%. Anal. Calcd for C_15_H_21_Cl_3_Ga_3_N_9_O_5_Ru (*M*_r_ = 823.98): C, 21.86; H, 2.57; N, 15.29. Found: C, 22.04; H, 2.69; N, 15.00. ESI MS (in MeCN/MeOH+1% H_2_O): positive ion, *m*/*z* 759.80 [M – (OMe) – HCl]^+^, 793.76 [M – (OMe)]^+^; negative ion, *m*/*z* 859.56 (M + Cl)^−^. IR (ATR, selected bands, ν_max_, cm^–1^): 2944, 1862, 1494, 1431, 1386, 1290, 1169, 1055, 1025, 756, 696. Crystals of **8** suitable for an X-ray diffraction analysis were found in both isolated portions, as well as upon crystallization of **8** in DMF/MeOH.

### Crystallographic Structure Determination

Single-crystal X-ray diffraction (SC-XRD) measurements for **3**, **4**, **5·H**_**2**_**O**, **6**, **7·MeOH**, and **8** were carried out on Bruker D8 Venture and Bruker X8 APEX-II CCD diffractometers. Single crystals were positioned at 40, 35, 26, 27, 27, and 30 mm from the detector, and 541, 1021, 850, 1438, 500, and 2209 frames were measured, each for 1, 8, 60, 5, 15, and 10 s over 1, 0.5, −0.36, 0.5, −0.36, and 0.36° scan widths, respectively. The structures were solved by the SHELXS program and refined by full-matrix least squares on *F*^2^ with SHELXL.^89^ Non-H atoms were refined with anisotropic displacement parameters. H atoms were inserted in calculated positions and refined using a riding model. Crystal data, data collection parameters, and structure refinement details are given in Table S14, while pertinent geometric parameters are quoted in the legends to Figures 1–4 and Figures S8 and S23. Crystallographic data for the structures have been deposited with the Cambridge Crystallographic Data Centre, CCDC: 2107595 and 2098989–2098993.

### Mass Spectrometric Measurements

Mass spectrometry data were obtained from a Thermo LTQ-FT ion cyclotron resonance mass spectrometer equipped with a 7 T superconducting magnet. Electrospray ionization (ESI) measurements were carried out in a mass range between *m*/*z* 100 and 2000 using a transient of 1.5 s, resulting in a resolution of 200.000 at *m*/*z* 400.

### Magnetic Susceptibility Studies

Magnetic measurements were carried out on microcrystalline samples with a Quantum Design SQUID magnetometer (MPMS-XL). The variable-temperature (2–300 K) direct current (dc) magnetic susceptibility was measured under an applied magnetic field of 0.1 T. All data were corrected for the contribution of the sample holder and diamagnetism of the samples estimated from Pascal’s constants.^69,90^

### Mössbauer Studies

Mössbauer spectra were recorded on unenriched powders contained in Delrin cups at ca. 6 K on a strong-field Mössbauer spectrometer equipped with an Oxford Instruments Spectromag 4000 cryostat containing an 8 T split-pair superconducting magnet and at 82 K on a Mössbauer spectrometer equipped with a Janis SVT-400 cryostat. Both spectrometers were operated in a constant-acceleration mode in transmission geometry. The isomer shifts were referenced against that of a room-temperature metallic iron foil. Analyses of the data were performed with a locally written program.^91−93^

### Irradiation Studies

Infrared spectroscopy measurements in the range 400–4000 cm^–1^ and with a resolution of 2 cm^–1^ were performed on a Nicolet 5700 FT-IR spectrometer. The samples were ground, mixed with KBr, and pressed into pellets. Irradiation was performed by different light sources providing the wavelengths 655, 589, 556, 526, 476, 445, and 405 nm (lasers) and 810, 780, 590, 505, 470, and 365 nm (LEDs). The optical power was varied in the range 5–160 mW. For low-temperature measurements the KBr pellets were bonded by silver paste on the cold finger of a closed-cycle cryostat (Oxford Optistat V01). The cryostat allows controlling the temperature in the range of 9–320 K. UV–vis spectra were recorded at room temperature on a Varian CARY 4000 spectrometer, using KBr pellets as prepared for IR.

### Computational Details

The single-point and geometry optimization calculations of complex **6** have been performed in various spin states at the DFT level of theory using the B3LYP^94−96^ and BLYP^94,95^ functionals along with the def2-SVP basis set^97−100^ and with the utilization of the def2-SVP pseudopotential^101^ for Ru atoms in the Gaussian16 program package.^102^ The stability of the optimized structures was confirmed by the vibrational analysis. Restricted (closed-shell) and unrestricted high-spin, as well as broken symmetry (BS),^103−107^ approaches have been used to study the different spin-state energetics. The *J* (isotropic exchange) coupling is defined using the spin Hamiltonian in eq 1(108)
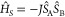
1where *S*_A_ and *S*_B_ are the individual spin center Hamiltonians. The *J* value was numerically evaluated using eq 2
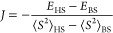
2where *E*_HS_ denotes the unrestricted high spin (HS) total energy, *E*_BS_ denotes the broken symmetry (BS) energy, and ⟨*S*^2^⟩ is the spin-squared expectation value of the relevant state, HS or BS. TDDFT calculations as implemented in Gaussian16 have been performed for 150 excited states, the TDDFT absorption spectrum has been processed with le_pychemy_go,^109^ and the TD transition state densities have been visualized with IQmol.^110^

Additional single-point calculations were carried out at the complete active space self-consistent field (CASSCF)^111−113^ level of theory with the utilization of the RIJCOSX^114^ approximation in the ORCA 4.2.0 software packages.^115−117^ The CASSCF results were based on an active space of 10 electrons in 10 orbitals, to account for the d electrons of two iron atoms of the neutral species. There were 9 electrons correlated in 10 orbitals in the case of the oxidized species. In the reduced species case, CASSCF calculations were based on an active space of 11 electrons in 11 orbitals, to account for the d electrons of two iron atoms, including the interaction within the ^1^[Ru^III^(NO)]^3+^ moiety. Furthermore, *n*-electron valence state perturbation theory of second order (NEVPT2)^118−121^ has been utilized to account for the dynamic electron correlation in CASSCF calculations. In the neutral and reduced species cases, low-spin states (*S* = 0, respectively *S* = 1/2) have been preferably targeted in the state-specific calculation. A single high-spin state has been chosen (*S* = 5) as the state-specific root in the case of the oxidized species.

The Molekel package^122^ was used for the visualization of the molecular orbitals and spin densities. The electronic structure was elucidated via localized orbitals (LOC) and a Mulliken population analysis as implemented in the Gaussian16 and ORCA 4.2.0 software packages^115−117^ using the B3LYP functional along with the def2-SVP and (def2)-TZVP basis sets. The basis set denoted (def2)-TZVP accounts for the def2-TZVP basis set for the Ru atom and the TZVP basis set for the Fe, Cl, N, O, C, and H atoms. The EPR (**g** tensor, ZFS splitting)^111,123,124^ and ^57^Fe Mössbauer parameters (isomer shifts and quadrupole splittings) were evaluated using the ORCA 4.2.0 software package according to Neese and co-workers.^125,126^ The B3LYP/(def2)-TZVP/*in vacuo* (BLYP/(def2)-TZVP/*in vacuo*) Mössbauer isomer shifts (δ) were estimated using the fitted trend line from Römelt^126^
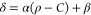
3where α = −0.298 au mm s^–1^, β = 1.118 mm s^–1^ and *C* = 11580 au as reported for this functional.

To further support the X-ray refined electronic structure, the quantum theory of atoms in molecules (QTAIM)^127^ has been used (using fchk or wfx files from Gaussian16) as implemented in the AIMAll package.^128^

### Experimental Electronic Structure of **6**

To examine the electronic structure of **6**, we have recorded additional synchrotron data at the Advanced Photon Source (APS). The diffraction intensities were collected by a Huber three-circle diffractometer equipped with a Pilatus 3× CdTe 1 M shutterless pixel array detector with λ = 0.41328 Å at 15 K maintained by the helium open-flow Cryostream system. A φ scan with a 0.3° interval was used for the data collection. Data reduction was done with EVAL15^129^ at the resolution of 1.12 Å^–1^ (d = 0.455 Å) and averaged by JANA2006.^130^ Crystallographic data are given in the Supporting Information.

The starting atomic coordinates and atom displacement parameters (ADPs) were taken from a routine SHELXL^89^ refinement, and all other refinements were carried out on *F*^2^ using the XD2015^131^ suite of programs. In addition, the determination of the atomic basins and bond critical point (BCP) descriptors was based on the framework of the quantum theory of atoms in molecules (QTAIM).^127^ Our experimental charge density refinement strategy was the same as that described in our previous studies,^88,132,133^ including the use of relativistic wave functions for all atoms.^134^ Multipoles up to hexadecapoles for Ru and Fe, octopoles for Cl, O, N, and C, and dipoles for H are used in the multipole model. Local symmetry constraints, as well as any chemical constraints, were not used. Attempts to apply the anisotropic secondary extinction correction^131^ were undertaken, but this correction was not found to be significant.

## Supporting Information Available

Syntheses of the concentrated “Kralik solution”, **1**, **2**, and **5**, high-resolution ESI mass spectra of [{Ru(OH)(pz)_3_(NO)(Hpz)}_2_M^III^]^+^ (M = Fe, Al) and [{Ru(OH)(pz)_3_(NO)(Hpz)}_2_Ba+H]^+^, low-resolution ESI mass spectra of **1**–**6**, **8**, ^1^H NMR spectra of **3** and **5**, IR spectra of **5**–**8**, ORTEP views of **3** and **5**, temperature-dependent magnetic susceptibility measurements on the polycrystalline heterotrinuclear clusters **6** and **7·MeOH**, Mössbauer spectra of **6**, full spectral range for RT irradiation of **6**, comparison of the initial state and after 400 min of 365 nm light irradiation with 100 mW, details of charge density and DFT calculations for **6**, and crystal data and details of data collection for **3**, **4**, **5·H**_**2**_**O**, **6**, **7·MeOH**, and **8** (PDF)

## Accession Codes

CCDC 2107595 and 2098989–2098993 contains the supplementary crystallographic data for this paper. These data can be obtained free of charge via www.ccdc.cam.ac.uk/data_request/cif, or by emailing data_request@ccdc.cam.ac.uk, or by contacting The Cambridge Crystallographic Data Centre, 12 Union Road, Cambridge CB2 1EZ, UK; fax: +44 1223 336033.
