# Supplementary material for: The Ruthenium Nitrosyl Moiety in Clusters: Trinuclear Linear μ-Hydroxido Magnesium(II)-Diruthenium(II), μ3-Oxido Trinuclear Diiron(III)–Ruthenium(II), and Tetranuclear μ4-Oxido Trigallium(III)-Ruthenium(II) Complexes
Source: Inorg Chem. 2021 Dec 28;61(2):950–67. doi: 10.1021/acs.inorgchem.1c03011 (PMC8767547; doi:10.1021/acs.inorgchem.1c03011)
Supplement: Supplementary file 1 — ic1c03011_si_001.pdf [file ic1c03011_si_001.pdf]

Supporting Information

for

The ruthenium nitrosyl moiety in clusters: trinuclear linear μ-hydroxido magnesium(II)-diruthenium(II), μ_3-oxido trinuclear diiron(III)-ruthenium(II) and tetranuclear μ_4-oxido trigallium(III)-ruthenium(II) complexes

Iryna Stepanenko,[†] Pavlo Mizetskyi,[†] Ewelina Orlowska,[†] Lukáš Bučinský,[#] Michal Zalibera,[#] Barbora Vénosová,[#,≠] Martin Clémancey,[‡] Geneviève Blondin,[‡] Peter Rapta,[#] Ghenadie Novitchi,[⊥] Wolfgang Schrader,[◊] Dominik Schaniel,[Δ] Yu-Sheng Chen,[⸸] Martin Lutz,[∇] Jozef Kožíšek,[#] Joshua Telser,[§] Vladimir B. Arion[†,*]

[†]*University of Vienna, Institute of Inorganic Chemistry, Währinger Strasse 42, A-1090 Vienna, Austria*

[#]*Institute of Physical Chemistry and Chemical Physics, Faculty of Chemical and Food Technology, Slovak University of Technology in Bratislava, Radlinského 9, SK-81237 Bratislava, Slovak Republic*

[‡] *Univ. Grenoble Alpes, CNRS, CEA, IRIG, LCBM, F-38000 Grenoble, France*

[⊥]*CNRS-LNCMI, 17 avenue des Martyrs, 38042 Grenoble Cedex, France*

[◊]*MPI für Kohlenforschung, Kaiser-Wilhelm-Platz 1, Mülheim an der Ruhr, Germany*

[Δ]*Université de Lorraine, CNRS, CRM2, 54506 Nancy, France*

[⸸]*NSF's ChemMATCARS, The University of Chicago, Lemont, 60439, Illinois, USA*

[∇]*Structural Biochemistry, Bijvoet Centre for Biomolecular Research, Utrecht University, 3584 CH Utrecht, The Netherlands*

[§]*Department of Biological, Physical and Health Sciences, Roosevelt University, 430 S. Michigan Avenue, Chicago, Illinois 60605, USA*

[≠]*Department of Physics, Faculty of Science, University of Ostrava, 30.dubna 22, 70103 Ostrava, Czech Republic*

*Corresponding author's E-mail address: vladimir.arion@univie.ac.at

Table of Contents

Syntheses of concentrated “Kralik solution”, 1, 2 and 5 S5

Characterization of the compounds S6

DFT and *ab initio* results of [6]$^+$ and [6]$^-$ S6

Figure S1. HRMS of [{Ru(OH)(pz)$_3$(NO)(Hpz)}$_2$Fe]$^+$: experimental (top) and calculated (bottom) isotopic distribution S9

Figure S2. HRMS of [{Ru(OH)(pz)$_3$(NO)(Hpz)}$_2$Al]$^+$: experimental (top) and calculated (bottom) isotopic distribution S9

Figure S3. HRMS of [{Ru(OH)(pz)$_3$(NO)(Hpz)}$_2$Ba+H]$^+$: experimental (top) and calculated (bottom) isotopic distribution S10

Figure S4. Negative ion ESI mass spectrum of **1** (in MeCN/MeOH+1% H_2O): full spectrum (top); experimental (middle) and calculated (bottom) isotopic distributions of the peak with *m/z* 379.72 attributed to [Ru^{III}Cl$_4$(Hpz)$_2$]$^–$ S11

Figure S5. Positive ion ESI mass spectrum of **2** (in MeCN/MeOH+1% H_2O): full spectrum (top); experimental (middle) and calculated (bottom) isotopic distributions of the peak with *m/z* 444.04 attributed to [Ru^{III}Cl$_2$(Hpz)$_4$]$^+$ S12

Figure S6. ^{1}H NMR (600 MHz, DMSO-*d*$_6$) spectrum of **3** S13

Figure S7. ESI mass spectrum of **3**: full spectrum in the positive ion mode (in MeCN/MeOH+1% H_2O) (A); full spectrum in the positive ion mode (in MeOH) (B); experimental (C) and calculated (D) isotopic distributions of the peak with *m/z* 442.89 attributed to [Ru^{II}Cl$_2$(Hpz)$_4$–H]$^–$ (in MeCN/MeOH+1% H_2O) S14

Figure S8. ORTEP view of *trans*-[Ru^{II}Cl$_2$(Hpz)$_4$] (**3**). S15

Figure S9. Positive ion ESI mass spectrum of **4** (in MeCN/MeOH+1% H_2O): full spectrum (top); experimental (middle) and calculated (bottom) isotopic distributions of the peak with *m/z* 861.09 attributed to [M+H]$^+$ S16

Figure S10. Negative ion ESI mass spectrum of **4** (in MeCN/MeOH+1% H_2O): full spectrum (top); experimental (middle) and calculated (bottom) isotopic distributions of the peak with *m/z* 859.00 attributed to [M–H]$^–$ S17

Figure S11. Positive ion ESI mass spectrum of **5** (in MeOH): full spectrum (top); experimental (middle) and calculated (bottom) isotopic distributions of the peak with *m/z* 420.17 attributed to [Ru(OH)(NO)(pz)(Hpz)$_3$]$^+$ S18

Figure S12. Negative ion ESI mass spectrum of **5** (in MeOH): experimental (top) and calculated (bottom) isotopic distributions of the peak with *m/z* 418.01 attributed to [Ru(OH)(NO)(pz)$_3$(Hpz)]$^–$ S19

Figure S13. ^{1}H NMR (600 MHz, DMSO-*d*$_6$) spectrum of **5** S19

Figure S14. FTIR spectrum of crude **5** S20

Figure S15. Negative ion ESI mass spectrum of **6** (in MeCN/MeOH+1% H_2O): full spectrum (top); experimental (middle) and calculated (bottom) isotopic distributions of the peak with *m/z* 665.85 attributed to [M–HCl–H]$^–$ S21

Figure S16. Positive ion ESI mass spectrum of **6** (in MeCN/MeOH+1% H_2O): full spectrum (top); experimental (middle) and calculated (bottom) isotopic distributions of the peak with *m/z* 597.92 attributed to [M–(Hpz)–Cl]$^+$ S22

Figure S17. Negative ion ESI mass spectrum of **7** (in MeCN/MeOH+1% H_2O): the peak with *m/z* 665.75 attributed to $[M–H]^–$, where M is the unit $[Fe_2Ru^{II}Cl_3(\mu_3$-O)(μ-OMe)(μ-pz)$_3$(NO)(Hpz)] S23
Figure S18. FTIR spectrum of **6** S23
Figure S19. FTIR spectrum of **7** S24
Figure S20. Positive ion ESI mass spectrum of **8** (in MeCN/MeOH+1% H_2O): full spectrum (top); experimental (middle) and calculated (bottom) isotopic distributions of the peak with *m/z* 793.76 attributed to $[M–(OMe)]^+$ S25
Figure S21. Negative ion ESI mass spectrum of **8** (in MeCN/MeOH+1% H_2O): full spectrum (top); experimental (middle) and calculated (bottom) isotopic distributions of the peak with *m/z* 859.58 attributed to $[M+Cl]^–$ S26
Figure S22. FTIR spectrum of **8** S27
Figure S23. ORTEP view of the cation $[Ru(OH)NO(Hpz)_4]^{2+}$ in **5** with thermal ellipsoids drawn at 30% probability level S26
Figure S24. Temperature dependent magnetic susceptibility measurements on the polycrystalline heterotrinuclear clusters **6** and **7·MeOH** S28
Figure S25. 82 K Mössbauer spectrum of a powder sample of **6**. S29
Figure S26. Experimental (hatched bars) Mössbauer spectrum recorded on a powder sample of **6** at 6 K using a 7 T external magnetic field applied parallel to the γ-beam S30
Figure S27. Full spectral range for RT irradiation of **6**: comparison of initial state and 400 min of 365 nm light irradiation with 100 mW S31
Figure S28. Frontier α orbitals and spin densities of ^{10}[**6**]$^+$ and ^{10}[**6**]$^–$ S32
Figure S29. Synchrotron result of X-ray diffraction study of **6** S34
Figure S30. 3D plot of the Laplacian of electron density of **6** at the isosurface value of 90 e/Å^5, generated using the method of Hübschle and Dittrich S34
Figure S31. ORTEP plots of the asymmetric units of **6**. Displacement ellipsoids are at 30% probability level. Symmetry code used: *) x – ½, –y + 3/2, z – ½; **) x – 1, y, z; ***) 1 – x, 1 – y, 1 – z S35
Figure S32. 3D plot of the Laplacian of electron density of **6** around N1 at the isosurface value of 55 e/Å^5, generated using the method of Hübschle and Dittrich S35
Figure S33. 3D plot of the Laplacian of electron density of **6** at the isosurface value of 16 e/Å^5, generated using the method of Hübschle and Dittrich. Symmetry code used: *) x – ½, –y + 3/2, z – ½; **) x – 1, y, z. S36
Figure S34. 3D plot of the Laplacian of electron density of **6** around Fe1/Fe2 at the isosurface value of 15.5 e/Å^5, generated using the method of Hübschle and Dittrich S36
Table S1. B3LYP, BLYP, CAS and NEVPT2 (def2-SVP basis set) total energies of spin states of the studied complex **6** S37
Table S2. Relative B3LYP and BLYP def2-SVP energies (ΔE = E_S), $\langle S^2 \rangle$ expectation values, *J* coupling of spin states, relative CASSCF and NEVPT2 def2-SVP energies of the studied species of [**6**]$^+$ and [**6**]$^-$, note that *J* coupling is given with respect to eqs. (2) and (3) S37

Table S3. Selected B3LYP/def2-SVP bond distances (Å) and angles (°) of chosen [**6**] species S38
Table S4a. B3LYP/def2-SVP MPA charge and spin of neutral $[\mathbf{6}]^0$ and oxidized $[\mathbf{6}]^+$ species for the chosen spin states S39
Table S4b. B3LYP/def2-SVP MPA charge and spin of the reduced $[\mathbf{6}]^-$ complex for the chosen spin states S39
Table S5. B3LYP/def2-SVP Mulliken d-orbital and s-orbital populations of various neutral and charged species of **6** S40
Table S6. B3LYP/TZVP and CASSCF/def2-SVP Mulliken d-orbital populations of various neutral and charged species of **6** S41
Table S7. B3LYP/TZVP Mössbauer parameters for Fe1 and Fe2 centers of various charged species of **6** S42
Table S8. Calculated **g**-tensor and ZFS parameters of various neutral and charged species of **6** S42
Table S9. Crystallographic data of **6** measured for charge density calculations. S43
Table S10. Selected interatomic distances [Å] and angles [°] in **6** S44
Table S11. AIM electron density properties at bond critical points in **6**: comparison between experiment (1st row) and density functional theory (experimental geometry) calculations (2nd row) S45
Table S12. Population of the *d*-orbitals [e-] on central atoms in **6**: comparison between experiment (1st row) and density functional theory (experimental geometry) S46
Table S13. AIM atomic charges and atomic volumes in **6**: comparison between experiment (1st row) and density functional theory (experimental geometry) calculation S47
Table S14. Crystal data and details of data collection for **3**, **4**, **5·H_2O**, **6**, **7·MeOH** and **8**. S48
References S49

Syntheses of concentrated "Kralik solution", 1, 2 and 5

Concentrated "Kralik solution"[1]: commercial $RuCl_3$·hydrate (8 g) was dissolved in a mixture of ethanol (50 mL) and 12M HCl (50 mL) and refluxed for 1 h. After ethanol removal on a rotary evaporator, the dark red solution was filtered and diluted with 12M HCl to a volume of 100 mL (0.028 mmol of Ru in 100 mL).

(H_2pz)[*trans*-$Ru^{III}Cl_4(Hpz)_2$] (**1**). Pyrazole (20 g, 0.29 mol) was added to the concentrated "Kralik solution" (100 ml, 0.028 mol of Ru) and stirred at room temperature for 72 h. The formed dark wine-red precipitate was filtered off, washed with a small amount of cold ethanol, then diethyl ether and dried in vacuo. Yield: 9.0 g, 71%. ESI-MS (in MeCN/MeOH+1% H_2O): negative: *m/z* 243.65 $[RuCl_4]^-$, 311.68 $[RuCl_4(Hpz)]^-$, 379.72 $[RuCl_4(Hpz)_2]^-$.

[*trans*-$Ru^{III}Cl_2(Hpz)_4$]Cl (**2**)**.** A mixture of **(H_2pz)[*trans*-$Ru^{III}Cl_4(Hpz)_2$]** (**1**) (4.5 g, 0.01 mol) and pyrazole (1.35g, 0.02 mol) in ethanol/water (v/v 7/3, 180 mL) was heated under reflux for 7 h. The solution was evaporated to ca. 50 mL and left to stand at 4 °C overnight. The dark-red precipitate was filtered off, washed with water and dried in vacuo. Yield: 3.2 g, 66.4%. ESI-MS (in MeCN/MeOH+1%H_2O): positive: *m/z* 444.04 $[RuCl_2(Hpz)_4]^+$; negative 441.84 $[RuCl_2(Hpz)_4{-}2H^+]^-$.

Crude product 5. The crude product **5** is a mixture of two complexes, complex **5** and $[Mg(H_2O)_6][Ru(Hpz)_4(NO)(OH)]Cl_4$. Attempts to crystallize the second complex failed. However, we succeeded to crystallize the complex $[Mg(H_2O)_6][Ru(Hpz)_4(NO)(OH)](SO_4)_2$, which was obtained by a similar procedure: hydrolysis of complex **4** using diluted sulfuric acid instead of hydrochloric acid.

The formation of the two complexes follows the reactions:

$$Ru_2Mg(OH)_2(NO)_2(pz)_6(Hpz)_2\ (\mathbf{4}) + 6HCl = 2[Ru(Hpz)_4(NO)(OH)]Cl_2\ (\mathbf{5}) + MgCl_2$$

$$[Ru(Hpz)_4(NO)(OH)]Cl_2\ (\mathbf{5}) + MgCl_2 + 6H_2O = [Mg(H_2O)_6][Ru(Hpz)_4(NO)(OH)]Cl_4$$

or

$$Ru_2Mg(OH)_2(NO)_2(pz)_6(Hpz)_2\ (\mathbf{4}) + 6HCl + 6H_2O = [Ru(Hpz)_4(NO)(OH)]Cl_2\ (\mathbf{5}) + [Mg(H_2O)_6][Ru(Hpz)_4(NO)(OH)]Cl_4$$

This crude product **5** was used directly for the synthesis of complexes **6**–**8** and reproducibility of the results was confirmed. The formation of $[Mg(H_2O)_6][Ru(Hpz)_4(NO)(OH)]Cl_4$ along with **5**

had no effects on the next steps and therefore the crude product **5** was used as is. Crystallization of crude **5** in MeOH or $CHCl_3$/MeOH afforded crystals of $[Ru^{II}(Hpz)_4(NO)(OH)]Cl_2$ (**5**), which were studied by SC-XRD, ESI MS and ^{1}H NMR.

Characterization of the compounds. Elemental analyses were carried out in a Carlo-Erba microanalyzer at the Microanalytical Laboratory of the University of Vienna. Electrospray ionization mass spectrometry (ESI-MS) was carried out with amaZon speed ETD Bruker instrument. Expected and experimental isotope distributions were compared. UV–vis spectra were measured on Perkin Elmer UV–vis spectrophotometer Lambda 35 in the 240 to 700 nm window. IR spectra were recorded on a Bucker Vertex 70 Fourier transform IR spectrometer (4000–600 cm^{-1}) using the ATR technique. ^{1}H NMR spectra were acquired on a Bruker AV NEO 500 or AV III 600 spectrometers in DMSO-d_6 at 25 °C and in $CDCl_3$ at –40 °C.

DFT and *ab initio* results of [6]$^+$ and [6]$^-$

Energetics. In the one-electron oxidized cation ^{10}[**6**]$^+$, the m_S = 10 spin multiplicity was found to be energetically preferred, see Tables S1 and S2. However, the BS doublet state (^{2}[**6**]$^+$) is close in energy to the m_S = 10 spin state (^{10}[**6**]$^+$). In the reduced anion case, three spin states are found energetically close to each other (^{12}[**6**]$^–$, ^{10}[**6**]$^–$, ^{2}[**6**]$^–$). The BS doublet state (^{2}[**6**]$^–$) is below the high spin states ^{10}[**6**]$^–$, and ^{12}[**6**]$^–$, see Tables S1 and S2. The B3LYP-calculated energy difference between ^{2}[**6**]$^+$ and ^{10}[**6**]$^+$ is 6.8 kJ/mol and *J* coupling is found to be 0.339 kJ/mol (28.3 cm^{-1}), see Table S2. In the ^{12}[**6**]$^–$ and ^{2}[**6**]$^–$ case, we have observed small energy shift (6.6 and –7.4 kJ/mol) from ^{10}[**6**]$^–$. The ^{12}[**6**]$^–$ vs ^{2}[**6**]$^–$ *J* coupling is –0.462 kJ/mol (–38.6 cm^{-1}), see Table S2, while ^{10}[**6**]$^–$ vs ^{2}[**6**]$^–$ *J* coupling is –0.367 kJ/mol (–30.7 cm^{-1}). Among the most relevant [**6**]$^+$ and [**6**]$^–$ spin states, the major differences between the two functionals employed can be observed for the ^{12}[**6**]$^–$ and ^{12}[**6**]$^+$ species where the absolute energy difference with respect to ^{10}[**6**] is considerably larger in the BLYP case. In addition, BLYP predicts a smaller value of antiparallel *J* coupling for ^{2}[**6**]$^–$ comparing B3LYP. In the case of the oxidized and reduced species, the NEVPT2 J couplings equal 0.334 kJ/mol (27.9 cm–1) and –0.413 kJ/mol (–34.5 cm–1), respectively. In CASSCF calculations, these values are underestimated by more than a factor of two: 0.140 kJ/mol (11.7 cm–1) and –0.189 kJ/mol (–15.8 cm–1), respectively.

Optimized geometries. In the case of the oxidized and reduced species of **6**, the energetically favored m_S = 10 high spin state, m_S = 12 high spin state, and the BS doublet spin state have been

taken into account, see Table S2. No significant differences in bond lengths and bond angles are observed comparing the neutral and oxidized species, see Table S3. Changes in the geometry upon reduction are much more obvious when compared to the neutral geometry. The Ru–N9, Ru–O1 bond lengths become significantly longer and the Fe–Fe, O1–Fe1, O1–Fe2 bond lengths (distances) become shorter, see Table S3. All the reduced species have a bent structure of the [Ru(NO)] moiety, with the Ru–N9–O2 angle of ~ 145° due to the Ru-(NO) character of the LUMO (see Figure S28).

Electronic structure characterization and the frontier orbitals. The iron center is oxidized in the case of the oxidized species ^{10}[**6**]$^{+}$, see Tables S3, S4a, and S5. In the case of reduction, the bent ^{2}[Ru^{III}-NO]$^{2+}$ ({RuNO}^{7}) moiety is the electron acceptor locus, see Tables S3, S4b, and S5. For the oxidized ^{12}[**6**]$^{+}$ species, the ruthenium center becomes oxidized, i.e. d_{xy}(Ru) α and β AO populations are 0.983 and 0.629 (see Table S5), respectively, instead of ideally 2 and 0 which appears a natural feature of the ^{2}[Ru^{IV}-NO]$^{4+}$ moiety. Still, this ^{2}[Ru^{IV}-NO]$^{4+}$ moiety remains linear, i.e. the Ru-N1-O1 angle is close to 180° and the spin density is found localized on Ru and the heterocycles. In terms of Enemark-Feltham notation, this moiety contains either Ru^{IV} (4d^{4}, S_{Ru} = 0) bonded to NO^{0} (π*^{1}, S_{NO} = 1/2) or Ru^{III} (4d^{5}, S_{Ru} = 1/2) bonded to NO^{+} (π*^{0}, S_{NO} = 0), but both giving a total spin S = 1/2. Still, the Enemark-Feltham notation seems not appropriate, because it is found here that the non-bonding 4d_{xy}(Ru) contains the unpaired electron. On the contrary, one of the iron (Fe1) centers is oxidized in ^{10}[**6**]$^{+}$, which is being energetically preferred (see Figure S28, Tables S2, S4a and S5) and the ^{1}[Ru^{III}-NO]$^{3+}$ moiety remains rather unaffected. The Fe1 center oxidation can be seen in the lower α d_{xz} AO population, see Table S5. In addition, the Fe1 spin population is 3.5 while Fe2 population remains close to 4.0, see Table S4a. In the case of BS ^{2}[**6**]$^{+}$ species, the Fe1 spin becomes flipped so the spin population is –3.5 (i.e., there is an excess of β spin density on the Fe1 center similar to BS ^{1}[**6**]^{0}, see Table S4a). These indicate that Fe1 atom in ^{10}[**6**]$^{+}$ and ^{2}[**6**]$^{+}$ has been oxidized to a formal oxidation state IV (electron configuration d^{4}), see Tables S4a and S5.

The ^{1}[Ru^{III}-NO]$^{3+}$ moiety is linear for all neutral **6** and oxidized **6**$^{+}$ geometries, showing no effect of spin state or oxidation on the 6 electrons (3 π electrons of NO and 3 d_{xz} and d_{yz} electrons of Ru) which are essential within this moiety. On the contrary, the ^{2}[Ru^{III}-NO]$^{2+}$ moiety becomes bent in the case of the anion **6**$^{–}$, thus a further electron enters the π_x(NO), π_y(NO), d_{xz}(Ru) and d_{yz}(Ru) manifold, see below. Furthermore, the spin population on the Ru-NO moiety in the

reduced species is approximately one, which confirms that this moiety is reduced indeed (while the spin population of Fe centers does not change), see Table S4b. For ^{12}[**6**]$^{–}$, the entire spin is up (high spin state), see Table S4b. In the case of the intermediate BS ^{10}[**6**]$^{–}$ spin state, the ^{2}[Ru^{III}-NO]$^{2+}$ spin density is antiparallel to the two spin up iron centers, see Table S4b and Figure S28. In addition, the Fe1 center has the spin flipped in the case of ^{2}[**6**]$^{–}$, see Table S4b.

B3LYP/def2-SVP spind density and frontier α HOMO-1, HOMO, LUMO, LUMO+1, LUMO+2 orbitals for ^{10}[**6**]$^{+}$ and ^{10}[**6**]$^{–}$ complex are depicted in Figure S28. The HOMO of ^{10}[**6**]$^{+}$ and ^{10}[**6**]$^{–}$ has a pz-Ru-pz antibonding character being similar to HOMO-1 of ^{11}[**6**]^{0}. In the case of ^{10}[**6**]$^{+}$, one of the Fe centers is oxidized, hence the LUMO orbital reflects this. On the contrary, for the reduced ^{10}[**6**]$^{–}$ species HOMO-1 has a considerable [Ru-NO] moiety character and one finds still the two lowest virtual orbitals with the Ru-NO antibonding nature (note, the two lowest unoccupied frontier orbitals in the neutral species that are at the Ru-NO moiety).

EPR (spin Hamiltonian) parameters. The theoretical spin Hamiltonian parameters (**g**- tensor values and ZFS values) of different species of **6** are compiled in Table S8. The B3LYP/TZVP calculation yields g values shifted from the free electron 2.0023 *g*-value. The g-values calculated for the B3LYP and BLYP levels of theory do not differ significantly. Still, the *g*-value of [**6**] is underestimated when compared to the experiment. The *g*-values from CASSCF and NEVPT2 calculations for species of **6** chosen in Table S8 are close to the free electron *g* value, which points to a larger localization of the spin density on the iron centers for the *ab initio* methods (exact exchange). The ZFS B3LYP parameters are found positive and below 1 cm$^{–1}$. In the case of BLYP (a non-hybrid or pure DFT functional) *D* values are larger than B3LYP ones which mostly leads to results closer to experimental fittings, i.e. B3LYP tends to underestimate the results by a factor of at least two when comparing to BLYP.[2] Based on reported studies, BLYP (a non-hybrid or pure DFT functional) calculated *D* values are larger than those from B3LYP. On the other hand, the BLYP level of theory tends to predict experimentally derived *D* parameters less accurately in comparison with the CASSCF/NEVPT2 results.2

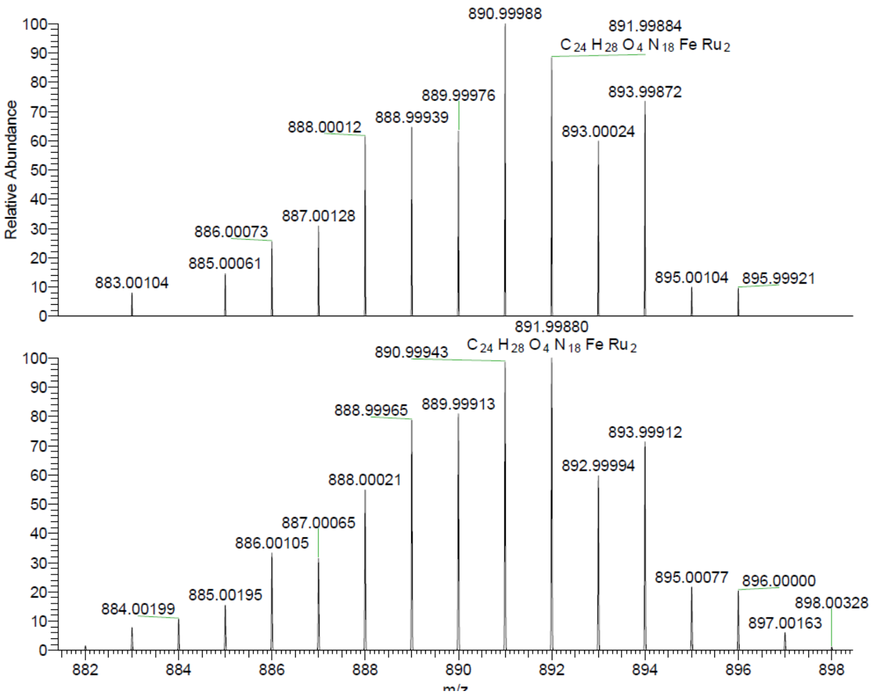

Figure S1. HRMS of [{Ru(OH)(pz)$_3$(NO)(Hpz)}$_2$Fe]$^+$: experimental (top) and calculated (bottom) isotopic distributions.

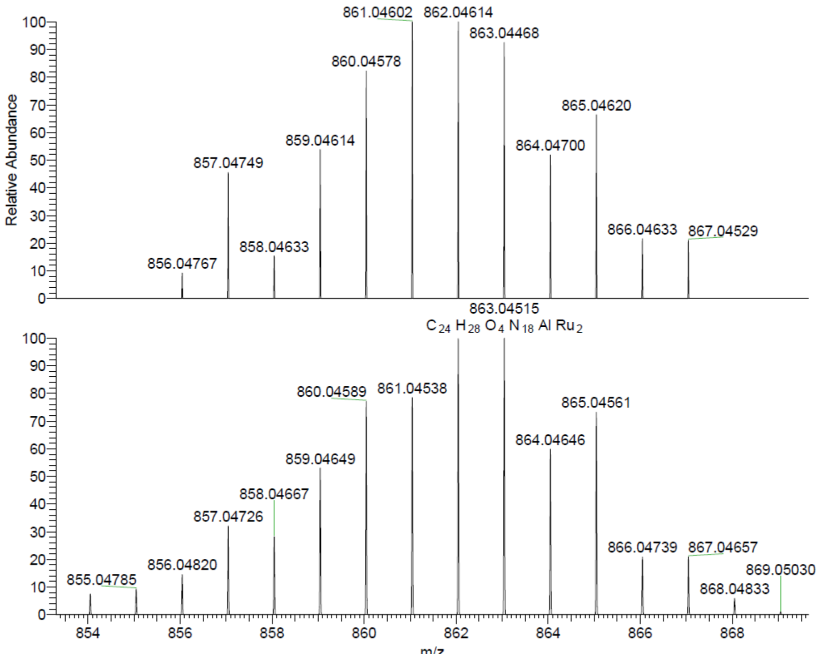

Figure S2. HRMS of [{Ru(OH)(pz)$_3$(NO)(Hpz)}$_2$Al]$^+$: experimental (top) and calculated (bottom) isotopic distributions.

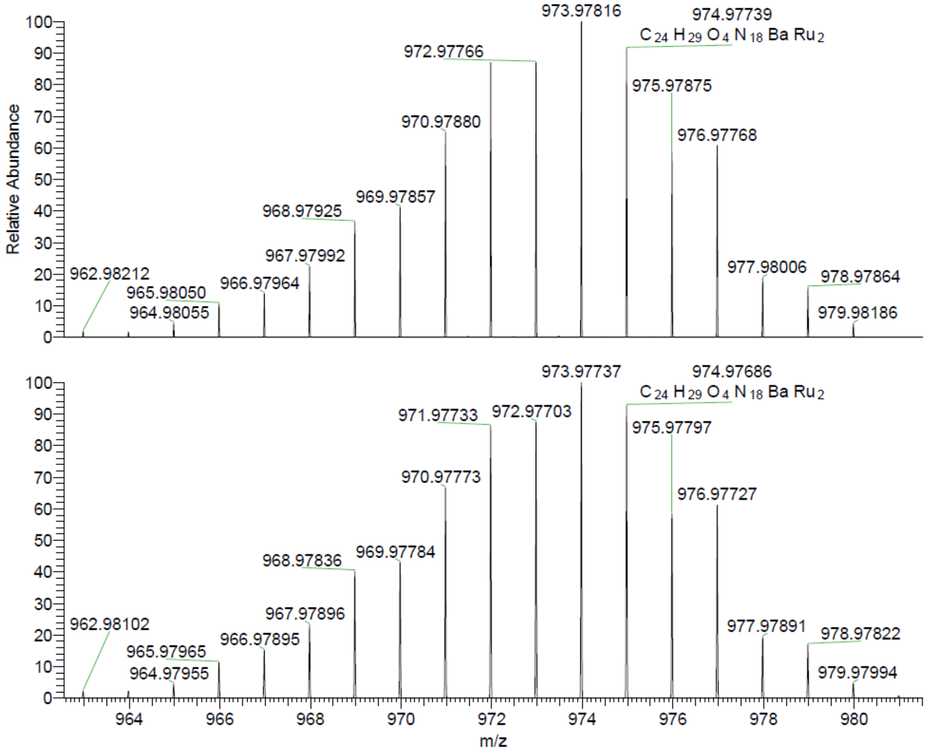

Figure S3. HRMS of $[\{Ru(OH)(pz)_3(NO)(Hpz)\}_2Ba+H]^+$: experimental (top) and calculated (bottom) isotopic distributions.

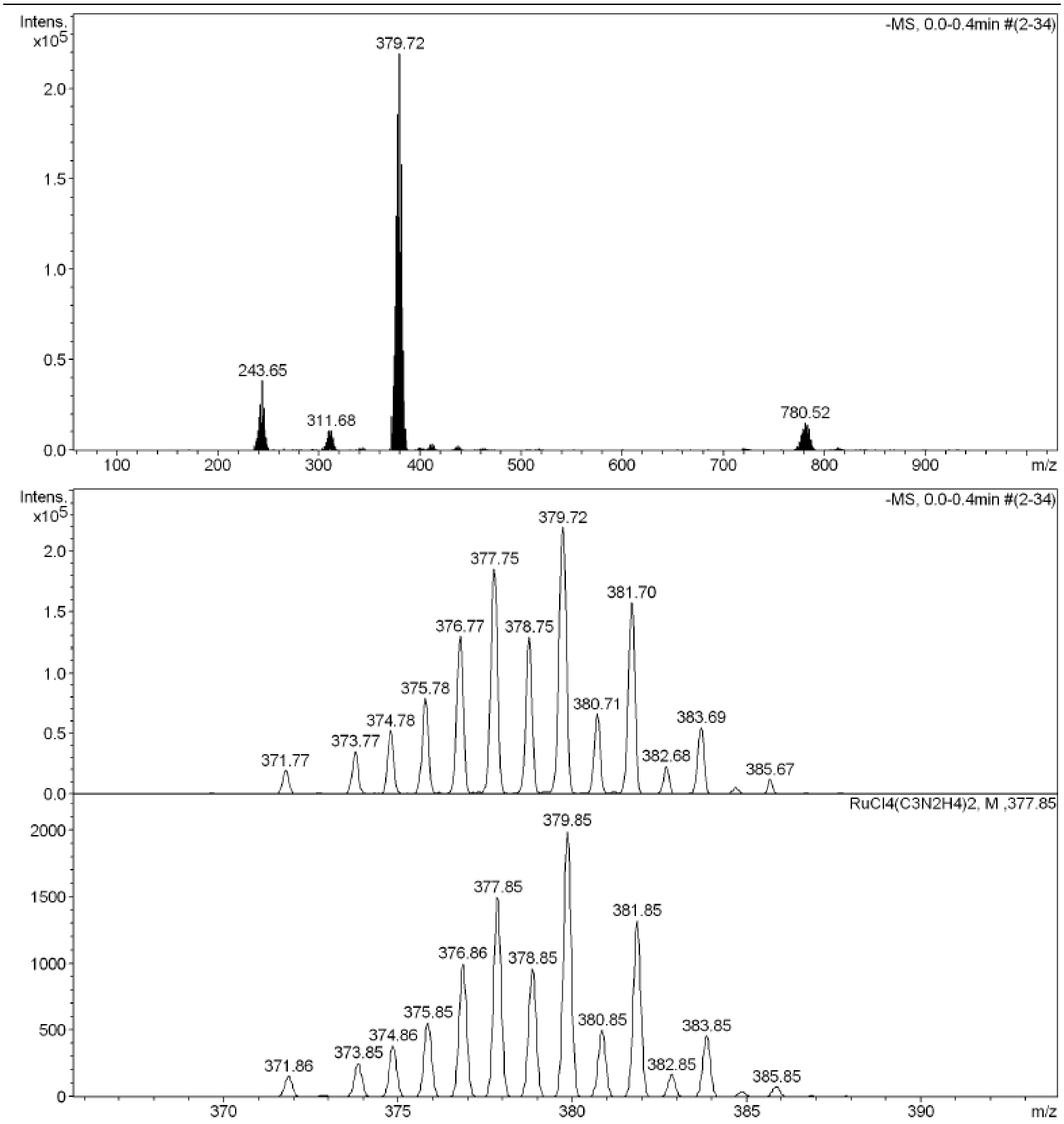

Figure S4. Negative ion ESI mass spectrum of **1** (in MeCN/MeOH+1% H_2O): full spectrum (top); experimental (middle) and calculated (bottom) isotopic distributions of the peak with *m/z* 379.72 attributed to $[Ru^{III}Cl_4(Hpz)_2]^-$.

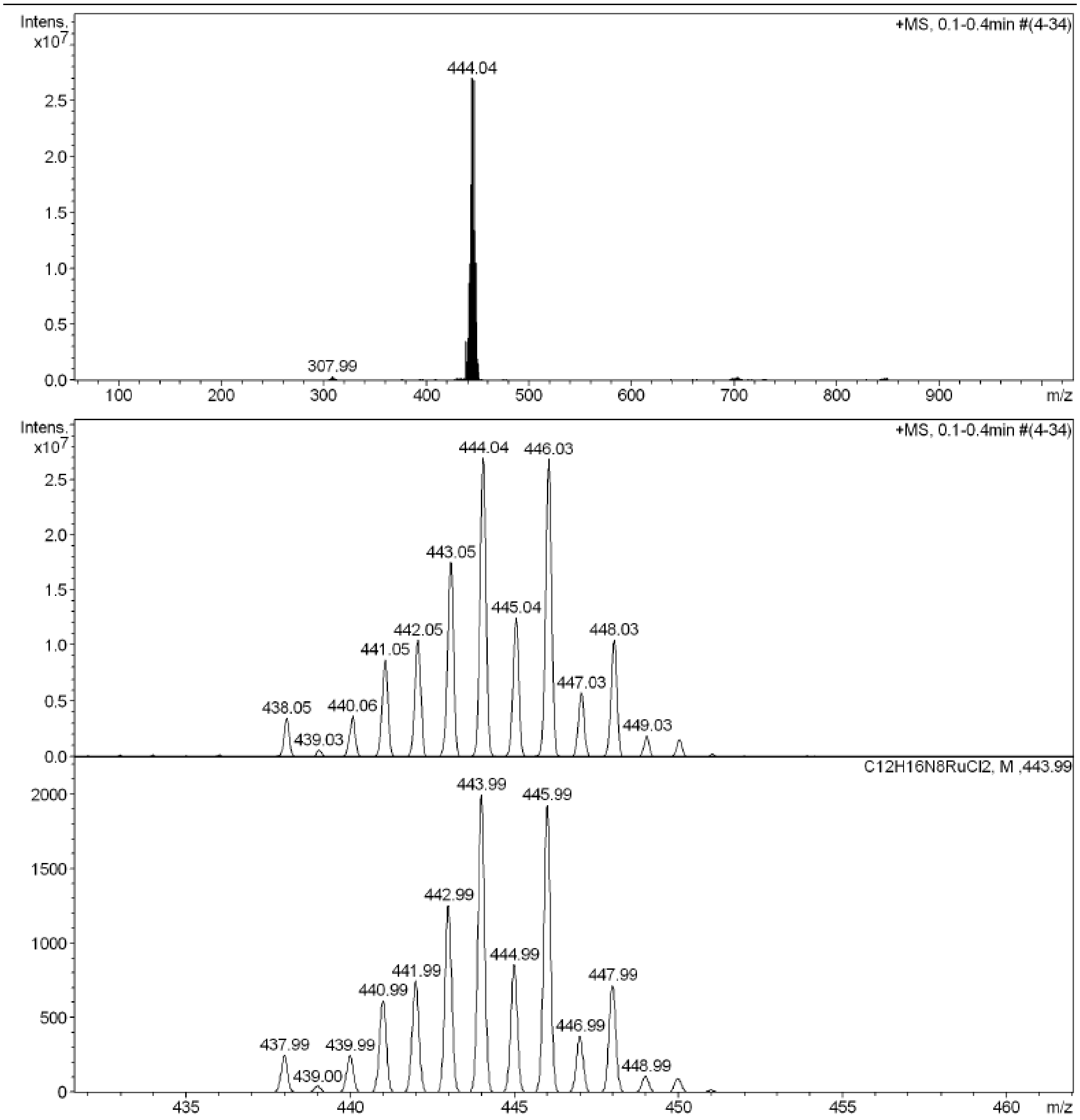

Figure S5. Positive ion ESI mass spectrum of **2** (in MeCN/MeOH+1% H_2O): full spectrum (top); experimental (middle) and calculated (bottom) isotopic distributions of the peak with *m/z* 444.04 attributed to $[Ru^{III}Cl_2(Hpz)_4]^+$.

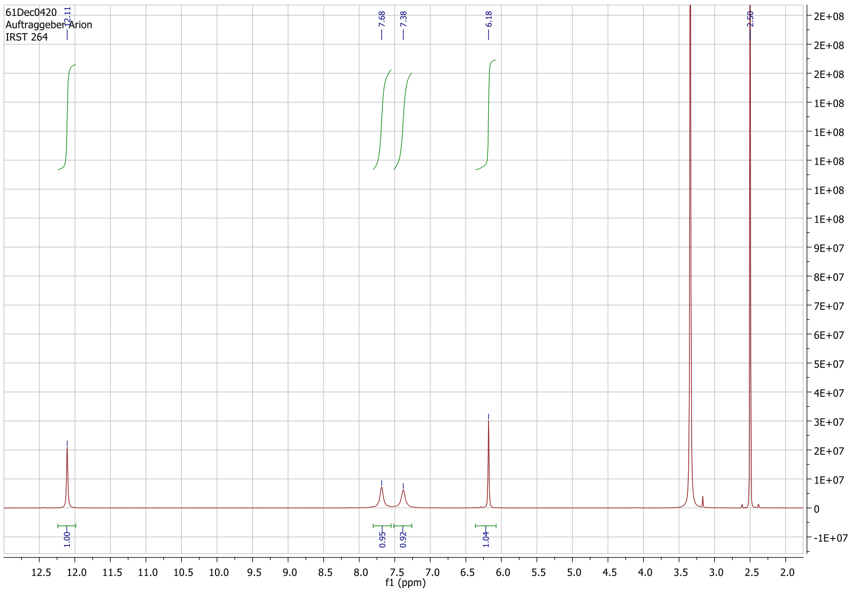

Figure S6. ^{1}H NMR (600 MHz, DMSO-d_6) spectrum of **3**.

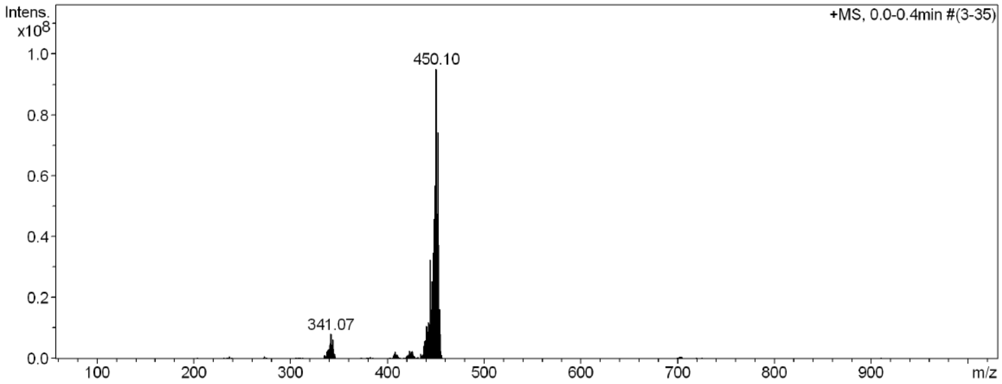

A

Positive ion ESI MS (in MeCN/MeOH+1% H_2O): *m/z* 450.10 $[Ru^{II}Cl(Hpz)_4(CH_3CN)]^+$; 341.07 $[Ru^{II}Cl(Hpz)_3]^+$

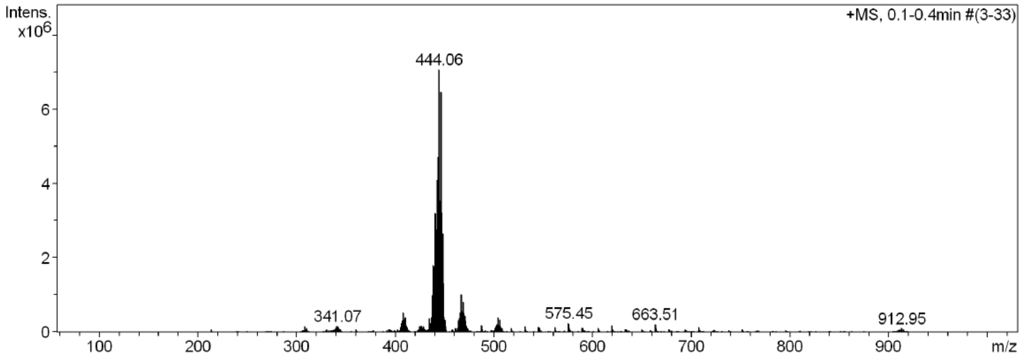

B

Positive ion ESI MS (in MeOH): *m/z* 444.06 $[Ru^{III}Cl_2(Hpz)_4]^+$

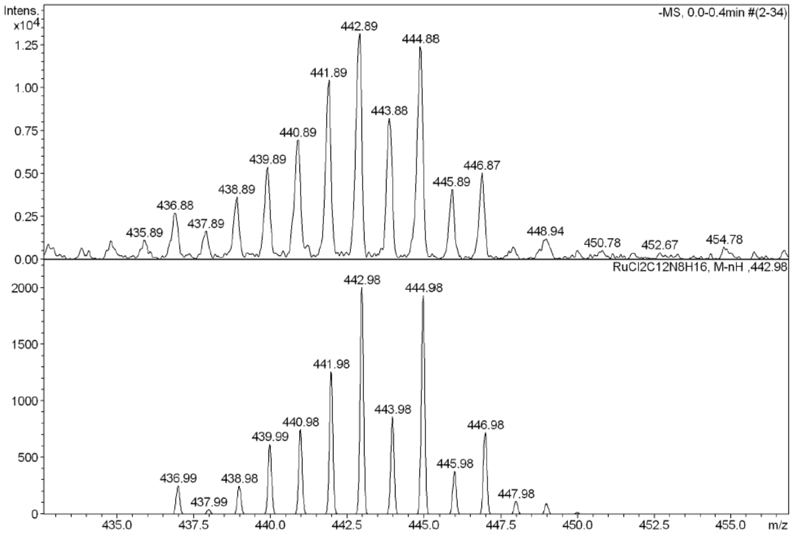

C

D

Figure S7. ESI mass spectrum of **3**: full spectrum in the positive ion mode (in MeCN/MeOH+1% H_2O) (A); full spectrum in the positive ion mode (in MeOH) (B); experimental (C) and calculated (D) isotopic distributions of the peak with *m/z* 442.89 attributed to $[Ru^{II}Cl_2(Hpz)_4–H]^–$ (in MeCN/MeOH+1% H_2O).

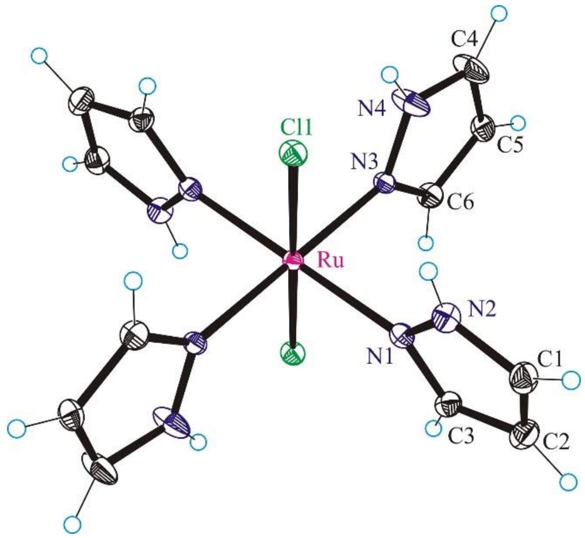

Figure S8. ORTEP view of *trans*-[Ru^{II}Cl$_2$(Hpz)$_4$] (**3**). Selected bond distances (Å) and bond angles (°): Ru1–N1 2.0749(15), Ru1–N3 2.0717(15), Ru–Cl1 2.4474(4); N1–Ru–N3 87.68(5).

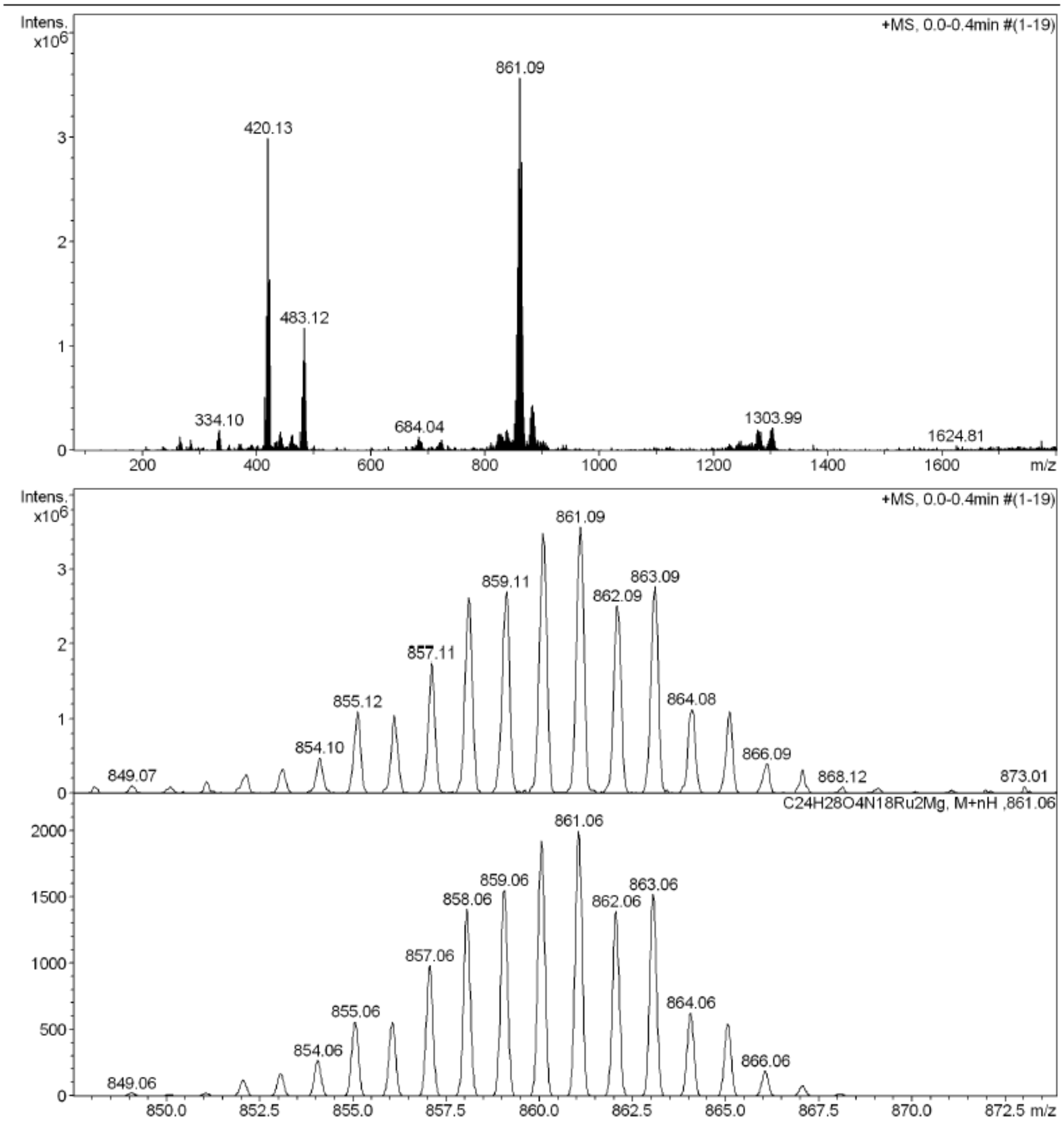

Figure S9. Positive ion ESI mass spectrum of **4** (in MeCN/MeOH+1% H_2O): full spectrum (top); experimental (middle) and calculated (bottom) isotopic distributions of the peak with *m/z* 861.09 attributed to $[M+H]^+$.

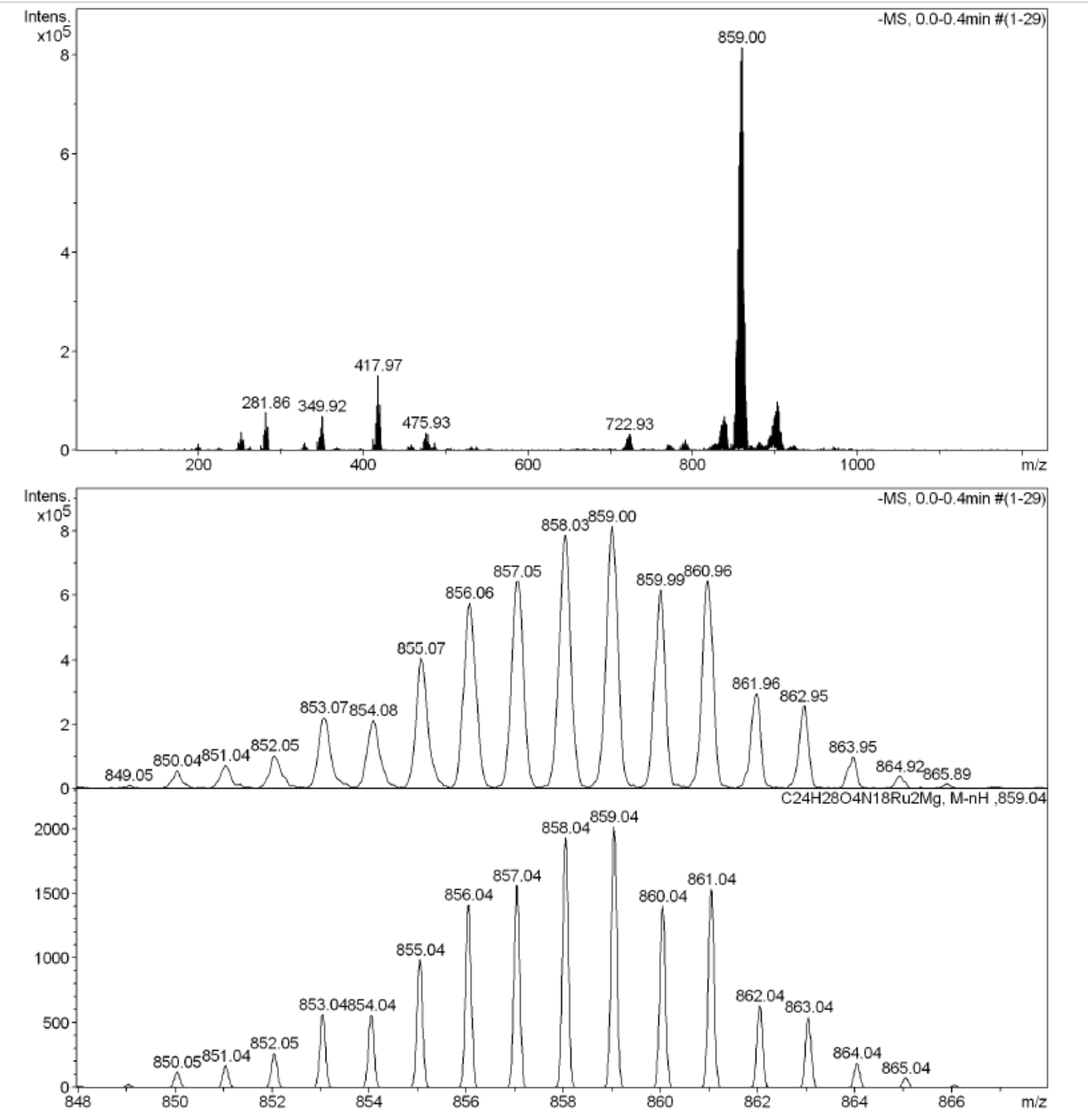

Figure S10. Negative ion ESI mass spectrum of **4** (in MeCN/MeOH+1% H_2O): full spectrum (top); experimental (middle) and calculated (bottom) isotopic distributions of the peak with *m/z* 859.00 attributed to $[M–H]^-$.

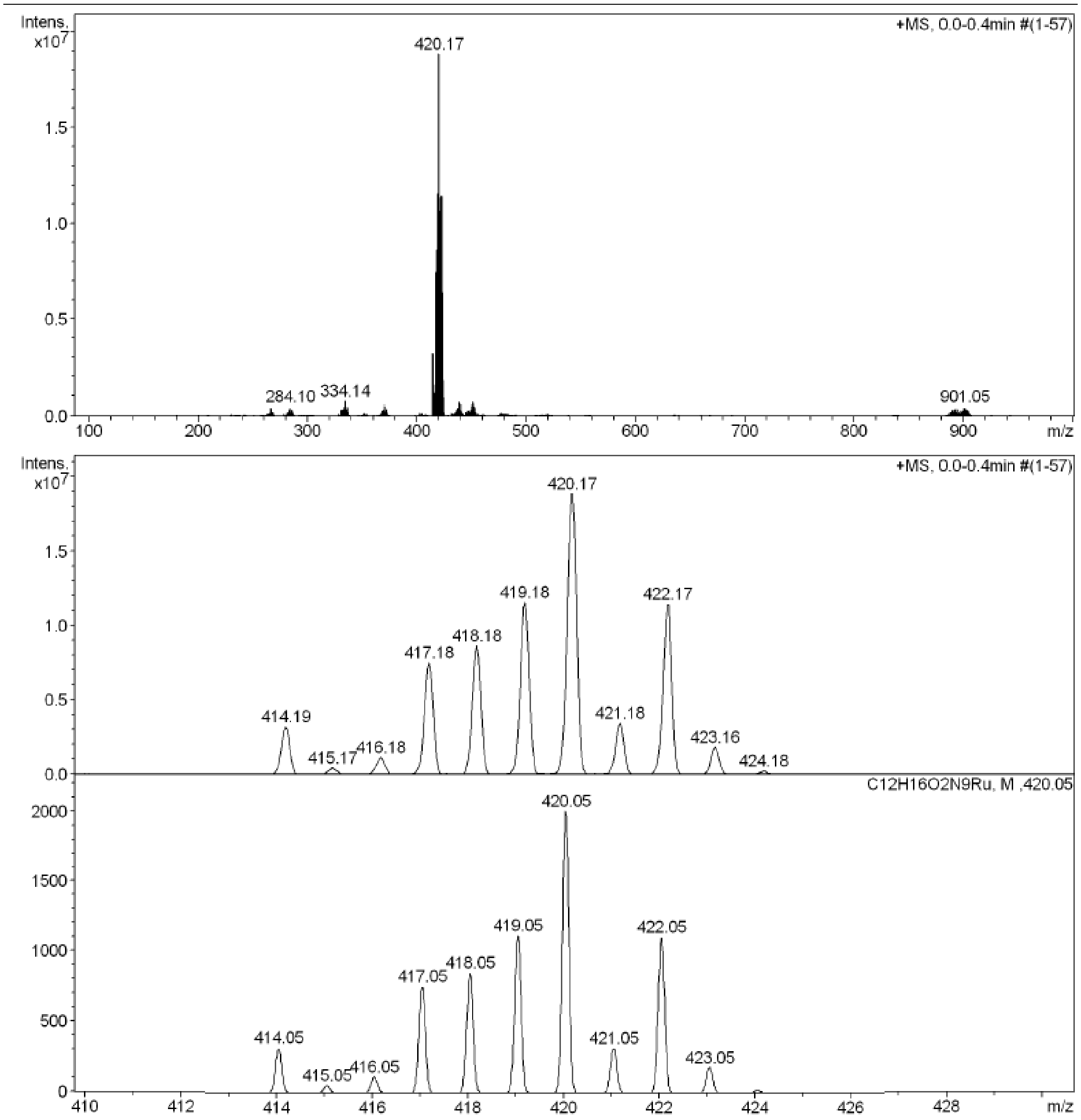

Figure S11. Positive ion ESI mass spectrum of **5** (in MeOH): full spectrum (top); experimental (middle) and calculated (bottom) isotopic distributions of the peak with *m/z* 420.17 attributed to $[Ru(OH)(NO)(pz)(Hpz)_3]^+$.

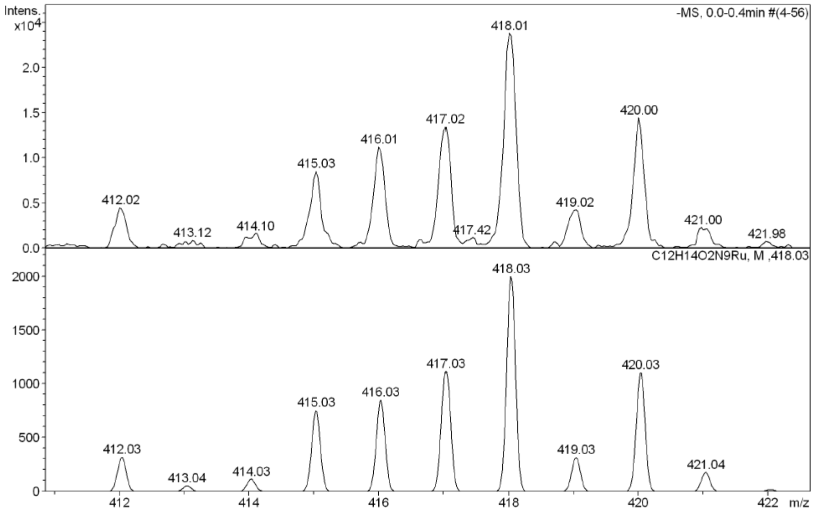

Figure S12. Negative ion ESI mass spectrum of **5** (in MeOH): experimental (top) and calculated (bottom) isotopic distributions of the peak with *m/z* 418.01 attributed to $[Ru(OH)(NO)(pz)_3(Hpz)]^-$

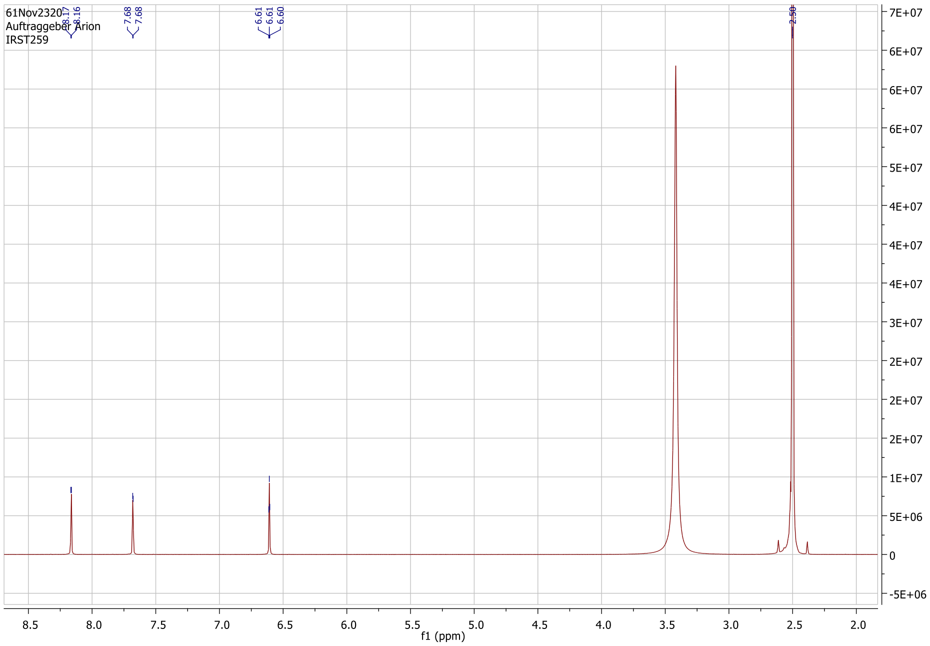

Figure S13. ^{1}H NMR (600 MHz, DMSO-d_6) spectrum of **5**.

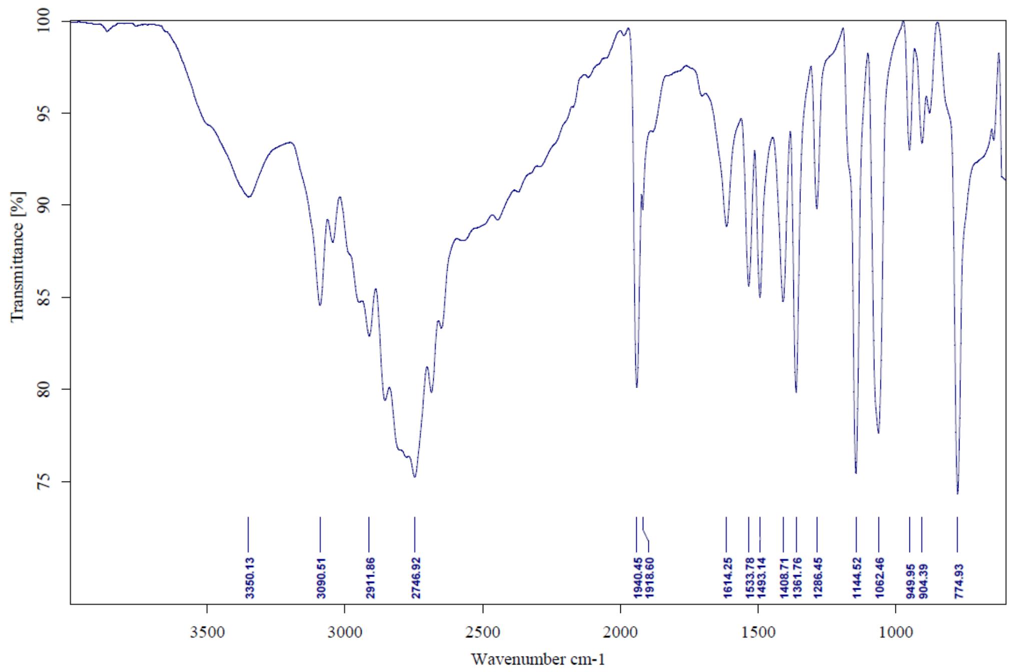

Figure S14. FTIR spectrum of crude **5**.

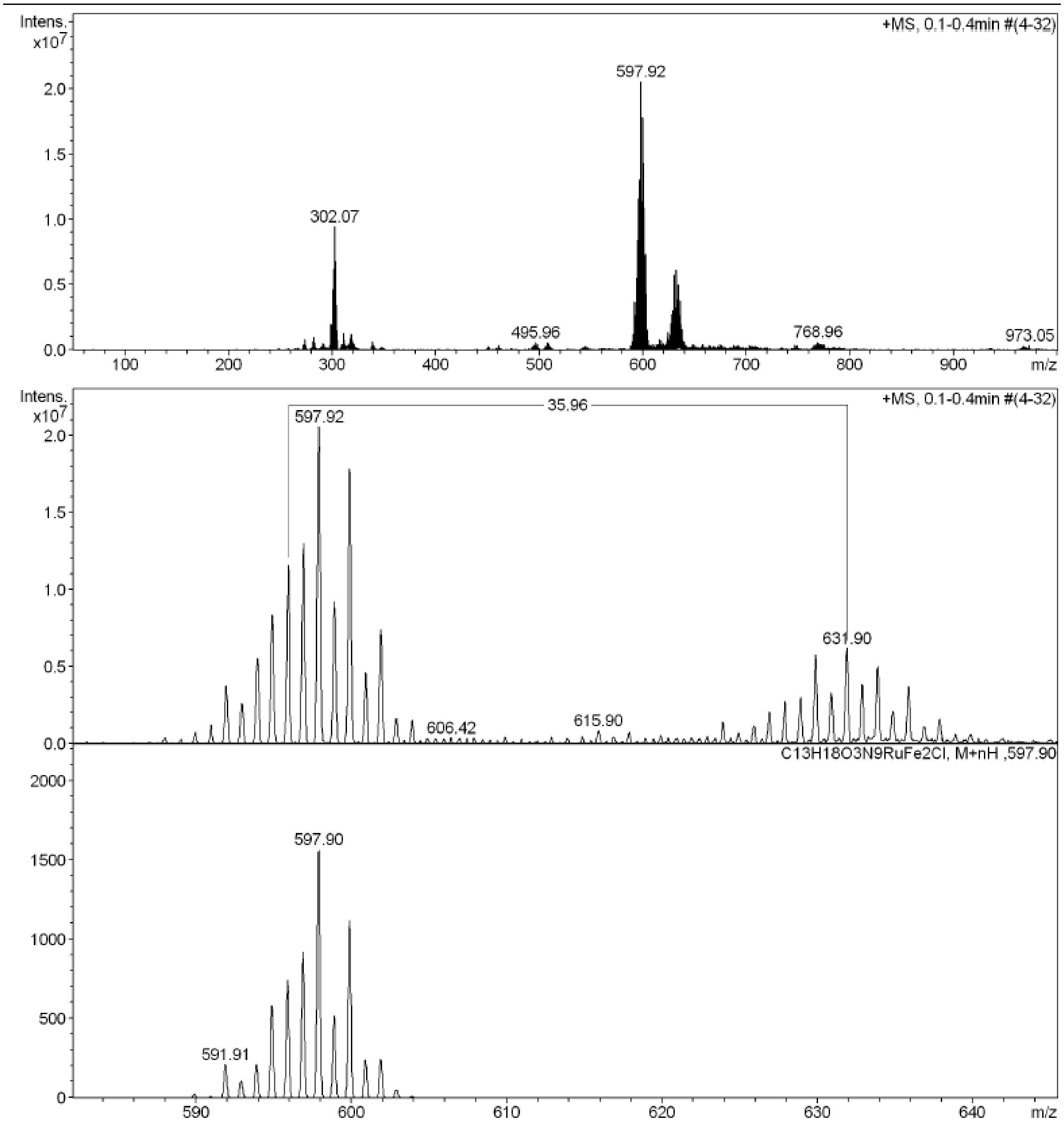

Figure S15. Positive ion ESI mass spectrum of **6** (in MeCN/MeOH+1% H_2O): full spectrum (top); experimental (middle) and calculated (bottom) isotopic distributions of the peak with *m/z* 597.92 attributed to $[M–(Hpz)–Cl]^+$.

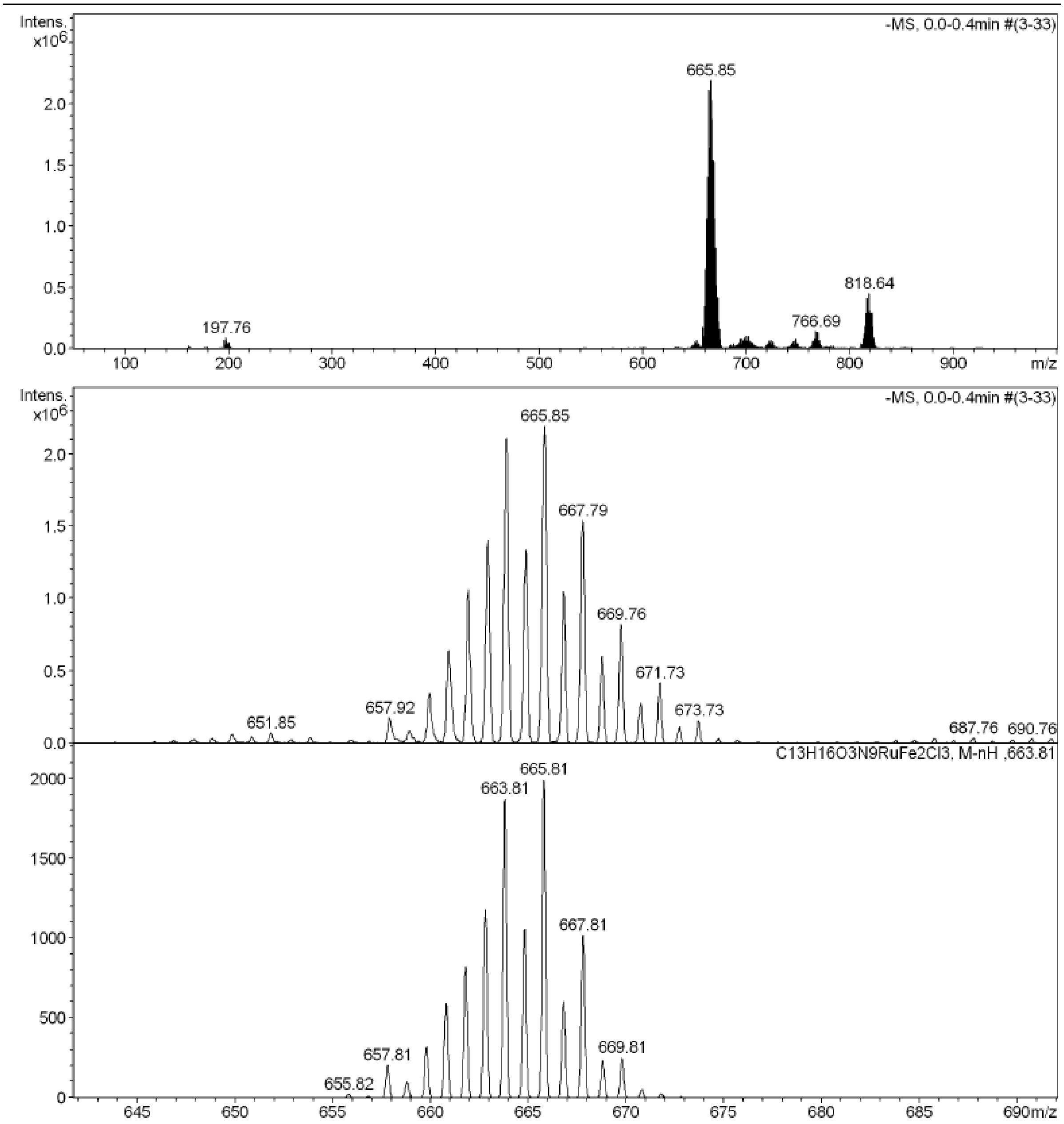

Figure S16. Negative ion ESI mass spectrum of **6** (in MeCN/MeOH+1% H_2O): full spectrum (top); experimental (middle) and calculated (bottom) isotopic distributions of the peak with *m/z* 665.85 attributed to $[M–HCl–H]^{–}$.

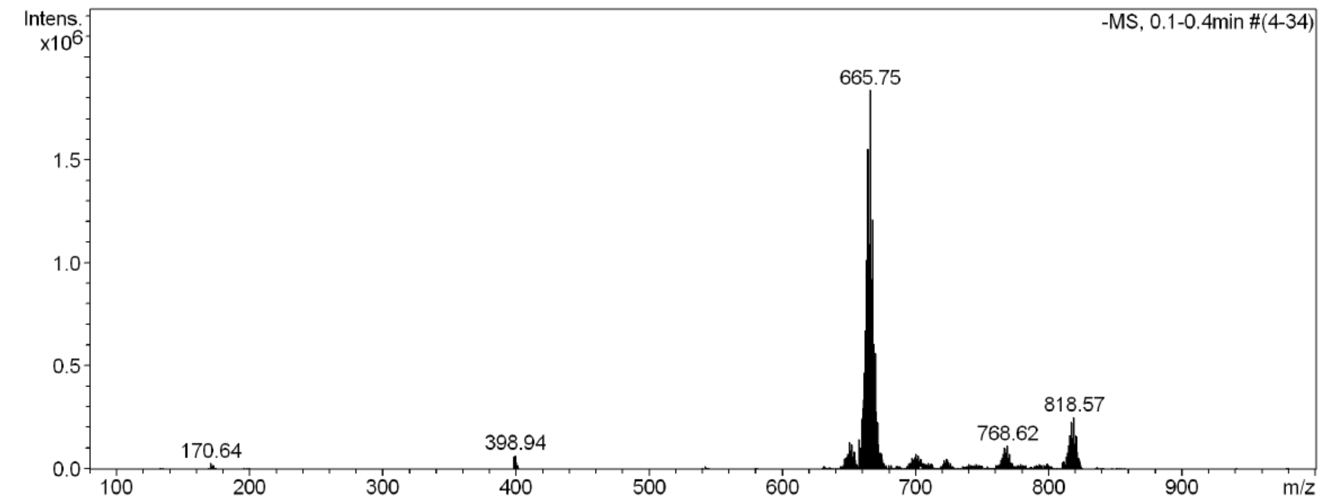

Figure S17. Negative ion ESI mass spectrum of **7** (in MeCN/MeOH+1% H_2O): the peak with *m/z* 665.75 attributed to $[M–H]^–$, where M is the unit $[Fe_2Ru^{II}Cl_3(\mu_3\text{-}O)(\mu\text{-}OMe)(\mu\text{-}pz)_3(NO)(Hpz)]$.

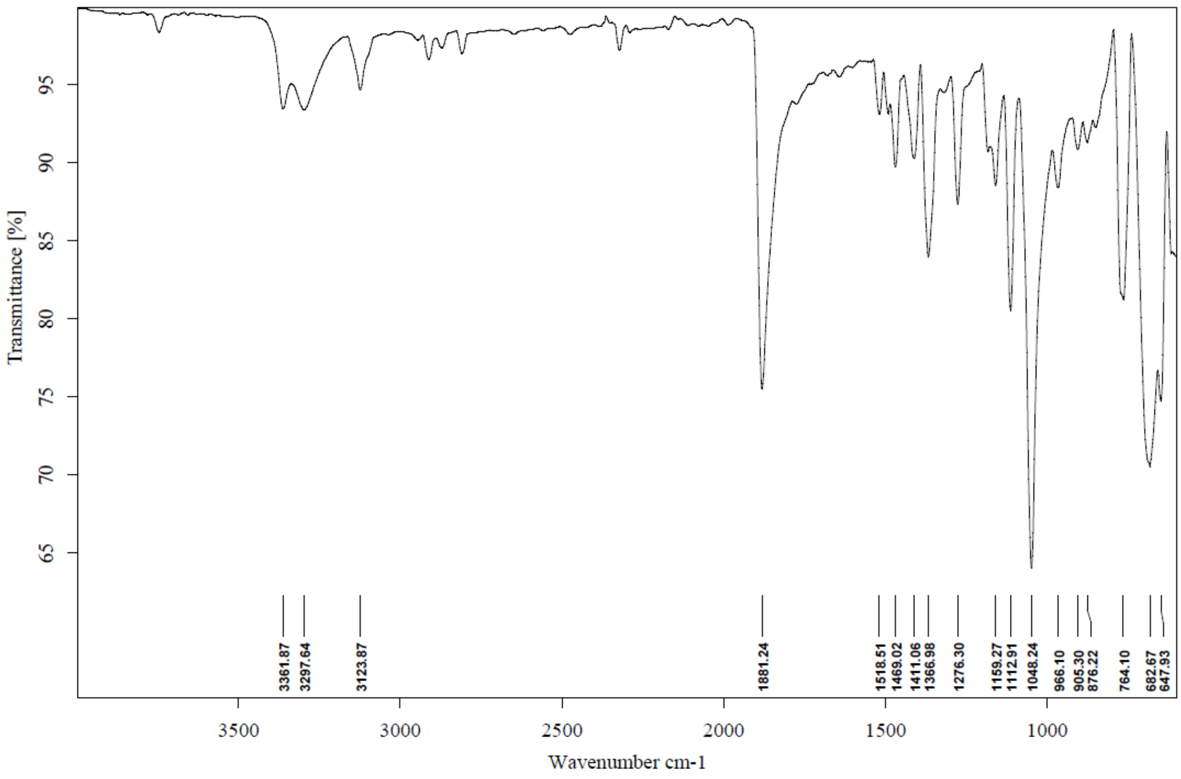

Figure S18. FTIR spectrum of **6** with $\nu_{N\text{-}H}$ and $\nu_{C\text{-}H}$ in 1*H*-pyrazole at 3362 and 3298 cm^{-1}, $\nu_{C\text{-}H}$ in OCH_3 at 3124 cm^{-1}, ν_{NO} at 1881 cm^{-1}, $\nu_{Fe\text{-}O\text{-}Fe}$ at 1113 cm^{-1}.

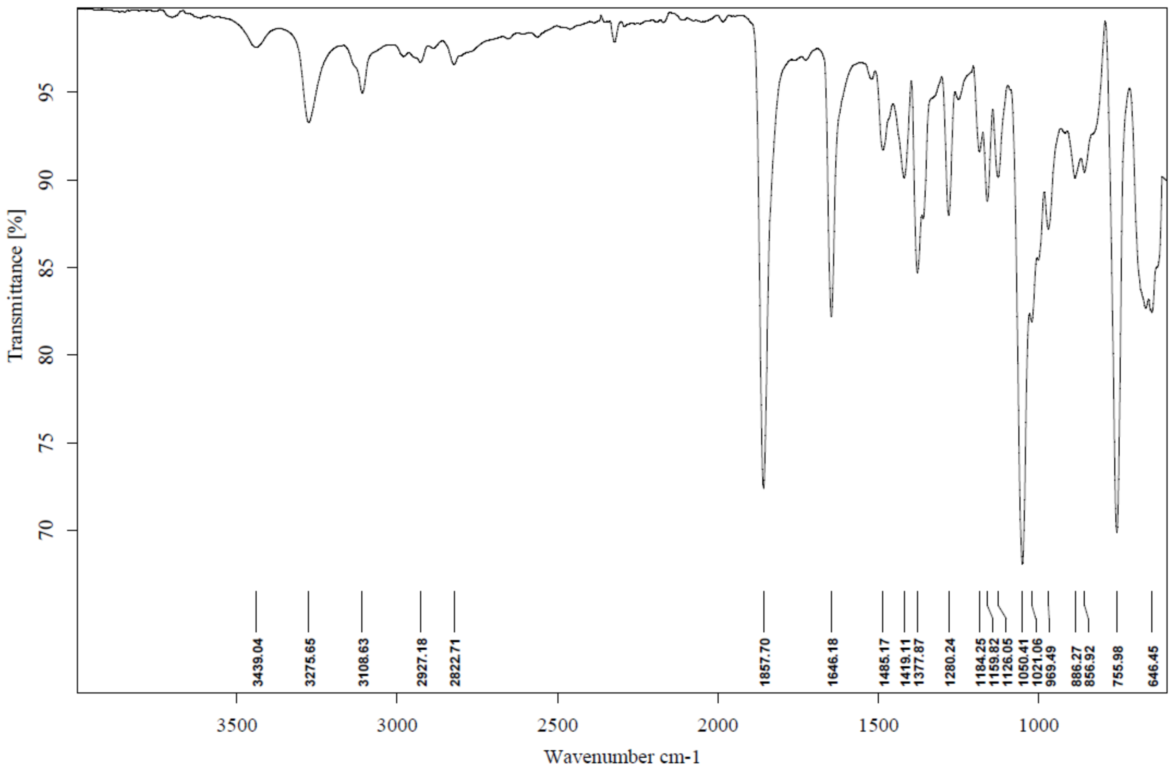

Figure S19. FTIR spectrum of **7**.

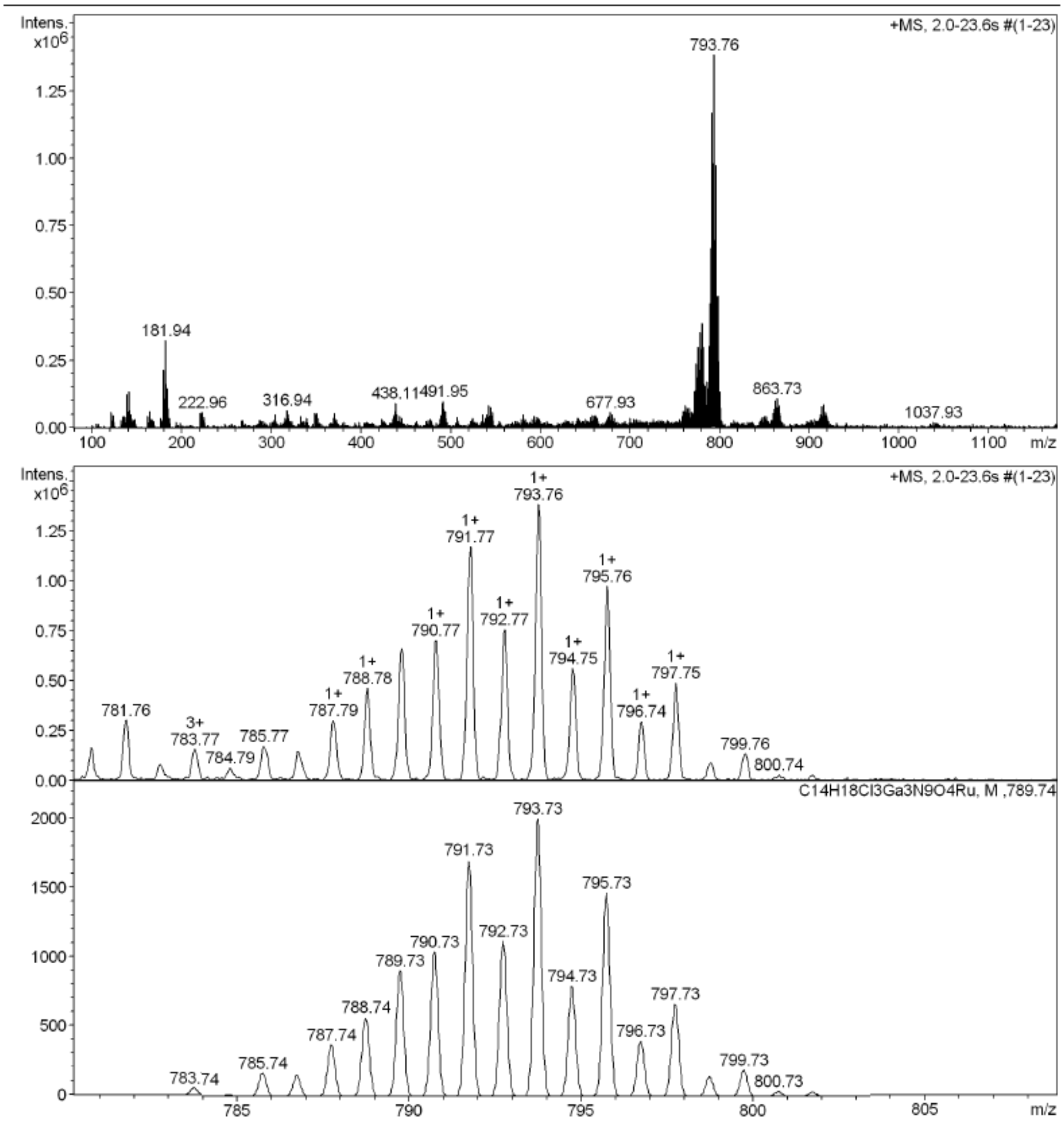

Figure S20. Positive ion ESI mass spectrum of **8** (in MeCN/MeOH+1% H_2O): full spectrum (top); experimental (middle) and calculated (bottom) isotopic distributions of the peak with *m/z* 793.76 attributed to $[M–(OMe)]^+$.

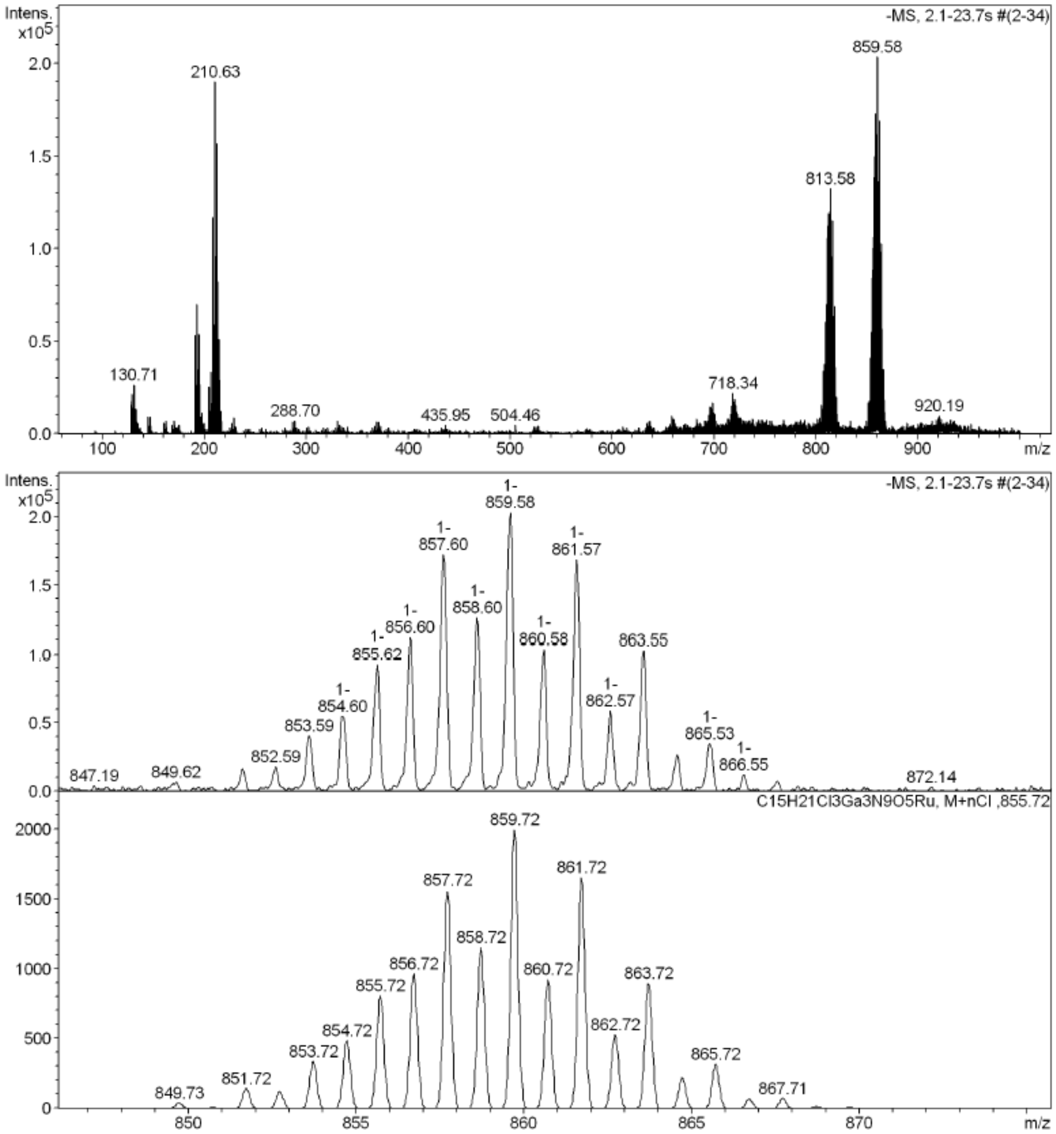

Figure S21. Negative ion ESI mass spectrum of **8** (in MeCN/MeOH+1% H_2O): full spectrum (top); experimental (middle) and calculated (bottom) isotopic distributions of the peak with *m/z* 859.58 attributed to $[M+Cl]^-$.

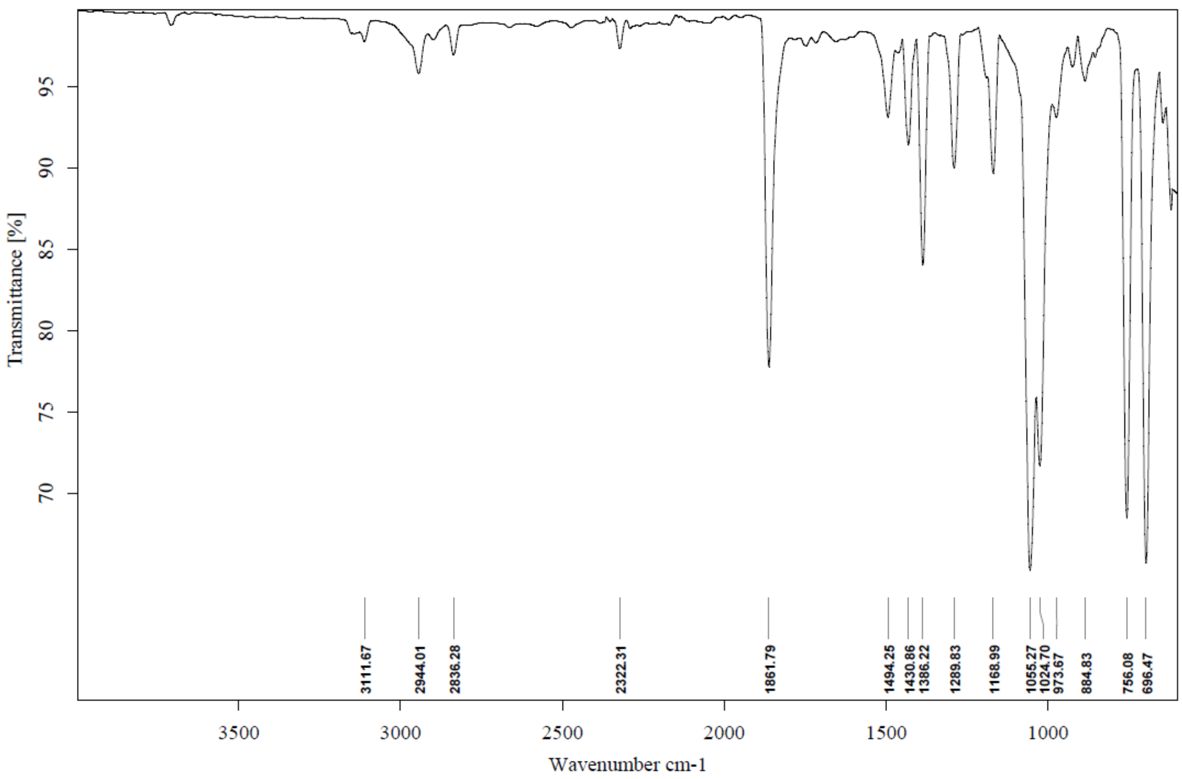

Figure S22. FTIR spectrum of **8**.

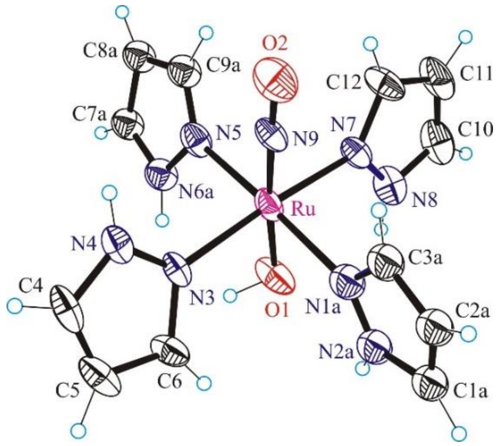

Figure S23. ORTEP view of the cation $[Ru(OH)NO(Hpz)_4]^{2+}$ in **5** with thermal ellipsoids drawn at 30% probability level. Two 1*H*-pyrazole ligands are disordered over two positions and only one component is shown. Selected bond distances (Å) and bond angles (°): Ru–O1 1.927(7), Ru–N9 1.731(7), Ru–N1a 2.075(10), Ru–N3 2.072(6), Ru–N5 2.070(8), Ru–N7 2.075(6), N9–O2 1.219(9); O1–Ru–N9 175.3(3), Ru–N9–O2 177.6(5).

Complex **5** crystallized in the monoclinic centrosymmetric space group $P2_1/n$ with one co-crystallized water molecule per asymmetric unit. The complex cation can be described as octahedral with four 1*H*-pyrazole ligands bound to Ru in equatorial positions and OH^- and NO in axial positions. Two trans pyrazole ligands were found to be disordered over two positions with site occupation factors 75:25 and 50:50, and only one position for each of them is shown in Figure S18. In the first case the most populated one is shown. The equatorial average Ru–N_{Hpz} bond length in **5** [2.073(1) Å] is significantly (4.9 σ) longer than the average Ru–N bond in two crystallographically independent molecules of $[RuCl_2(Hpz)_4]Cl$ at 2.0603(24) Å, which have only chlorido axial co-ligands rather than nitrosyl/hydroxido.[3]

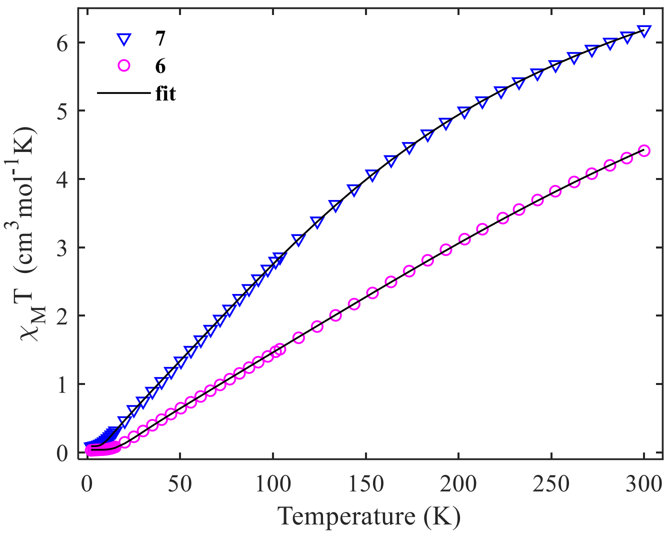

Figure S24. Temperature dependent magnetic susceptibility measurements on the polycrystalline heterotrinuclear clusters **6** and **7·MeOH**. Solid line represents the best fit to experimental data (see text).

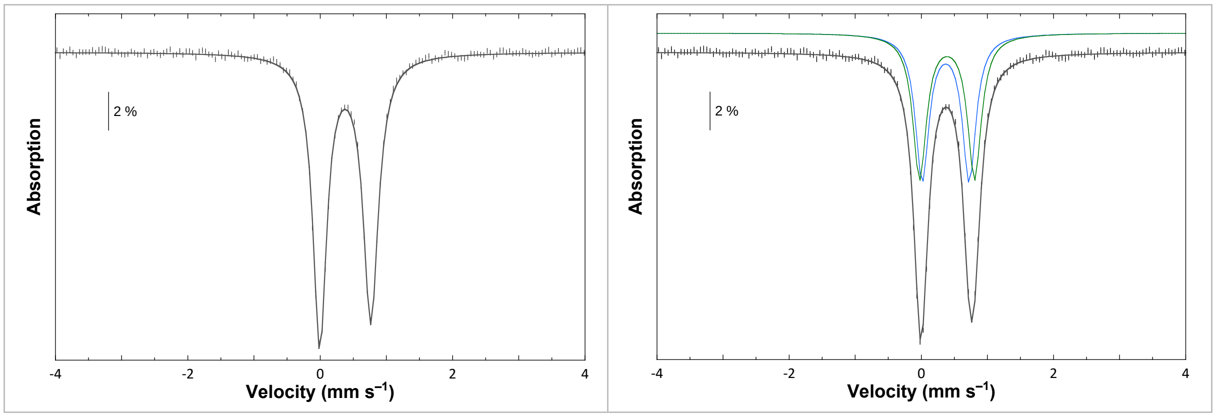

Figure S25. 82 K Mössbauer spectrum of a powder sample of **6**. A simulation assuming a unique iron site is reproduced on the left (δ = 0.38(2) mm s^{-1}, ΔE_Q = 0.77(5) mm s^{-1}) whereas a simulation assuming two nested doublets in a 1:1 ratio is shown on the right (Site 1 in green: δ_1 = 0.39(2) mm s^{-1}, ΔE_{Q1} = 0.83(5) mm s^{-1} ; Site 2 in blue: δ_2 = 0.37(2) mm s^{-1}, ΔE_{Q2} = 0.71(5) mm s^{-1}).

Figure S25 reproduces the 82 K Mössbauer spectrum recorded on a powder sample of **6** using a 0.06 T external magnetic field applied parallel to the γ-beam. A doublet is observed, the high velocity line presenting a larger linewidth and a reduced depth than the low velocity line. This asymmetry may be due to texture effects but may also evidence a slight difference between the two iron sites that are indeed not equivalent according to the X-ray structure. Simulations assuming either a unique iron site or two different iron sites in a 1:1 ratio are reproduced below. In the latter case, nested doublets were preferred versus crossed doublets, that is similar isomer shift values versus similar quadrupole splitting values. Parameters are listed in the legend to Figure S25.

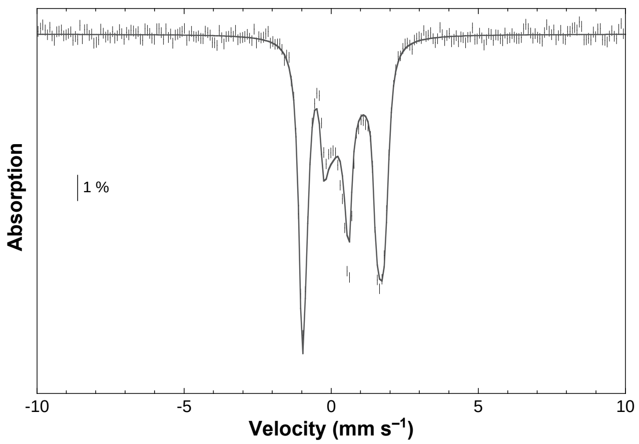

Figure S26. Experimental (hatched bars) Mössbauer spectrum recorded on a powder sample of **6** at 6 K using a 7 T external magnetic field applied parallel to the γ-beam. The overlaid solid grey line corresponds to a satisfying simulation obtained assuming a single diamagnetic iron site. The nuclear parameters are: δ = 0.39(2) mm s^{-1}, ΔE_Q = 0.79(5) mm s^{-1}, η = 0.05(5).

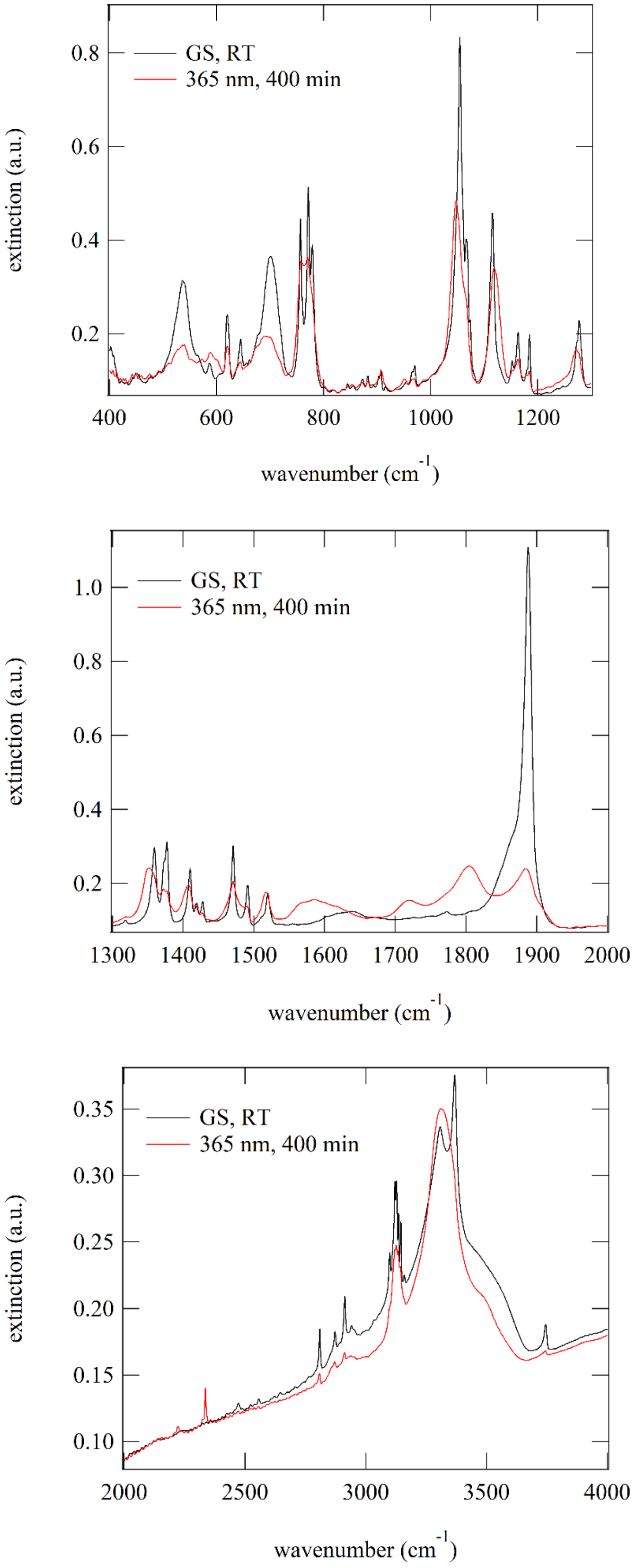

Figure S27. Full spectral range for RT irradiation of **6**: comparison of initial state and 400 min of 365 nm light irradiation with 100 mW.

^{10}[**6**]$^{+}$	^{10}[**6**]$^{-}$
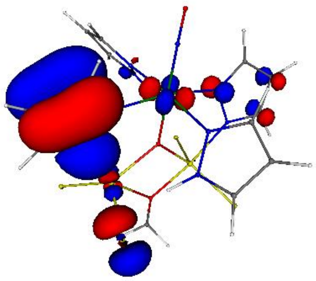<br>(b) HOMO-1 (–0.375)	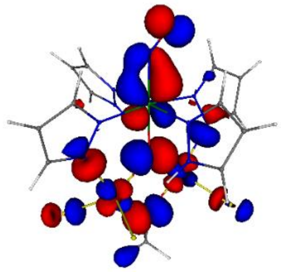<br>(c) HOMO-1 (–0.120)
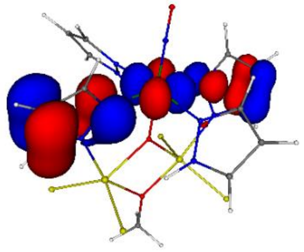<br>(e) HOMO (–0.361)	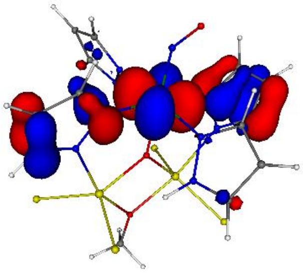<br>(f) HOMO (–0.106)
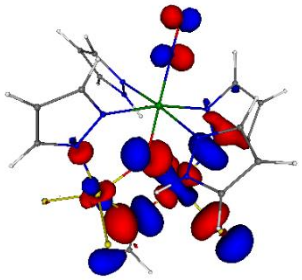<br>(h) LUMO (–0.304)	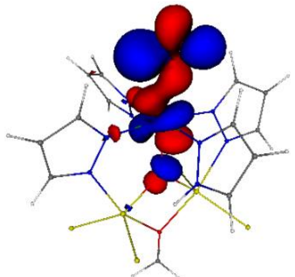<br>(i) LUMO (0.027)
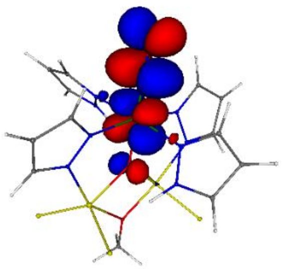<br>(k) LUMO+1 (–0.243)	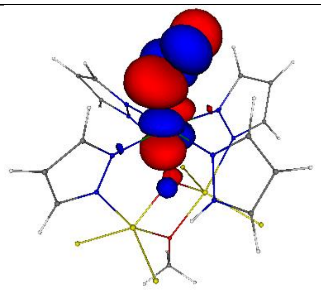<br>(l) LUMO+1 (0.038)
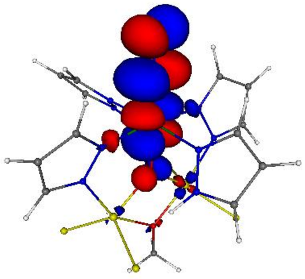<br>(n) LUMO+2 (–0.240)	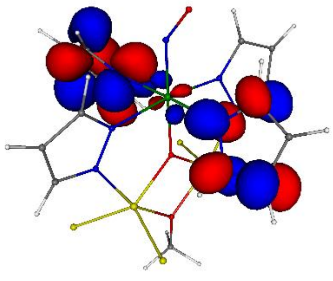<br>(o) LUMO+2 (0.081)

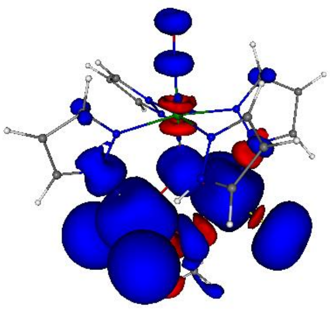	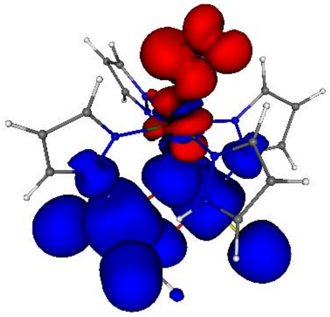
spin density	spin density

Figure S28. Frontier α orbitals and spin densities of ^{10}[**6**]$^{+}$ and ^{10}[**6**]$^{-}$ (eigenvalues in hartrees are shown in parentheses), frontier orbitals and spin density isosurface value is 0.04 e bohr^{-3} and 0.002 e bohr^{-3}.

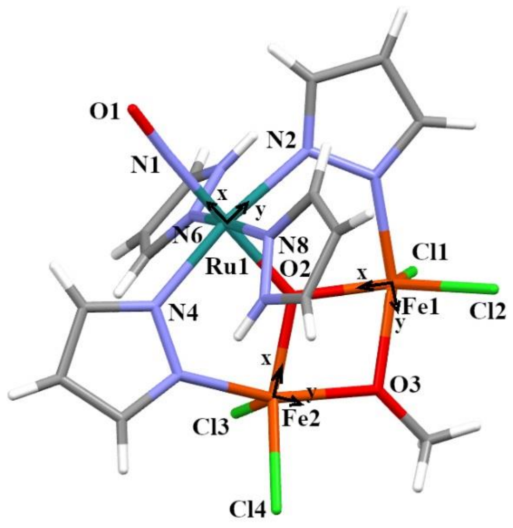

Figure S29. Result of synchrotron X-ray diffraction study of **6**.

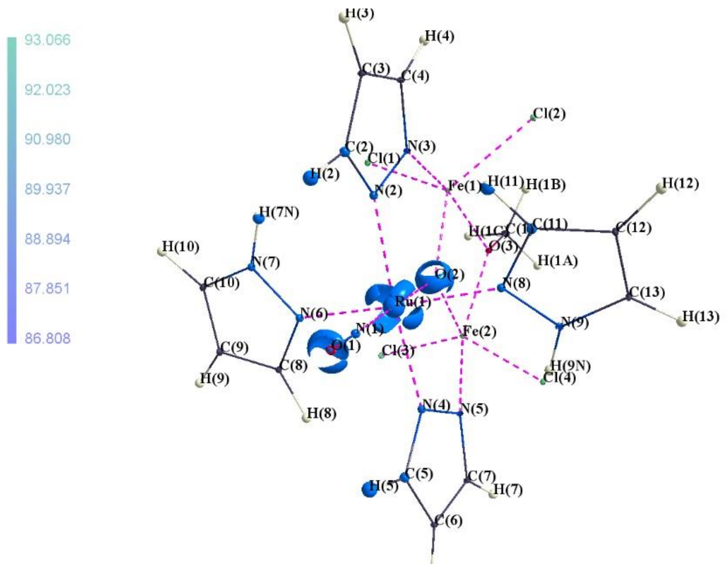

Figure S30. 3D plot of the Laplacian of electron density of **6** at the isosurface value of 90 e/Å^5, generated using the method of Hübschle and Dittrich.[4]

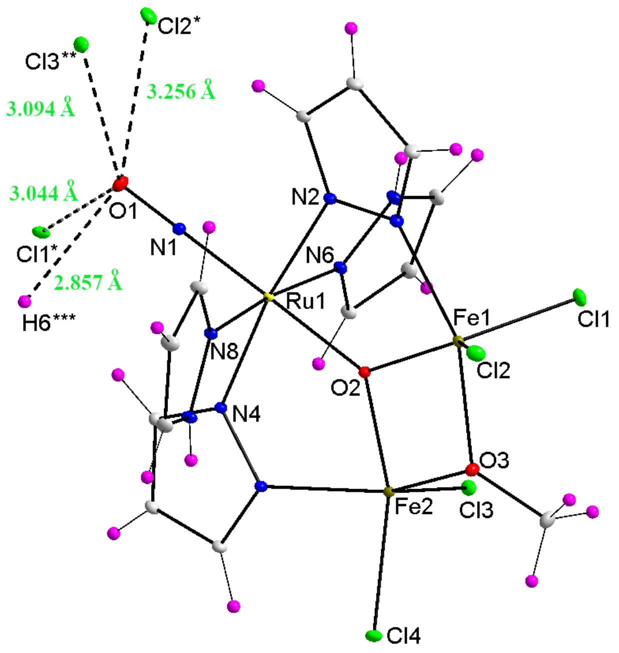

Figure S31. ORTEP plots of the asymmetric units of **6**. Displacement ellipsoids are at 30% probability level. Symmetry code used: *) $x - ½$, $-y + 3/2$, $z - ½$; **) $x - 1$, y, z; ***) $1 - x$, $1 - y$, $1 - z$.

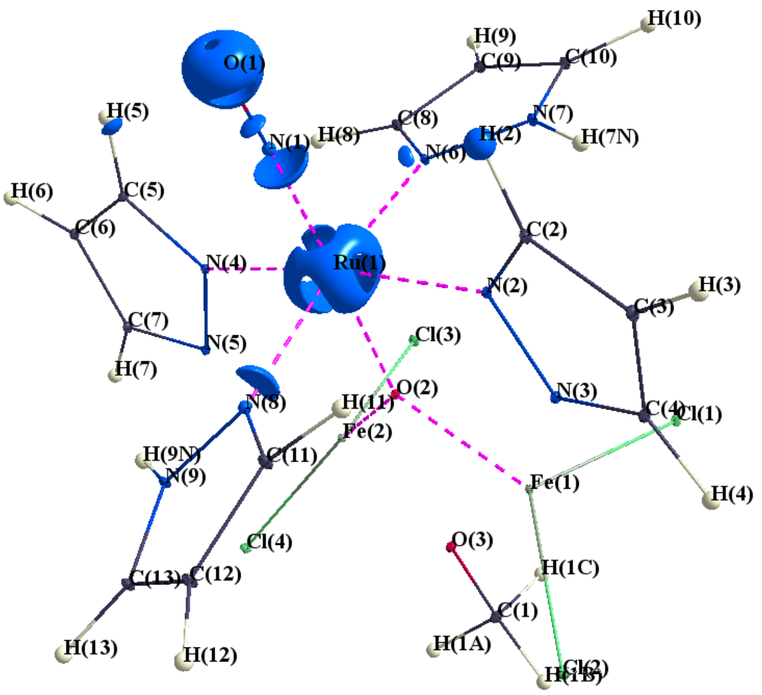

Figure S32. 3D plot of the Laplacian of electron density of **6** around N1 at the isosurface value of 55 e/Å^5, generated using the method of Hübschle and Dittrich.4

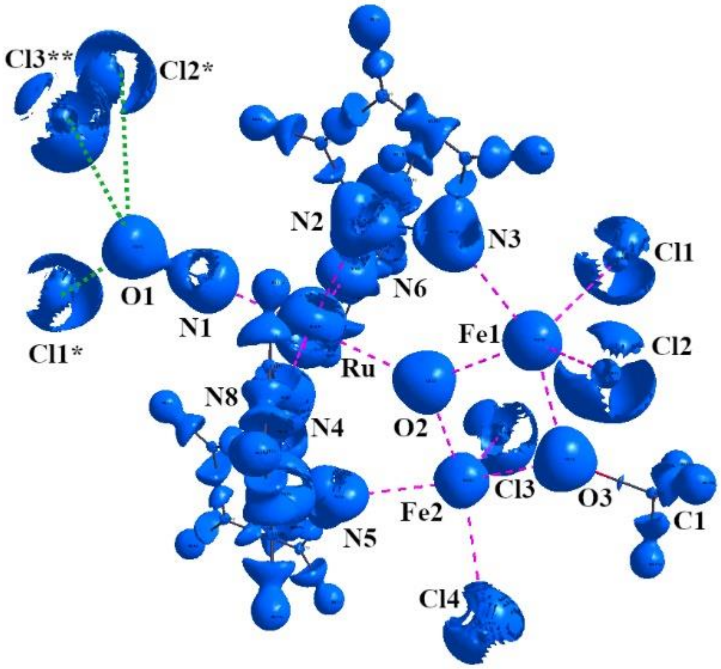

Figure S33. 3D plot of the Laplacian of electron density of **6** at the isosurface value of 16 e/Å^5, generated using the method of Hübschle and Dittrich.4 Symmetry code used: *) *x* – ½, –*y* + 3/2, z – ½; **) *x* – 1, *y*, *z*.

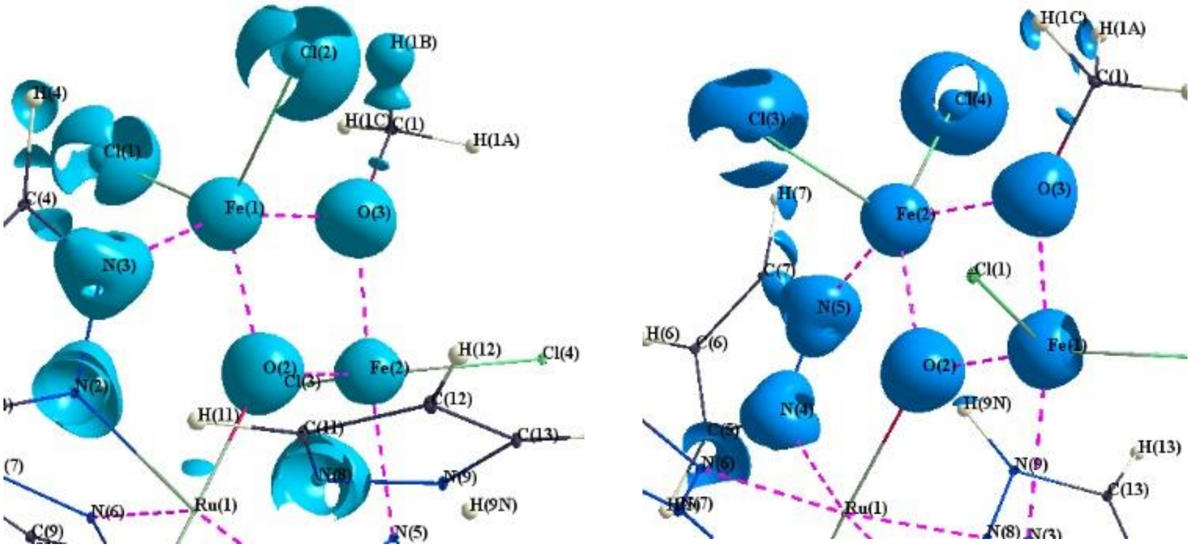

Figure S34. 3D plot of the Laplacian of electron density of **6** around Fe1/Fe2 at the isosurface value of 15.5 e/Å^5, generated using the method of Hübschle and Dittrich.4

Table S1. B3LYP, BLYP, CAS and NEVPT2 (def2-SVP basis set) total energies of spin states of the studied complex **6**.

	E [A.U.]		*E* [A.U.]	
	**B3LYP**	**BLYP**	**CAS**	**NEVPT2**
^{13}[**6**]^{0}	-5685.822745	-5685.424973	-	-
^{11}[**6**]^{0}	-5685.861239	-5685.470459	-5672.178283	-5677.614084
^{9}[**6**]^{0}	-5685.847161	-5685.469899	-5672.178410	-5677.614367
^{7}[**6**]^{0}	-5685.824403	-5685.458283	-5672.178512	-5677.614609
^{5}[**6**]^{0}	-5685.802197	-5685.442637	-5672.178589	-5677.614797
^{3}[**6**]^{0}	-5685.778531	-5685.444659	-5672.178640	-5677.614925
^{1}[**6**]^{0}	-5685.863162	-5685.474930	-5672.178666	-5677.614991
^{12}[**6**]$^{+}$	-5685.575507	-5685.211615		
^{10}[**6**]$^{+}$	-5685.582676	-5685.236759	-5677.288178	-5671.703385
^{8}[**6**]$^{+}$	-5685.559589	-5685.226782	-5677.287438	-5671.703223
^{6}[**6**]$^{+}$	-5685.539953	-5685.217689	-5677.286883	-5671.703096
^{4}[**6**]$^{+}$	-5685.523468	-5685.223321	-5677.286498	-5671.703006
^{2}[**6**]$^{+}$	-5685.580078	-5685.234447	-5677.286269	-5671.702951
^{12}[**6**]$^{-}$	-5685.961987	-5685.548350	-5672.30203	-5677.607176
^{10}[**6**]$^{-}$	-5685.964485	-5685.560739	-5672.302642	-5677.608584
^{8}[**6**]$^{-}$	-5685.941839	-5685.560211	-5672.302817	-5677.60892
^{6}[**6**]$^{-}$	-5685.932084	-5685.545313	-5672.302953	-5677.609199
^{4}[**6**]$^{-}$	-5685.873597	-5685.552910	-5672.30305	-5677.609406
^{2}[**6**]$^{-}$	-5685.967289	-5685.561026	-5672.303108	-5677.609533

Table S2. Relative B3LYP and BLYP def2-SVP energies ($\Delta E = E_S$), $\langle S^2 \rangle$ expectation values, *J* coupling of spin states, relative CASSCF and NEVPT2 def2-SVP energies of the studied species of [**6**]$^{+}$ and [**6**]$^{-}$, note that *J* coupling is given with respect to eqs. (2) and (3)

	Δ*E* [kJ/mol]		S^2 [A.U.]		*J* [kJ/mol]		Δ*E* [kJ/mol]	
	**B3LYP**	**BLYP**	**B3LYP**	**BLYP**	**B3LYP**	**BLYP**	**CASSCF**	**NEVPT2**
^{12}[**6**]$^{+}$	18.8	66.0	35.778	35.776	-[a]	-[a]		
^{10}[**6**]$^{+}$	0.0	0.0	24.890	24.828	-	-	0.0	0.0
^{8}[**6**]$^{+}$	60.6	26.2	16.855	15.824	7.544	2.909	0.8	1.9
^{6}[**6**]$^{+}$	112.1	50.1	9.070	8.857	7.090	3.135	1.4	3.4
^{4}[**6**]$^{+}$	155.4	35.3	4.989	5.854	7.811	1.859	1.8	4.4
^{2}[**6**]$^{+}$	6.8	6.1	4.791	4.639	0.339	0.301	2.1	5.0
^{12}[**6**]$^{-}$	6.6	32.5	35.7869	35.7669	-	-	0.0	0.0
^{10}[**6**]$^{-}$	0.0	0.0	25.6969	24.7919	-0.650	-2.964	-1.6	-3.7
^{8}[**6**]$^{-}$	59.5	1.4	15.8291	16.3524	2.650	-1.604	-2.1	-4.6
^{6}[**6**]$^{-}$	85.1	40.5	9.8117	9.6202	3.022	0.305	-2.4	-5.3
^{4}[**6**]$^{-}$	238.6	20.6	5.3669	6.5506	7.629	-0.410	-2.7	-5.8
^{2}[**6**]$^{-}$	-7.4	-0.8	5.6348	5.1916	-0.462	-1.088	-2.8	-6.2

[a] not relevant

Table S3. Selected B3LYP/def2-SVP bond distances (Å) and angles (°) of chosen [**6**] species

Distances	^{11}[**6**]^{0}	^{1}[**6**]^{0}	^{12}[**6**]$^{+}$	^{10}[**6**]$^{+}$	^{2}[**6**]$^{+}$	^{12}[**6**]$^{-}$	^{10}[**6**]$^{-}$	^{2}[**6**]$^{-}$
**Ru-N9**	1.750	1.750	1.776	1.748	1.747	1.853	1.843	1.848
**Ru-N5**	2.090	2.088	2.037	2.081	2.082	2.094	2.095	2.101
**Ru-N3**	2.089	2.090	2.037	2.090	2.092	2.101	2.101	2.092
**Ru-N1**	2.105	2.106	2.104	2.106	2.105	2.110	2.115	2.101
**Ru-N7**	2.107	2.107	2.107	2.107	2.107	2.099	2.098	2.112
**Ru-O1**	1.913	1.917	1.895	1.945	1.950	1.940	2.027	2.032
**N9-O2**	1.154	1.154	1.144	1.144	1.144	1.191	1.190	1.190
**O3-C13**	1.411	1.413	1.421	1.418	1.417	1.399	1.399	1.400
**Fe1-Fe2**	3.129	3.159	3.130	3.110	3.111	3.060	3.055	3.090
**O3-Fe1**	1.976	1.973	1.960	2.096	2.174	2.000	1.999	1.995
**O3-Fe2**	1.983	1.981	1.966	1.855	1.815	2.001	1.998	2.011
**O1-Fe1**	2.017	2.021	2.038	2.011	2.004	1.935	1.935	1.942
**O1-Fe2**	2.007	2.011	2.049	1.905	1.886	1.940	1.943	1.925
**Angles**	^{11}[**6**]^{0}	^{1}[**6**]^{0}	^{12}[**6**]$^{+}$	^{10}[**6**]$^{+}$	^{2}[**6**]$^{+}$	^{12}[**6**]$^{-}$	^{10}[**6**]$^{-}$	^{2}[**6**]$^{-}$
**Ru-N9-O2**	179.870	179.900	179.640	178.780	178.540	144.550	144.930	144.400
**Fe1O3C13**	128.830	128.14	127.730	129.840	130.09	131.760	131.74	131.08
**Fe2O3C13**	126.630	125.7	126.290	126.370	126.76	128.360	128.35	127.98
**O1Fe1O3**	76.720	75.31	77.020	71.660	70.39	77.990	78.090	76.43
**O1Fe2O3**	76.650	75.37	77.160	79.510	81.3	77.910	78.260	76.45

Table S4a. B3LYP/def2-SVP MPA charge and spin of neutral [**6**]^0 and oxidized [**6**]$^+$ species for the chosen spin states.

	^{11}[**6**]^0		^{1}[**6**]^0		^{12}[**6**]$^+$		^{10}[**6**]$^+$		^{2}[**6**]$^+$	
	**charge**	**spin**	**charge**	**spin**	**charge**	**spin**	**charge**	**spin**	**charge**	**spin**
**Ru**	0.482	-0.047	0.684	0.001	0.717	0.428	0.719	-0.040	0.720	0.008
**Fe1**	0.662	3.966	0.802	-4.143	0.787	4.124	0.698	3.531	0.685	-3.317
**Fe2**	0.668	3.966	0.807	4.143	0.792	4.124	0.770	4.085	0.777	4.123
**Cl11**	-0.300	0.248	-0.345	-0.203	-0.300	0.230	-0.233	0.262	-0.227	-0.249
**Cl12**	-0.265	0.334	-0.316	-0.270	-0.260	0.311	-0.152	0.223	-0.144	-0.198
**Cl13**	-0.267	0.329	-0.318	0.265	-0.262	0.307	-0.225	0.314	-0.233	0.321
**Cl14**	-0.297	0.253	-0.343	0.208	-0.298	0.234	-0.268	0.264	-0.272	0.255
**NO**	-0.009	0.192	0.018	-0.003	0.111	0.002	0.104	0.058	0.105	-0.004
**O1**	-0.739	0.205	-0.721	-0.006	-0.722	0.162	-0.710	0.163	-0.717	-0.036
**OCH3**	-0.238	0.355	-0.306	0.004	-0.722	0.162	-0.710	0.163	-0.717	-0.036
**Lig1**	-0.191	0.110	-0.248	-0.085	-0.596	0.296	-0.507	0.020	-0.503	-0.039
**Lig2**	-0.192	0.111	-0.248	0.086	-0.265	0.329	-0.133	0.024	-0.131	-0.042
**Lig3**	0.280	-0.001	0.272	-0.004	-0.001	0.362	-0.067	0.006	-0.055	0.018
**Lig4**	0.281	-0.001	0.273	0.004	-0.002	0.361	-0.151	0.114	-0.160	0.124

Table S4b. B3LYP/def2-SVP MPA charge and spin of the reduced [**6**]$^–$ complex for the chosen spin states.

	^{12}[**6**]$^–$		^{10}[**6**]$^–$		^{2}[**6**]$^–$	
	**charge**	**spin**	**charge**	**spin**	**charge**	**spin**
**Ru**	0.498	0.153	0.505	-0.184	0.503	0.166
**Fe1**	0.823	4.165	0.825	4.161	0.818	-4.140
**Fe2**	0.830	4.163	0.826	4.151	0.827	4.154
**Cl11**	-0.396	0.179	-0.398	0.175	-0.404	-0.166
**Cl12**	-0.384	0.222	-0.385	0.221	-0.391	-0.211
**Cl13**	-0.389	0.214	-0.393	0.211	-0.390	0.210
**Cl14**	-0.395	0.183	-0.399	0.179	-0.396	0.177
**NO**	-0.168	0.844	-0.164	-0.702	-0.162	0.762
**O1**	-0.719	0.413	-0.718	0.361	-0.710	0.011
**OCH3**	-0.719	0.413	-0.589	0.249	-0.710	0.011
**Lig1**	-0.587	0.252	-0.361	0.278	-0.584	0.012
**Lig2**	-0.360	0.282	-0.343	0.090	-0.360	0.015
**Lig3**	-0.344	0.091	-0.345	0.081	-0.345	-0.074
**Lig4**	-0.342	0.087	0.176	-0.006	-0.343	0.082

Table S5. B3LYP/def2-SVP Mulliken d-orbital and s-orbital populations of various neutral and charged species of **6**.

Species	atom	spin	d_{z2}	d_{xz}	d_{yz}	d_{x2-y2}	d_{xy}	s	d_{total}
^{11}[**6**]^{0}	**Ru**	α	0.376	0.679	0.693	0.423	0.974	0.170[a]	3.145
		β	0.365	0.730	0.726	0.425	0.975	0.176[a]	3.221
	**Fe1**	α	1.000	1.008	1.001	0.998	1.002	0.272	5.009
		β	0.141	0.233	0.165	0.176	0.212	0.247	0.927
	**Fe2**	α	1.000	1.008	1.001	0.998	1.002	0.272	5.010
		β	0.142	0.233	0.160	0.179	0.212	0.247	0.926
^{1}[**6**]^{0}	**Ru**	α	0.364	0.698	0.713	0.421	0.979	0.167[a]	3.176
		β	0.364	0.699	0.714	0.421	0.979	0.168[a]	3.178
	**Fe1**	α	0.996	1.009	0.999	0.995	1.001	0.271	5.002
		β	0.143	0.270	0.165	0.185	0.200	0.235	0.963
	**Fe2**	α	0.147	0.269	0.156	0.182	0.210	0.237	0.964
		β	0.997	1.009	0.999	0.995	1.001	0.272	5.001
^{12}[**6**]$^{+}$	**Ru**	α	0.423	0.743	0.746	0.464	0.983	0.178	3.358
		β	0.394	0.738	0.713	0.458	0.629	0.176	2.931
	**Fe1**	α	1.000	1.006	1.001	0.998	1.002	0.268	5.007
		β	0.152	0.222	0.162	0.185	0.227	0.243	0.949
	**Fe2**	α	1.000	1.007	0.999	0.999	1.002	0.268	5.007
		β	0.139	0.219	0.165	0.198	0.228	0.243	0.949
^{10}[**6**]$^{+}$	**Ru**	α	0.363	0.703	0.706	0.424	0.980	0.165	3.176
		β	0.351	0.720	0.727	0.424	0.980	0.170	3.202
	**Fe1**	α	0.964	0.791	0.990	0.982	1.003	0.274	4.730
		β	0.205	0.318	0.244	0.230	0.286	0.241	1.282
	**Fe2**	α	0.998	0.988	1.000	0.997	1.002	0.272	4.984
		β	0.166	0.218	0.197	0.203	0.196	0.240	0.980
^{2}[**6**]$^{+}$	**Ru**	α	0.357	0.711	0.720	0.425	0.980	0.168	3.193
		β	0.354	0.712	0.714	0.423	0.980	0.168	3.185
	**Fe1**	α	0.226	0.353	0.259	0.234	0.321	0.271	1.393
		β	0.939	0.734	0.988	0.974	1.002	0.242	4.638
	**Fe2**	α	0.998	1.006	1.000	0.998	1.000	0.242	5.003
		β	0.163	0.202	0.200	0.199	0.194	0.271	0.957
^{12}[**6**]$^{-}$	**Ru**	α	0.403	0.764	0.913	0.357	0.953	0.168	3.390
		β	0.341	0.785	0.805	0.352	0.951	0.170	3.234
	**Fe1**	α	1.000	1.011	1.001	0.997	1.003	0.271	5.012
		β	0.127	0.259	0.161	0.173	0.192	0.247	0.912
	**Fe2**	α	1.000	1.011	1.001	0.997	1.003	0.271	5.013
		β	0.132	0.258	0.155	0.174	0.194	0.247	0.913
^{10}[**6**]$^{-}$	**Ru**	α	0.356	0.763	0.793	0.355	0.953	0.168	3.220
		β	0.405	0.801	0.877	0.359	0.954	0.171	3.395
	**Fe1**	α	1.000	1.011	1.001	0.997	1.003	0.271	5.012
		β	0.135	0.254	0.159	0.174	0.193	0.246	0.915
	**Fe2**	α	1.000	1.010	1.001	0.997	1.003	0.271	5.011
		β	0.127	0.267	0.157	0.181	0.191	0.246	0.923
^{2}[**6**]$^{-}$	**Ru**	α	0.406	0.824	0.850	0.358	0.955	0.169	3.392
		β	0.355	0.778	0.789	0.354	0.953	0.169	3.228
	**Fe1**	α	0.124	0.278	0.155	0.181	0.194	0.273	0.933
		β	0.999	1.008	1.000	0.996	1.002	0.244	5.005
	**Fe2**	α	1.000	1.009	1.000	0.997	1.002	0.244	5.008
		β	0.129	0.272	0.148	0.173	0.198	0.272	0.920

[a] Note that the p-orbital population is close to 0.3 in value.

Table S6. B3LYP/TZVP and CASSCF/def2-SVP Mulliken d-orbital populations of various neutral and charged species of **6**.

B3LYP	atom	spin	d_{z2}	d_{xz}	d_{yz}	d_{x2-y2}	d_{xy}	d_{total}
^{11}[**6**]^{0}	**Fe1**	α	1.007	1.016	1.007	1.008	1.010	5.049
		β	0.146	0.245	0.176	0.185	0.221	0.974
	**Fe2**	α	1.007	1.017	1.007	1.008	1.010	5.049
		β	0.148	0.244	0.171	0.188	0.222	0.972
^{1}[**6**]^{0}	**Fe1**	α	1.004	1.017	1.006	1.005	1.009	5.041
		β	0.148	0.283	0.177	0.194	0.211	1.013
	**Fe2**	α	0.152	0.282	0.167	0.191	0.221	1.013
		β	1.004	1.016	1.005	1.005	1.009	5.040
^{12}[**6**]$^{+}$	**Fe1**	α	1.007	1.014	1.006	1.009	1.010	5.047
		β	0.152	0.234	0.171	0.201	0.237	0.995
	**Fe2**	α	1.007	1.015	1.006	1.009	1.010	5.047
		β	0.153	0.233	0.167	0.205	0.236	0.994
^{10}[**6**]$^{+}$	**Fe1**	α	0.973	0.803	0.998	0.993	1.012	4.779
		β	0.210	0.328	0.255	0.239	0.293	1.326
	**Fe2**	α	1.005	0.996	1.007	1.007	1.009	5.024
		β	0.171	0.230	0.208	0.212	0.205	1.025
^{2}[**6**]$^{+}$	**Fe1**	α	1.005	1.013	1.007	1.008	1.008	5.042
		β	0.167	0.213	0.210	0.208	0.203	1.001
	**Fe2**	α	0.231	0.362	0.270	0.244	0.327	1.434
		β	0.949	0.746	0.996	0.986	1.011	4.688
^{12}[**6**]$^{–}$	**Fe1**	α	1.007	1.020	1.007	1.008	1.010	5.051
		β	0.133	0.271	0.171	0.182	0.202	0.959
	**Fe2**	α	1.007	1.019	1.007	1.008	1.010	5.051
		β	0.137	0.270	0.166	0.183	0.204	0.960
^{10}[**6**]$^{–}$	**Fe1**	α	1.007	1.019	1.007	1.007	1.010	5.051
		β	0.142	0.263	0.171	0.180	0.207	0.963
	**Fe2**	α	1.006	1.018	1.007	1.007	1.010	5.049
		β	0.133	0.278	0.169	0.190	0.201	0.971
^{2}[**6**]$^{–}$	**Fe1**	α	1.006	1.018	1.006	1.007	1.010	5.046
		β	0.135	0.283	0.159	0.182	0.209	0.969
	**Fe2**	α	0.131	0.290	0.166	0.192	0.204	0.984
		β	1.006	1.016	1.006	1.006	1.009	5.044
**CASSCF**	**atom**	**spin**	d_{z2}	d_{xz}	d_{yz}	d_{x2-y2}	d_{xy}	d_{total}
^{2}[**6**]$^{–}$	**Fe1**		1.0486	1.1576	1.0560	1.0741	1.0704	5.4067
	**Fe2**		1.0518	1.1515	1.0545	1.0767	1.0696	5.4042
^{1}[**6**]^{0}	**Fe1**		1.0547	1.1315	1.0657	1.0742	1.0853	5.4115
	**Fe2**		1.0553	1.1331	1.0615	1.0749	1.0855	5.4103
^{10}[**6**]$^{+}$	**Fe1**		1.0751	1.1632	1.0896	1.1120	1.1047	5.5445
	**Fe2**		1.0615	1.1286	1.0770	1.0833	1.0807	5.4311

Table S7. B3LYP/TZVP Mössbauer parameters for Fe1 and Fe2 centers of various charged species of **6**

		δ (mm.s^{-1})	ΔE_Q (mm.s^{-1})
^{12}[**6**]$^{+}$	**Fe1**	0.32	0.780
	**Fe2**	0.32	0.753
^{10}[**6**]$^{+}$	**Fe1**	0.28	–0.596
	**Fe2**	0.04	–0.469
^{2}[**6**]$^{+}$	**Fe1**	0.37	1.110
	**Fe2**	0.38	1.494
^{12}[**6**]$^{-}$	**Fe1**	0.33	0.742
	**Fe2**	0.32	0.705
^{10}[**6**]$^{-}$	**Fe1**	0.54	1.888
	**Fe2**	0.54	1.876
^{2}[**6**]$^{-}$	**Fe1**	0.32	0.750
	**Fe2**	0.43	1.865

Table S8. Calculated **g**-tensor and ZFS parameters of various neutral and charged species of **6**

	method	g_x	g_y	g_z	g_{ave}	D (cm^{-1})	E (cm^{-1})
^{11}[**6**]^{0}	**B3LYP***	2.010	2.016	2.017	2.014	0.271	0.005
	**BLYP***	2.012	2.018	2.019	2.016	0.693	0.053
^{12}[**6**]$^{+}$	**B3LYP***	2.009	2.016	2.018	2.014	0.252	0.015
	**BLYP***	2.011	2.018	2.018	2.016	0.252	0.015
^{10}[**6**]$^{+}$	**B3LYP***	2.011	2.021	2.022	2.018	0.250	0.081
	**BLYP***	2.013	2.022	2.023	2.019	0.634	0.047
	**CASSCF+**	2.002	2.002	2.002	2.002	0.100	0.000
	**NEVPT2+**	2.002	2.002	2.002	2.002	0.113	0.000
^{2}[**6**]$^{+}$	**B3LYP***	1.974	1.982	2.101	2.019		
	**BLYP***	1.966	2.008	2.012	1.995		
^{12}[**6**]$^{-}$	**B3LYP***	2.009	2.013	2.014	2.012	0.191	0.006
	**BLYP***	2.011	2.015	2.016	2.014	0.378	0.055
^{10}[**6**]$^{-}$	**B3LYP***	2.024	2.032	2.034	2.030	0.409	0.068
	**BLYP***	2.020	2.022	2.028	2.023	3.968	0.552
^{2}[**6**]$^{-}$	**B3LYP***	1.844	1.952	2.020	1.939		
	**BLYP***	1.956	1.992	2.022	1.990		
	**CASSCF+**	2.002	2.007	2.007	2.005		
	**NEVPT2+**	2.002	2.002	2.002	2.002		

* TZVP basis set

+ def2-SVP basis set

Table S9. Crystallographic data of **6** measured for charge density calculations.

Crystal data	**6**
Empirical formula	$C_{13}H_{17}Cl_4Fe_2N_9O_3Ru$
Formula weight, *Z*	701.93, 4
F(000)	1384.0
Temperature/K	15.0(3)
crystal size/mm	0.136 × 0.048 × 0.036
a/Å	9.5318(2)
b/Å	15.3701(3)
c/Å	15.6352(3)
β/°	93.870(1)
V/Å^3	2285.41(8)
space group	No. 14, $P2_1/n$
wavelength/Å	0.41328
ρ_{calc} g/cm^3, μ/mm^{-1}	2.040, 1.278
scan type	φ scans
2Θ range for data collection/°	2.828 to 55.06
range of indices for *h*, *k*, *l*	–19, +21; –34, +34; –34, +34
No. of measured diffractions	525319
SHELXL (IAM) refinement	
Independent reflections	88734<br>[R_{int} = 0.0433, R_{sigma} = 0.0143]
Data/restraints/parameters	26494/0/291
Goodness-of-fit on F^2	1.066
Final R indexes [I>=2σ (I)]	R_1 = 0.0172, wR_2 = 0.0456
Final R indexes [all data]	R_1 = 0.0187, wR_2 = 0.0460
$\Delta\rho_{max}$, Δρmin/e Å^{-3}	2.23/–1.64
Multipole refinement on F^2	
R(F), wR(F), GOOF	0.0141, 0.0151, 1.9191
R(F^2), wR(F^2), GOOFw	0.0225, 0.0298, 1.9191
Nref/Nv	27.4864
Data/restraints/parameters	24298/0/884
$\Delta\rho_{max}$, Δρmin/e Å^{-3}	1.46, –3.03

Table S10. Selected interatomic distances [Å] and angles [°] in **6**.

	distance		angle
O(1)-N(1)	1.1282(5)	Ru-N(1)-O(1)	175.56(4)
Ru-O(2)	1.9237(4)		
Ru-N(1)	1.7480(4)	O(1)-Ru-O(2)	175.14(2)
Ru-N(2)	2.0412(4)	O(1)-Ru-N(2)	91.70(1)
Ru-N(4)	2.0481(4)	O(1)-Ru-N(4)	96.67(1)
Ru-N(6)	2.0513(4)	O(1)-Ru-N(6)	96.51(1)
Ru-N(8)	2.0547(3)	O(1)-Ru-N(8)	90.51(1)
Fe(1)-Cl(1)	2.2188(1)	N(1)-Ru-N(2)	92.62(2)
Fe(1)-Cl(2)	2.2222(1)	N(1)-Ru-N(4)	95.78(2)
Fe(1)-O(2)	1.9052(4)	N(1)-Ru-N(6)	95.01(2)
Fe(1)-O(3)	1.9756(4)	N(1)-Ru-N(8)	92.04(1)
Fe(1)-N(3)	2.0505(4)	N(1)-Ru-O(2)	171.51(1)
Fe(2)-Cl(3)	2.1962(1)	N(2)-Ru-N(4)	171.51(1)
Fe(2)-Cl(4)	2.2415(1)	N(2)-Ru-N(6)	91.30(2)
Fe(2)-O(2)	1.9322(4)	N(2)-Ru-N(8)	86.96(1)
Fe(2)-O(3)	1.9433(4)	N(4)-Ru-N(6)	89.25(2)
Fe(2)-N(5)	2.0591(4)	N(4)-Ru-N(8)	91.46(1)
		N(6)-Ru-N(8)	172.82(1)
Cl(1)*- O(1)	3.0424(5)		
Cl(2)*- O(1)	3.2561(5)		
Cl(3)**- O(1)	3.0945(5)		
H(6)*** - O(12)	2.857		

Symmetry code used: *) x –½, –y + 3/2, z – ½; **) x – 1, y, z; ***) 1 – x, 1 – y, 1 – z

Table S11. AIM electron density properties at bond critical points in **6**: comparison between experiment (1st row) and density functional theory (experimental geometry) calculations (2nd row).

Bond		BCP characteristics					
Atom 1	Atom 2	d_{12} [Å]	ρ_{BCP} [e/Å^3]	$\nabla^2\rho_{BCP}$ [e/Å^5]	ε	d_1 [Å]	d_2 [Å]
N(1)	O(1)	1.1282	4.20(4)	-29.4(20	0.12	0.6208	0.5074
			4.198	-53.981	0.002		
Ru(1)	N(1)	1.7490	1.210(21)	26.676(35)	0.10	0.9211	0.8279
			1.318	25.524	0.029		
Ru(1)	O(2)	1.9245	0.762(13)	17.626(12)	0.08	1.0124	0.9121
			0.904	14.917	0.008		
Ru(1)	N(2)	2.0427	0.534(11)	14.271(9)	1.88	1.0317	1.0110
			0.750	10.808	0.468		
Ru(1)	N(4)	2.0576	0.576(11)	13.186(8)	1.53	1.0488	1.0088
			0.737	10.694	0.504		
Ru(1)	N(6)	2.0532	0.615(11)	12.875(7)	0.35	1.0563	0.9969
			0.619	9.773	0.466		
Ru(1)	N(8)	2.0606	0.594(10)	12.709(7)	0.42	1.0530	1.0076
			0.622	9.782	0.467		
Fe(1)	Cl(1)	2.2195	0.501(6)	6.898(8)	0.10	1.0318	1.1877
			0.452	5.864	0.031		
Fe(1)	Cl(2)	2.2230	0.436(6)	6.097(9)	0.07	1.0378	1.1852
			0.519	7.136	0.044		
Fe(1)	O(2)	1.9056	0.607(9)	12.969(10)	0.03	0.9439	0.9617
			0.518	10.977	0.127		
Fe(1)	O(3)	1.9770	0.586(7)	10.793(11)	0.05	0.9941	0.9829
			0.561	12.237	0.133		
Fe(1)	N(3)	2.0514	0.547(7)	8.676(10)	0.15	0.9985	1.0529
			0.539	9.828	0.085		
Fe(2)	Cl(3)	2.1963	0.522(6)	7.168(9)	0.13	1.0261	1.1701
			0.549	7.762	0.004		
Fe(2)	Cl(4)	2.2420	0.490(5)	6.414(8)	0.17	1.0496	1.1924
			0.504	6.866	0.039		
Fe(2)	O(2)	1.9323	0.620(8)	12.215(11)	0.02	0.9635	0.9688
			0.624	14.270	0.114		
Fe(2)	O(3)	1.9439	0.609(8)	11.675(10)	0.03	0.9730	0.9710
			0.611	13.765	0.139		
Fe(2)	N(5)	2.0596	0.506(7)	7.914(9)	0.11	1.0035	1.0561
			0.524	9.500	0.103		

Symmetry code used:*) x –½, –y + 3/2, z – ½; **) x – 1, y, z

Table S12. Population of the *d*-orbitals [e-] on central atoms in **6**: comparison between experiment (1st row for each central atom) and density functional theory (experimental geometry).

orbital	$d_{x^2-y^2}$	d_{z^2}	d_{yz}	d_{xz}	d_{xy}	Σ
**Ru1**	0.71(2)	0.79(3)	1.12(2)	1.20(2)	2.07(2)	5.98
**DFT**	0.848	0.741	1.419	1.409	1.949	6.366
**Fe1**	1.46(2)	1.63(2)	1.58(2)	1.28(2)	1.47(2)	7.42
**DFT**	1.174	1.141	1.166	1.241	1.214	5.936
**Fe2**	1.39(2)	1.79(2)	1.37(2)	1.64(2)	1.31(2)	7.50
**DFT**	1.177	1.142	1.161	1.241	1.214	5.936

Table S13. AIM atomic charges and atomic volumes in **6**: comparison between experiment (1st row) and density functional theory (experimental geometry) calculation.

Atom	Ru1	Fe1	Fe2	N1	N2	N4	N6	N8	O1	O2	O3	Cl1	Cl2	Cl3	Cl4
								charge [e^-]							
**experiment**	2.11	1.39	1.33	0.04	-0.56	-0.65	-0.55	-0.48	-0.34	-0.91	-1.12	-0.49	-0.66	-0.71	-0.49
**DFT**	1.608	1.656	1.678	0.232	-0.849	-0.853	-0.773	-0.764	-0.451	-1.117	-1.145	-0.588	-0.606	-0.580	-0.609
**experiment** *V001* [Å^3]	10.31	11.31	11.63	12.10	9.41	10.03	10.61	10.53	16.76	12.11	12.76	34.36	33.55	34.02	32.71
**DFT, iso = 0.0004 au**	11.62	9.62	9.72	12.34	9.83	10.44	11.57	11.33	20.79	12.71	13.02	40.82	40.12	39.58	39.04

Table S14. Crystal data and details of data collection for 3, 4, 5·H₂O, 6, 7·MeOH and 8.

Compound	**3**	**4**	**5·H_2O**	**6**	**7·MeOH**	**8**
empirical formula	$C_{12}H_{16}Cl_2N_8Ru$	$C_{24}H_{28}MgN_{18}O_4Ru_2$	$C_{12}H_{19}Cl_2N_9O_3Ru$	$C_{13}H_{17}Cl_4Fe_2N_9O_3Ru$	$C_{31}H_{47}Cl_6Fe_4N_{19}O_9Ru_2$	$C_{15}H_{21}Cl_3Ga_3N_9O_5Ru$
fw	444.30	859.10	509.33	701.93	1468.12	823.99
space group	*C*2/*c*, no. 15	*P*-1, no. 2	$P2_1/n$, no. 14	$P2_1/n$, no. 14	*Cc*, no. 9	$P2_1/n$, no. 14
a [Å]	13.8222(5)	8.7988(4)	9.4185(7)	9.6588(3)	37.6143(6)	13.7228(18)
b [Å]	9.1828(3)	9.6040(4)	14.426(2)	15.5863(5)	8.8399(2)	12.1107(8)
c [Å]	14.0063(5)	10.4046(4)	15.5176(14)	15.8342(5)	17.6163(5)	16.3322(9)
α		87.0450(12)				
β [°]	116.785(1)	71.7653(13)	107.485(2)	93.7777(16)	116.304(2)	98.021(4)
γ		83.4896(13)				
V [Å^3]	1587.02(10)	829.57(6)	2010.9(4)	2378.58(13)	5251.0(2)	2687.7(4)
Z	4	1	4	4	4	4
λ [Å]	0.71073	0.71073	0.71073	1.54178	0.71073	0.71073
ρ_{calcd} [g cm^{-3}]	1.860	1.720	1.682	1.960	1.857	2.036
crystal size [mm]	0.20 × 0.20 × 0.09	0.15 × 0.07 × 0.04	0.07 × 0.05 × 0.05	0.10 × 0.02 × 0.02	0.25 × 0.03 × 0.03	0.10 × 0.04 × 0.04
T [K]	100(2)	100(2)	100(2)	100(2)	100(2)	100(2)
μ [mm^{-1}]	1.336	0.991	1.078	19.189	2.008	3.869
R_1[a]	0.0234	0.0193	0.0846	0.0259	0.0417	0.0538
wR_2[b]	0.0551	0.0463	0.2809	0.0633	0.1033	0.1401
GOF[c]	1.067	1.098	1.062	1.027	1.090	1.020

[a] $R_1 = \Sigma||F_o| - |F_c||/\Sigma|F_o|$. [b] $wR_2 = \{\Sigma[w(F_o^2 - F_c^2)^2]/\Sigma[w(F_o^2)^2]\}^{1/2}$. [c] GOF $= \{\Sigma[w(F_o^2 - F_c^2)^2]/(n - p)\}^{1/2}$, where *n* is the number of reflections and *p* is the total number of parameters refined.

References

(1) Králík, F.; Vřešťál, J. Komplexverbindungen des Rutheniums mit Fünfzähligen Heterocyclischen Basen. II. Reaktionen des Ruthenium(III)-Chlorids mit Pyrazol und Imidazol. *Collect. Czech. Chem. Commun.* **1961**, *26*, 1298–1304.

(2) Bučinský, L.; Büchel, G. E.; Ponec, R.; Rapta, P.; Breza, M.; Kožíšek, J.; Gall, M.; Biskupič, S.; Fronc, M.; Schiessl, K.; Cuzan, O.; Prodius, D.; Turta, C.; Shova, S.; Zając, D. A.; Arion, V. B. On the Electronic Structure of *mer*,*trans*-[$RuCl_3$(1*H*-Indazole)$_2$(NO)], a Hypothetical Metabolite of the Antitumor Drug Candidate KP1019: An Experimental and DFT Study. *Eur. J. Inorg. Chem.* **2013**, 2505–2519.

(3) Reisner, E.; Arion, V. B.; Eichinger, A.; Kandler, N.; Giester, G.; Pombeiro, A. J. L.; Keppler, B. K. Tuning of Redox Properties for the Design of Ruthenium Anticancer Drugs: Part 2. Syntheses, Crystal Structures, and Electrochemistry of Potentially Antitumor [$Ru^{III/II}Cl_{6-n}$(Azole)$_n$]^z (n = 3, 4, 6) Complexes. *Inorg. Chem.* **2005**, *44*, 6704–6716.

(4) Hübschle, C. B.; Dittrich, B. MoleCoolQt – a Molecule Viewer for Charge-Density Research. *J. Appl. Crystallogr.* **2011**, *44*, 238–240.
